# Evaluation of Simvastatin as a Disease-Modifying Treatment for Patients With Parkinson Disease: A Randomized Clinical Trial

**DOI:** 10.1001/jamaneurol.2022.3718

**Published:** 2022-10-31

**Authors:** Kara N. Stevens, Siobhan Creanor, Alison Jeffery, Alan Whone, John Zajicek, Andy Foggo, Ben Jones, Rebecca Chapman, Laura Cocking, Jonny Wilks, Doug Webb, Camille Carroll

**Affiliations:** 1 Faculty of Health, University of Plymouth, Plymouth, United Kingdom; 2 Exploristics Ltd, Belfast, United Kingdom; 3 College of Medicine and Health, University of Exeter, Exeter, United Kingdom; 4 Bristol Medical School, University of Bristol, Bristol, United Kingdom; 5 School of Medicine, Medical and Biological Sciences, University of St Andrews, St Andrews, United Kingdom; 6 School of Biological and Marine Sciences, Faculty of Science and Engineering, University of Plymouth, Plymouth, United Kingdom; 7 NIHR BioResource, University of Cambridge, Cambridge, United Kingdom; 8 MAC Clinical Research, Blackpool, United Kingdom; 9 Bristol Trials Centre, University of Bristol, Bristol, United Kingdom

## Abstract

**Importance:**

Current treatments manage symptoms of Parkinson disease (PD), but no known treatment slows disease progression. Preclinical and epidemiological studies support the potential use of statins as disease-modifying therapy.

**Objective:**

To determine whether simvastatin has potential as a disease-modifying treatment for patients with moderate PD.

**Design, Setting, and Participants:**

This randomized clinical trial, a double-blind, parallel-group, placebo-controlled futility trial, was conducted between March 2016 and May 2020 within 23 National Health Service Trusts in England. Participants aged 40 to 90 years with a diagnosis of idiopathic PD, with a modified Hoehn and Yahr stage of 3.0 or less while taking medication, and taking dopaminergic medication with wearing-off phenomenon were included. Data were analyzed from May 2020 to September 2020, with additional analysis in February 2021.

**Interventions:**

Participants were allocated 1:1 to simvastatin or matched placebo via a computer-generated random sequence, stratified by site and Hoehn and Yahr stage. In the simvastatin arm, participants entered a 1-month phase of simvastatin, 40 mg daily, followed by 23 months of simvastatin, 80 mg daily, before a 2-month washout period.

**Main Outcomes and Measures:**

The prespecified primary outcome was 24-month change in Movement Disorder Society Unified Parkinson Disease Rating Scale (MDS-UPDRS) part III score measured while not taking medication (high scores indicate worse outcome). The primary futility analysis included participants who commenced the 80-mg phase and had valid primary outcome data. The safety analysis included all participants who commenced trial treatment and is reported by dose at time of event.

**Results:**

Of 332 patients assessed for eligibility, 32 declined and 65 were ineligible. Of 235 recruited participants, 97 (41%) were female, 233 (99%) were White, and the mean (SD) age was 65.4 (9.4) years. A total of 216 patients progressed to the 80-mg dose. Primary outcome analysis (n = 178) indicated the simvastatin group had an additional deterioration in MDS-UPDRS III score while not taking medication at 24 months compared with the placebo group (1.52 points; 2-sided 80% CI, −0.77 to 3.80; 1-sided futility test *P* = .006). A total of 37 serious adverse events (AEs), including 3 deaths, and 171 AEs were reported for participants receiving 0-mg simvastatin; 37 serious AEs and 150 AEs were reported for participants taking 40 mg or 80 mg of simvastatin. Four participants withdrew from the trial because of an AE.

**Conclusions and Relevance:**

In this randomized clinical trial, simvastatin was futile as a disease-modifying therapy in patients with PD of moderate severity, providing no evidence to support proceeding to a phase 3 trial.

**Trial Registration:**

ISRCTN Identifier: 16108482

## Key Points

**Question:**

Does simvastatin hold promise as a disease-modifying therapy in patients with Parkinson disease of moderate severity?

**Findings:**

In this randomized clinical trial, a double-blind, parallel-group, placebo-controlled futility trial involving 235 participants from 23 sites within the UK, participants in the simvastatin group had an additional deterioration in Movement Disorder Society Unified Parkinson Disease Rating Scale part III scores while not taking medication at 24 months compared with those in the placebo group (−1.52 points).

**Meaning:**

In this randomized clinical trial, simvastatin was futile as a disease-modifying therapy in patients with moderate Parkinson disease.

## Teaser

This randomized clinical trial evaluates whether simvastatin has potential as a disease-modifying treatment for patients with moderate Parkinson disease.

## Introduction

Parkinson disease (PD) is the fastest growing neurological condition worldwide,^1^ currently affecting more than 6.2 million people. To date, there is no treatment proven to slow disease progression.

Preclinical studies suggest that statins, widely used to treat hypercholesterolemia, may have disease-modifying effects relevant to the pathogenesis of PD unrelated to cholesterol lowering.^2^ Much of this evidence relates to simvastatin, which is one of the most lipophilic statins and able to cross the blood-brain barrier.^3,4^ Epidemiological studies support a potential protective effect of statins on PD, with meta-analyses demonstrating statin use may be associated with a relative risk reduction in PD incidence.^5,6,7^

Our aim was to assess the potential disease-modifying effects of 24 months’ exposure to simvastatin in patients with moderate PD and to determine if simvastatin is clearly ineffective (futile) in preventing the clinical decline of PD.

## Methods

### Study Design, Setting, and Participants

The study was a double-blind, parallel-group, randomized, placebo-controlled futility trial. The protocol was previously published,^8^ and the trial protocol can be found in Supplement 1. Participants were recruited from Parkinson services within 23 National Health Service (NHS) Trusts in England (eAppendix in Supplement 3). Patients were eligible if they were aged 40 to 90 years with a diagnosis of idiopathic PD, had a modified Hoehn and Yahr stage of 3.0 or less while taking medication, and were taking dopaminergic medication with wearing-off phenomenon (defined by the 9-item wearing-off questionnaire^9^). Patients were excluded if they had a diagnosis or suspicion of a secondary cause of parkinsonism; clinically relevant brain imaging abnormality; dementia; severe depression; prior intracerebral surgical intervention; intolerance, prior, or current use of statins; untreated hypothyroidism; end-stage kidney disease; severe cardiac disease; estimated glomerular filtration rate less than 30 mL/min; alcoholism or liver impairment; raised creatine kinase or aspartate/alanine transaminase levels; pregnancy; breastfeeding; participation in high-impact sports; or inability to abstain from grapefruit-based products. Ethnicity was defined by investigators and assessed to facilitate description of the study sample. Details of evaluation of cardiovascular risk, including new diagnosis of diabetes, are given in the trial protocol.^8^ Ethical approval was obtained from the North East–Newcastle and North Tyneside 2 Research Ethics Committee. All participants provided written informed consent. This study followed the Consolidated Standards of Reporting Trials (CONSORT) reporting guideline.

### Procedures

Participants were invited to attend face-to-face visits at baseline and months 1, 6, 12, 18, 24, and 26 (within 2 weeks) postbaseline, with 1 telephone call every 2 months to capture adverse events (AEs). Participants attended visits at baseline, 12, 24, and 26 months in the practically defined state of not taking medication (short-acting levodopa-containing medications and/or dopamine agonists omitted from 6 pm the evening before the study visit and long-acting preparations, including monoamine oxidase inhibitors, for the entire previous day).

Lockdown restrictions related to COVID-19 were introduced in the United Kingdom on March 23, 2020; remaining visits were conducted remotely using video conferencing. Where necessary, data were collected via telephone or mail.

### Allocation and Concealment

Allocation 1:1 to simvastatin or placebo, stratified by recruiting site and Hoehn and Yahr stage (either stage 2.0 or less or stage 2.5 to 3.0) used computer-generated random permuted blocks of size 2, 4, or 6. The randomization process generated a study-specific prescription of the participant’s study number, initials, and allocated bottle number for the relevant hospital pharmacy to dispense study medication.

Participants, trial management team, site investigator teams (including outcome raters), and site pharmacy staff were blinded to treatment allocation. Primary statistical analyses of the primary outcomes were undertaken blinded.

### Interventions

Trial treatment was overencapsulated simvastatin, 40 mg oral, tablets or visually identical matched placebo. A 1-month low-dose phase of simvastatin, 40 mg, once daily was followed by a 23-month high-dose phase of simvastatin, 80 mg, once daily and a final 2-month phase without trial medication. In the event of AEs thought to be related to trial medication, the participant’s dose could be reduced to 40 mg, then increased to 80 mg at a later date.

### Outcomes

The primary outcome was change between baseline and 24 months in the Movement Disorder Society Unified Parkinson Disease Rating Scale (MDS-UPDRS)^10^ part III, measured in the practically defined state of not taking medication (higher scores indicate worse outcome). Other assessments while not taking medication were the Bradykinesia Akinesia Incoordination Tap Test (number of alternate key strikes [*s* and *;*] in 30 seconds) and 10-meter walk test. Remaining secondary outcomes were measured while participants were taking medication, including MDS-UPDRS total and part II score, Montgomery-Åsberg Depression Rating Scale score, Non-Motor Symptom Scale score, Parkinson Disease Questionnaire score, King’s Parkinson Pain Scale score (higher scores worse), Addenbrooke’s Cognitive Examination III (ACE-III) score (lower scores worse), levodopa-equivalent daily dose (LEDD), and cholesterol levels (high-density lipoprotein [HDL] cholesterol, total cholesterol, and total/HDL cholesterol ratio). The visit and outcome schedule is detailed in the published trial protocol^8^ and in Supplement 1.

Safety and tolerability data were gathered via telephone and during follow-up visits. The number of capsules returned was recorded at months 1, 6, 12, 18, and 24.

### Sample Size

The sample size calculation was based on a 1-sided *t* test of futility at the 10% significance level with 80% power.^11^ At the time of study development, the minimal clinically important difference (MCID) of UPDRS part III score while not taking medication was estimated to be between 2.3 and 2.7 points^12^; therefore, a target difference of −3 was deemed clinically relevant. The anticipated SD was inflated from a reported 7.3^13^ to 7.5 points, yielding an effective required sample size of 57 participants per group. This was inflated for initial, potentially differential, loss to follow-up during the 1-month low-dose phase and then for further loss to follow-up by 24 months, giving a total recruitment target of 198 (Supplement 1).^8^

### Statistical Analysis

Predefined statistical analyses are fully detailed in the statistical analysis plan, which has been previously published^14^ and can be found in Supplement 2. Primary analyses followed a modified intention-to-treat principle (mITT). The mITT sample included all randomized participants who commenced the higher-dose phase at 1 month and provided valid outcome measurement data. Missing outcome/questionnaire items were imputed using published methods and imputation rules. An error in the researcher booklet for item 15 of the MDS-UPDRS part III (postural tremor of the hands) left space for 1 score instead of 2. Therefore, imputation was restricted to a maximum of 3 missing items (Supplement 2).

A mixed-effects linear regression model was fitted to the 24-month change in the MDS-UPDRS part III score while not taking medication (primary outcome), with adjustments for baseline outcome, sex, age at baseline, PD duration, and modified Hoehn and Yahr stage^15,16^ and a random intercept for recruiting site. The primary analysis of the primary outcome was based on the futility hypothesis test of the allocated group difference estimate from this mixed-effects model, under the null hypothesis of simvastatin arm score minus placebo arm score is −3 or less (ie, not futile) vs greater than −3 (ie, futile). To test for futility, the estimated adjusted mean between-group difference was compared with the 1-sided upper tail critical value of *t* test distribution.^17^ Planned sensitivity futility analyses included complier average causal effect analysis and treatment compliance (Supplement 2).

There were 2 planned secondary analyses of the primary outcome: (1) a mixed-effects repeated-measures model fitted to the MDS-UPDRS part III score while not taking medication at baseline, 12, and 24 months, testing between-group differences at follow-up under the superiority framework; and (2) an exploratory analysis of any disease-modifying effect of simvastatin if there was evidence of nonfutility in primary outcome analysis (Supplement 2). A post hoc futility analysis was conducted for the 12-month change in MDS-UPDRS part III score, following the observed (before unblinding) statistically significant between-group difference at 12 months from the repeated-measures model.

Analyses of the secondary outcomes were performed under the superiority framework (ie, 2-sided hypotheses, 5% significance level) using linear mixed-effects models for (1) the outcome at 24 months and (2) the repeated measures of the outcome. Planned sensitivity analyses are detailed in the statistical analysis plan (Supplement 2).

The safety population consisted of all participants who had at least 1 dose of allocated trial treatment. All AEs considered possibly, probably, or definitely related to trial treatment and all serious AEs were reported. As AEs of simvastatin are generally related to plasma levels of simvastatin and extent of 3-hydroxy-3-methylglutaryl coenzyme A reductase activity, the safety reporting groups were defined by dose of simvastatin at the time of the event. Accounting for the elimination half-life of 2 hours, these are 0 mg if the participant was allocated to placebo or the participant had discontinued simvastatin more than 24 hours prior to event (more than 5 half-lives); 40 mg if the event observed during the simvastatin, 40 mg, phase and participant was taking simvastatin, 40 mg, or 24 hours after reducing simvastatin dose from 80 mg to 40 mg; and 80 mg if the event was observed while taking simvastatin, 80 mg, or within 24 hours of discontinuing simvastatin, 80 mg. The safety data are summarized as frequency of cumulative incidence. Analyses were performed in Stata SE version 14 (StataCorp) and independently double-coded in R version 4.0.2 (The R Foundation).

## Results

**Figure 1.**
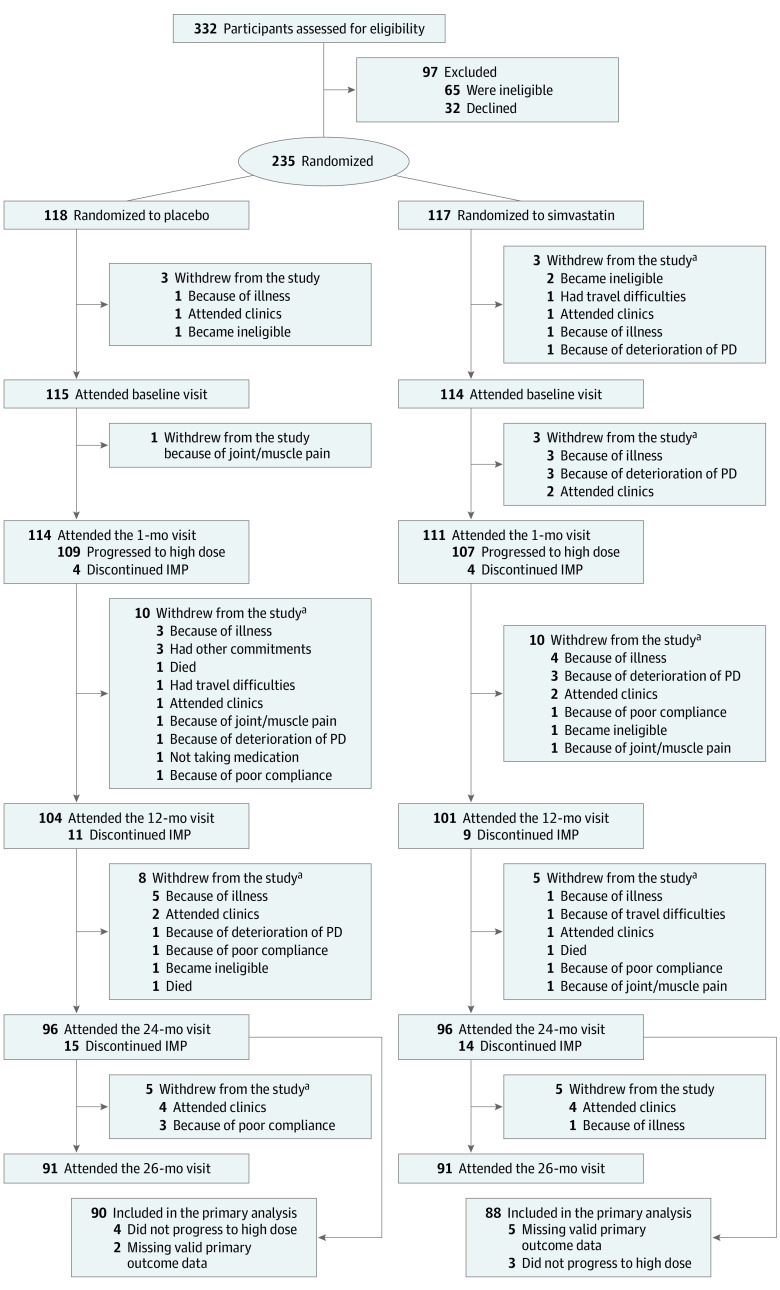
CONSORT Diagram of the PD STAT Study One participant who did not progress to the high dose was also missing valid primary outcome data. IMP indicates investigational medicinal product; PD, Parkinson disease. ^a^Multiple reasons provided for withdrawal.

Recruitment was between March 1, 2016, and March 31, 2018. Of 332 patients screened, 235 were randomized; 117 were allocated to simvastatin and 118 to placebo (Figure 1; eTable 1 in Supplement 3). Of 332 patients assessed for eligibility, 32 declined and 65 were ineligible. Of 235 recruited participants, 97 (41%) were female, 233 (99%) were White, and the mean (SD) age was 65.4 (9.4) years. Of the 228 participants who commenced trial treatment, 216 progressed to the high-dose phase at 1 month (evaluable sample; baseline characteristics summarized in Table 1). At the primary end point (24 month), 178 of 216 (82.4%) of the mITT sample remained in the study, including 88 of 107 (82.2%) in the simvastatin group and 90 of 109 (82.6%) in placebo group.

**Table 1.** Summary Statistics of the Baseline Characteristics by Allocated Group of All Participants Who Progressed to the High-Dose Phase at 1 Month

Characteristic	No. (%)		
	Placebo (n = 109)	Simvastatin (n = 107)	Total (N = 216)
Age, mean (SD), y	64.8 (9.9)	65.9 (8.6)	65.3 (9.3)
Sex			
Female	49 (45)	40 (37)	89 (41)
Male	60 (55)	67 (63)	127 (59)
Disease duration, mean (SD), y	9.8 (4.6)	9.5 (3.8)	9.7 (4.2)
Systolic blood pressure, mean (SD), mm Hg	128.9 (17.9)	125.7 (16.3)	127.3 (17.2)
BMI, mean (SD)^a^	26.8 (4.7)	26.2 (4.6)	26.5 (4.7)
MoCA score, mean (SD)	27.6 (2.4)	27.6 (2.2)	27.6 (2.3)
Ethnicity^b^			
White	108 (99)	106 (99)	214 (99)
Other	1 (1)	1 (1)	2 (1)
Smoking status			
Never	76 (70)	71 (66)	147 (68)
Ex-smoker	30 (27)	32 (30)	62 (29)
Light	3 (3)	3 (3)	6 (3)
Moderate	0	1 (1)	1 (0.5)
Relationship status			
Single	7 (6)	5 (5)	12 (6)
Married or civil partnership	89 (82)	90 (84)	179 (83)
Separated	3 (3)	1 (1)	4 (2)
Divorced or partnership dissolved	6 (6)	7 (7)	13 (6)
Widowed or surviving partner	4 (4)	4 (4)	8 (4)
Living status			
Live alone	10 (9)	12 (11)	22 (10)
Live with spouse or partner	95 (87)	92 (86)	187 (87)
Live with parent(s)	1 (1)	0	1 (0.5)
Live with children			
<18 y	10 (9)	13 (12)	23 (11)
≥18 y	17 (16)	14 (13)	31 (14)
Live with nonfamily	1 (1)	1 (1)	2 (1)
QRISK2 risk score			
≥10%	51 (47)	49 (46)	100 (46)
Median (IQR)	9.2 (3.7-16.0)	8.6 (5.8-15.9)	9.0 (4.7-16.0)
Cholesterol, mean (SD), mg/dL			
HDL	61.8 (19.3)	61.8 (19.3)	61.8 (19.3)
Total	189.2 (42.5)	189.2 (38.6)	189.2 (42.5)
Total/HDL ratio	3.2 (1.0)	3.3 (1.0)	3.2 (1.0)
Hoehn and Yahr stage			
1.0-2.0	74 (68)	75 (70)	149 (69)
2.5-3.0	35 (32)	32 (30)	67 (31)

Abbreviations: BMI, body mass index; HDL, high-density lipoprotein; MoCA, Montreal Cognitive Assessment.

SI conversion factor: To convert cholesterol to mmol/L, multiply by 0.0259.

^a^ Calculated as weight in kilograms divided by height in meters squared.

^b^ Ethnicity was defined by investigators and assessed to facilitate description of the study sample. The other category included Indian and unreported.

**Figure 2.**
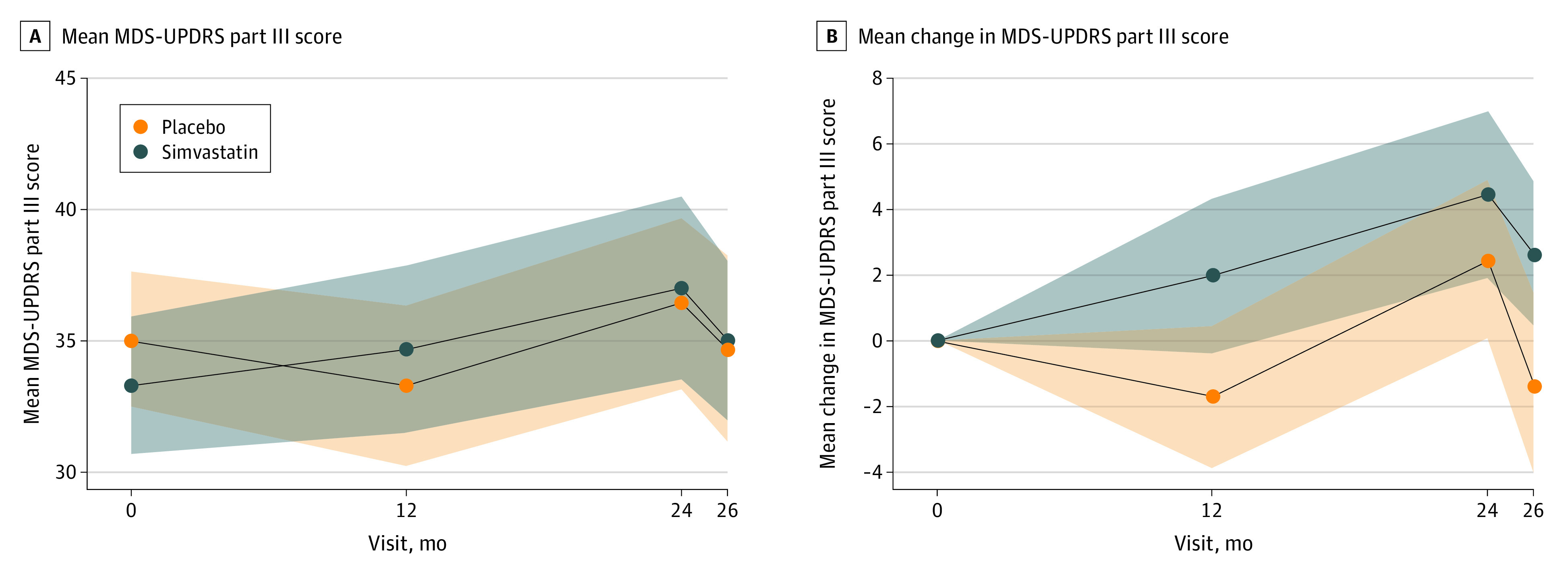
Mean Movement Disorder Society Unified Parkinson Disease Rating Scale (MDS-UPDRS) Part III Score by Allocated Group MDS-UPDRS part III score while not taking medication and mean of the change in MDS-UPDRS part III score from baseline to follow-up time point, including participants who progressed to the high dose at 1 month who had valid outcome data at each time point.

Figure 2 shows, by allocated group, the mean of the MDS-UPDRS part III score while not taking medication at each time point and the mean change between baseline and follow-up time points with 95% CIs. At 12 months, mean (SD) change in MDS-UPDRS part III score from baseline was −1.7 (10.9) in the placebo group and 2.0 (11.8) in the simvastatin group (Figure 2; Table 2). At 24 months (primary end point), the mean (SD) change was 2.4 (11.2) in the placebo group and 4.5 (12.2) in the simvastatin group. The fully adjusted between-group difference (simvastatin minus placebo) in the 24-month change of MDS-UPDRS part III score indicated that participants in the simvastatin group had worsened, on average, by an additional 1.52 points (2-sided 80% CI, −0.77 to 3.80) compared with the placebo group. The test of the futility hypothesis indicated that simvastatin was futile as a treatment for PD (*P* = .006). All prespecified sensitivity analyses of the primary outcome indicated that simvastatin was futile (Table 2). As there was no evidence that simvastatin was nonfutile, a disease-modifying analysis, including the 26-month data, was not performed.

**Table 2.** Results of the Fully Adjusted Linear Regression Mixed-Effects Models^a^

Analysis	Mean between-group difference (2-sided 80% CI)	Futility test *P* value
Primary analysis		
24-mo Change in MDS-UPDRS part III score in mITT population	1.52 (−0.77 to 3.8)	.003
Sensitivity analyses		
24-mo Change in MDS-UPDRS part III score		
Excluding outliers in the mITT population	0.82 (−1.08 to 2.72)	.006
Visit location in the non–COVID-19 population	1.62 (−0.70 to 3.93)	.006
Unrestricted imputation		
mITT population	1.10 (−1.16 to 3.36)	.01
COVID-19 population	1.51 (−0.78 to 3.81)	.007
CACE		
Compliance (i)	2.17 (−1.29 to 5.64)	.03
Compliance (ii)	2.08 (−1.26 to 5.42)	.03
Treatment compliance^b^	1.74 (−1.03 to 4.51)	.02
Post hoc analysis		
12-mo Change in MDS-UPDRS part III score		
mITT population^b^	2.74 (0.70 to 4.77)	<.001
Excluding outliers	2.55 (0.75 to 4.35)	<.001

Abbreviations: CACE, complier average causal effect; mITT, modified intention to treat.

^a^ Adjusted for baseline, age, sex, Parkinson disease duration, Hoehn and Yahr stage as fixed effects and study center as a random effect, with futility tests of the primary outcome for the primary, planned sensitivity, and post hoc analyses.

^b^ Post hoc analysis.

The a priori repeated measures model of MDS-UPDRS part III score while not taking medication, under superiority assumptions, showed a mean between-group difference of 2.74 (95% CI, −0.11 to 5.58; *P* = .06) at 12 months and 1.17 (95% CI, −1.78 to 4.13; *P* = .43) at 24 months. This prompted a post hoc blinded futility analysis of the 12-month change in MDS-UPDRS part III score, which indicated that the simvastatin group had deteriorated, on average, by 2.74 points (80% CI, 0.75-4.35; *P* < .001) more than the placebo group. However, in a (planned) superiority analysis, the between-group difference of the 12-month change in MDS-UPDRS part III score was not statistically significant (mean difference in changes, 2.74; 95% CI, −0.38 to 5.85; *P* = .09) (Table 3).

**Table 3.** Summary Statistics of Those Who Progressed to the High-Dose Phase at 1 Month With Valid Outcome Measurement by Visit Month^a^

Visit, mo	Placebo		Simvastatin		Fully adjusted mean between-group difference (2-sided 95% CI)	
	Total, No.	Mean (SD)	Total, No.	Mean (SD)	24-mo Linear regression model	Repeated-measures model
MDS-UPDRS part III score while not taking medication^b^						
0	109	35.1 (13.8)	107	33.3 (13.7)	NA	NA
12	97	33.3 (15.5)	97	34.7 (16.0)	NA	2.74 (−0.11 to 5.58)
24	90	36.4 (15.9)	88	37.0 (16.7)	NA	1.17 (−1.78 to 4.13)
26	72	34.7 (15.4)	68	35.0 (12.8)	NA	NA
HDL cholesterol, mg/dL						
0	109	61.8 (19.3)	107	61.8 (19.3)	NA	NA
12	93	61.8 (23.2)	96	61.8 (15.4)	NA	−0.05 (−0.18 to 0.07)^c^
24	83	65.6 (27.0)	86	61.8 (23.2)	0.001 (−0.14 to 0.14)^c^	0.01 (−0.16 to 0.14)^c^
26	68	57.9 (19.3)	68	57.9 (15.4)	NA	NA
Total cholesterol, mg/dL						
0	109	189.2 (42.5)	107	189.2 (38.6)	NA	NA
12	97	193.1 (34.8)	97	135.1 (34.8)	NA	−1.47 (−1.70 to −1.25)
24	87	185.3 (42.5)	88	139.0 (38.6)	−1.15 (−1.41 to −0.88)	−1.15 (−1.38 to −0.91)
26	70	193.1 (1.2)	69	189.2 (38.6)	NA	NA
Total/HDL cholesterol ratio						
0	109	3.2 (1.0)	107	3.3 (1.0)	NA	NA
12	93	3.3 (1.0)	96	2.3 (0.7)	NA	−0.99 (−1.19 to −0.78)
24	83	3.1 (1.0)	86	2.4 (0.9)	−0.69 (−0.90 to −0.48)	−0.70 (−0.91 to −0.49)
26	68	3.5 (1.0)	68	3.4 (1.0)	NA	NA
Bradykinesia Akinesia Incoordination Tap Test while not taking medication^b^^,^^d^						
0	91	45.2 (12.0)	92	46.1 (11.9)	NA	NA
12	85	44.9 (12.5)	81	45.4 (13.9)	NA	0.25 (−2.20 to 2.72)
24	74	44.5 (13.1)	72	44.1 (14.0)	−1.25 (−4.35 to 1.85)	−1.58 (−4.15 to 0.99)
26	66	44.3 (12.6)	56	43.2 (14.1)	NA	NA
Mean 10-minute walk test time while not taking medication, s^b^						
0	108	6.8 (10.3)	105	7.7 (11.6)	NA	NA
12	94	5.7 (3.5)	95	6.2 (12.4)	NA	−0.69 (−2.94 to 1.57)^c^
24	84	6.3 (5.5)	79	5.5 (3.1)	−0.64 (−1.61 to 0.33)^c^	−0.88 (−2.91 to 1.14)^c^
26	73	6.0 (3.8)	66	6.2 (5.0)	NA	NA
10-Minute walk test completed at least 1 test while not taking medication, No. (%)^b^						
0	109	108 (99)	107	105 (98)	NA	NA
12	97	94 (97)	97	95 (98)	NA	NA
24	91	84 (92)	94	79 (96)	Odds ratio, 0.50 (0.18 to 1.43)	NA
26	87	73 (84)	86	66 (77)	NA	NA
MDS-UPDRS part II score^b^						
0	108	10.8 (7.1)	106	10.6 (6.0)	NA	NA
12	100	12.1 (8.1)	91	11.7 (7.4)	NA	0.03 (−1.30 to 1.23)
24	85	11.4 (7.1)	90	13.1 (8.6)	0.97 (−0.52 to 2.46)	1.07 (−0.25 to 2.39)
26	83	12.3 (7.5)	85	12.6 (8.5)	NA	NA
MDS-UPDRS total score while taking medication^b^						
0	107	46.3 (21.5)	106	45.9 (18.3)	NA	NA
12	99	47.8 (23.5)	91	50.0 (21.4)	NA	2.89 (−0.98 to 6.77)
24	85	49.1 (22.9)	87	51.6 (22.1)	0.45 (−4.11 to 5.01)	0.66 (−3.39 to 4.70)
26	70	53.2 (24.9)	69	48.7 (17.5)	NA	NA
PDQ-39 total score^b^						
0	99	16.3 (12.0)	98	14.4 (10.2)	NA	NA
12	81	19.0 (14.7)	79	15.9 (11.5)	NA	−1.98 (−4.46 to 0.51)
24	73	18.2 (14.0)	76	17.1 (11.8)	0.41 (−2.41 to 3.22)	−0.05 (−2.51 to 2.60)
26	70	18.7 (14.6)	68	14.8 (10.8)	NA	NA
NMSS total score^b^						
0	108	39.1 (31.4)	104	38.9 (31.1)	NA	NA
12	96	41.7 (30.9)	94	38.9 (29.3)	NA	−1.98 (−9.30 to 5.35)^c^
24	88	37.9 (29.9)	88	40.9 (27.4)	3.11 (−3.77 to 9.98)^c^	3.02 (−4.18 to 10.22)^c^
26	86	37.7 (34.1)	83	39.8 (29.0)	NA	NA
KPPS total score^b^						
0	108	12.7 (13.0)	106	11.1 (11.8)	NA	NA
12	99	13.5 (13.9)	98	12.1 (14.5)	NA	0.12 (−3.06 to 3.29)^c^
24	91	13.5 (15.0)	92	13.1 (13.7)	0.72 (−2.35 to 3.78)^c^	0.49 (−3.16 to 4.15)^c^
26	87	13.3 (15.6)	86	13.0 (12.3)	NA	NA
LEDD, mg						
0	109	606.4 (302.5)	107	608.6 (286.2)	NA	NA
12	100	729.1 (359.6)	98	646.6 (311.6)	NA	−83.40 (−139.68 to −27.12)
24	91	734.0 (344.5)	93	744.0 (351.8)	−18.99 (−87.32 to 49.34)	−6.35 (−64.38 to 51.67)
26	87	756.7 (372.8)	87	749.9 (375.1)	NA	NA
Hemoglobin A_1c_, mmol/mol						
0	109	36.5 (3.9)	107	36.3 (4.6)	NA	NA
24	84	36.9 (3.9)	86	37.1 (4.1)	0.31 (−0.46 to 1.08)	NA
						
						
Diabetes						
0	109	0	107	1 (0.9)	NA	NA
24	84	1 (1.2)	86	1 (1.2)	Odds ratio, 0.86 (0.05 to 14.6)	NA
ACE-III total score						
0	109	93.8 (6.1)	105	94.6 (4.5)	NA	NA
12	98	93.4 (7.5)	96	94.1 (5.6)	NA	0.58 (−0.73 to 1.88)^c^
24	89	94.2 (5.6)	85	94.0 (5.9)	−0.11 (−1.28 to 1.07)^c^	0.10 (−1.00 to 1.20)^c^
26	73	94.7 (6.5)	73	93.3 (8.1)	NA	NA
MADRS total score^b^						
0	109	7.3 (5.8)	107	6.0 (5.2)	NA	NA
12	99	7.6 (6.2)	97	6.7 (6.1)	NA	−0.26 (−1.62 to 1.11)
24	90	6.9 (5.7)	92	7.1 (5.3)	−0.62 (−0.79 to 2.04)	0.69 (−0.73 to 2.10)
26	86	6.7 (7.3)	85	7.4 (5.6)	NA	NA

Abbreviations: ACE, Addenbrooke Cognitive Examination; HDL, high-density lipoprotein; KPPS, King’s Parkinson Disease Pain Scale; LEDD, levodopa-equivalent daily dose; MADRS, Montgomery-Åsberg Depression Rating Scale; MDS-UPDRS, Movement Disorder Society Unified Parkinson Disease Rating Scale; NA, not applicable; NMSS, Non-Motor Symptom Scale; PDQ-39, Parkinson Disease Questionnaire.

SI conversion factor: To convert cholesterol to mmol/L, multiply by 0.0259.

^a^ All models were adjusted for age and PD duration at baseline, sex, and Hoehn and Yahr stage as fixed effects and study center as random effects. In addition, the 24-month model included the corresponding outcome measure at baseline as a fixed effect and the repeated-measures model included participants nested within study center as a random effect.

^b^ Higher scores indicates worse outcome.

^c^ CIs calculated from bootstrapped SE estimate.

^d^ Bradykinesia Akinesia Incoordination Tap Test measured as frequency of key presses in 30 seconds.

Summary statistics for the secondary outcomes are shown in Table 3. Where modeling assumptions were violated (mean 10-minute walk test time, HDL cholesterol level, Non-Motor Symptom Scale, and King’s Parkinson Pain Scale), mean between-group estimates are presented on the original scale with bootstrapped CIs; conclusions were confirmed by fitting both models to the log-transformed outcome data. Conclusions regarding the statistical significance of the 24-month between-group difference were the same for both sets of analyses (linear regression and repeated measures) of all secondary outcomes. The expected reductions in total cholesterol level and total/HDL cholesterol ratio in the simvastatin group were observed. There was evidence of a between-group difference in LEDD at 12 months; mean LEDD in the simvastatin group was 83.4 mg (95% CI, 27.1-139.7; *P* = .004) lower than in the placebo group. There were no other statistically significant between-group differences at 12 or 24 months. Planned sensitivity analyses of the secondary outcomes of the non–COVID-19 population yielded similar results to the mITT population (eTable 2 in Supplement 3).

There were 74 SAEs reported by 49 participants who commenced trial treatment (eTable 3 in Supplement 3). No SAEs occurred while participants were taking low-dose (40 mg) simvastatin; 37 SAEs were reported among those taking high-dose (80 mg) simvastatin and 37 SAEs among those not taking any active treatment (ie, 0 mg of simvastatin or placebo), of which 8 were after simvastatin discontinuation of at least 8 days. Three participants’ SAE outcome (all having discontinued simvastatin or taking placebo) was death.

In total, there were 321 AEs considered related to simvastatin, reported by 72 participants (eTable 4 in Supplement 3). Of these, 214 AEs were common adverse effects of simvastatin (eTable 5 in Supplement 3); details can be found in Supplement 2. The proportion of related AEs associated with known adverse effects increased as the dose of simvastatin increased (0 mg, 93 of 171 [54.4%]; 40 mg, 48 of 66 [72.7%]; 80 mg, 68 of 80 [85.0%]). The most frequently observed common adverse effect across all groups was myalgia (0 mg, 62 [36.3%]; 40 mg, 30 [45.5%]; 80 mg, 54 [67.5%]). Of the 171 AEs reported by participants not taking active treatment, 6 occurred following simvastatin discontinuation of at least 6 days.

## Discussion

We have demonstrated that daily simvastatin, 80 mg, prescribed for 23 months, is futile for slowing motor progression in patients with PD of moderate severity, as determined by change in MDS-UPDRS part III score measured while not taking medication, a conclusion supported by the planned sensitivity analyses. We found no evidence of statistically significant differences between the simvastatin and placebo groups at 24 months in other motor or nonmotor measures, quality of life, or LEDD. Frequency and severity of AEs did not differ between the 2 groups and are similar to those previously reported.^18^

Retention of 82% of participants at the 24-month primary end point exceeded the required sample size. Our study sample was similar in terms of disease duration and baseline motor characteristics to that in the study from which the sample size calculation was derived.^13^ The calculation was based on the published MCID for the UPDRS part III score while not taking medication^12^ during study development. A subsequent publication evaluating the MCID for the MDS-UPDRS part III score while not taking medication suggested an MCID of −3.25 points for improvement and 4.63 points for worsening.^19^ Using these updated figures further supports our finding of the futility of simvastatin, although a potential cumulative benefit that might have accrued with longer-term exposure has not been explored. In our study, we observed the expected rate of progression in MDS-UPDRS part III score in the simvastatin-allocated group, with a lower-than-expected progression rate in the placebo-allocated group. The higher proportion of female participants within the placebo group, who may be more likely to have more slowly progressing tremor-dominant PD,^20^ may have contributed to this finding.

To maximize retention, particularly in view of the need for assessments while not taking medication, we allowed home visits to be conducted if required. In addition, 6 of our primary outcome assessments at the 24-month follow-up were conducted by video because of lockdown necessitated by the COVID-19 pandemic.^21^ However, sensitivity analyses demonstrate that neither visit location nor outcome assessment modality influenced our primary finding.

There were some interesting findings in our study. First, there was nonsignificant worsening of MDS-UPDRS part III score in participants taking simvastatin compared with those taking placebo. It has been suggested that there may be an inverse association between serum cholesterol level and rate of PD progression^22^ as well as cognitive and motor function,^23^ which may underlie the trend observed, as well as the difference in MDS-UPDRS part III score in favor of placebo at 12 months. However, we also found a greater increase in LEDD in the placebo group at 12 months compared with the simvastatin group, which might have affected the outcome measures at this time point. We undertook assessments in the practically defined state of not taking medication, but nevertheless, long-duration effects of dopamine replacement therapies, including levodopa, are an important potential confounder,^24^ which we will evaluate in post hoc analyses.

MDS-UPDRS part III score improved for both groups at 26 months compared with 24 months for participants having in-person assessments. This could be due to those with more severe disease withdrawing from the study or having their 26-month visits conducted remotely (with remote assessments exceeding the missing-item threshold for imputation/calculation of outcome, thus leading to a biased sample). However, this observation could relate to other factors influencing the outcome measure such as the participant’s trial experience. Understanding such potential influences could be important for future studies.

### Limitations

This study recruited participants with moderate PD, rather than an incident medication-naive population. To standardize motor assessments to account for the variable response to dopamine replacement therapy, we included the presence of wearing-off phenomenon as an inclusion criterion and primary outcome assessments were conducted in the practically defined state of not taking medication. To maximize retention, we allowed for continued titration of dopamine replacement therapies. This approach also reduced the risk of inclusion of atypical PD but potentially reduced sensitivity of the outcome measure to change.^25^ Additionally, the lack of ethnic diversity among trial participants limits the generalizability of the findings.

It could be that investigation in particular subgroups or very early PD might be more fruitful in terms of detecting a protective effect,^24^ a notion supported by epidemiological studies demonstrating a reduced risk of PD with statin use^5,6,7^ and the recently reported trial of lovastatin,^26^ although the trend in favor of treatment reported in that small, short-duration study was not statistically significant.

There are many potential mechanisms by which simvastatin might exert a protective effect^2^ but none that lends itself readily to demonstration of target engagement or biological effect within the context of a clinical trial. Simvastatin is known to be one of the more brain-penetrant statins, and our chosen dose of 80 mg once daily, as well as being the maximum licensed dose, is also the dose demonstrated to have efficacy in a phase 2 study in individuals with secondary multiple sclerosis.^18^ We saw the expected reduction in cholesterol levels in participants taking simvastatin, providing objective evidence of adherence. We are as confident as we can be that the dosing was adequate to achieve a response.

## Conclusions

In this study, we have robustly demonstrated futility of simvastatin for slowing motor progression in patients with moderate severity PD. The relationship between PD and cardiovascular risk factors, including cholesterol level, is complicated.^27^ A better understanding of the interplay between the potential protective effect of statins, the potential negative effect of low cholesterol level, the stage of disease,^28^ and relevant comorbidities might inform whether, when, and in whom statins merit further investigation as disease-modifying therapy in PD.

## Notes

Supplement 1. Trial Protocol

Supplement 2. Statistical Analysis Plan

Supplement 3. **eTable 1.** Summary Statistics of the Baseline Characteristics by Allocated Group of All Recruited Participants **eTable 2.** Results of the COVID-19 Sensitivity Analyses of the Secondary Outcomes Mixed-Effects Linear Regression Models at 24 Months **eTable 3.** Frequency and Percentage of Serious Adverse Events (SAEs) by Reported Dose of Simvastatin at the Time of the SAE **eTable 4.** Frequency and Percentage of Related Adverse Events (AEs) by Reported Dose of Simvastatin at the Time of the Related AE **eTable 5.** Frequency and Percentage of Related Adverse Events (AEs) Identified as Common Adverse Effects of Simvastatin **eAppendix.** Acknowledgement of Site Contributions

Supplement 4. PD STAT Study Group

Supplement 5. Data Sharing Statement

## References

[1] Dorsey ER, Bloem BR. The Parkinson pandemic—a call to action. JAMA Neurol. 2018;75(1):9-10. doi:10.1001/jamaneurol.2017.3299 29131880

[2] Carroll CB, Wyse RKH. Simvastatin as a potential disease-modifying therapy for patients with Parkinson’s disease: rationale for clinical trial, and current progress. J Parkinsons Dis. 2017;7(4):545-568. doi:10.3233/JPD-171203 29036837 PMC5676977

[3] Vuletic S, Riekse RG, Marcovina SM, Peskind ER, Hazzard WR, Albers JJ. Statins of different brain penetrability differentially affect CSF PLTP activity. Dement Geriatr Cogn Disord. 2006;22(5-6):392-398. doi:10.1159/000095679 16960448

[4] Sierra S, Ramos MC, Molina P, Esteo C, Vázquez JA, Burgos JS. Statins as neuroprotectants: a comparative in vitro study of lipophilicity, blood-brain-barrier penetration, lowering of brain cholesterol, and decrease of neuron cell death. J Alzheimers Dis. 2011;23(2):307-318. doi:10.3233/JAD-2010-101179 21098985

[5] Bai S, Song Y, Huang X, . Statin use and the risk of Parkinson’s disease: an updated meta-analysis. PLoS One. 2016;11(3):e0152564. doi:10.1371/journal.pone.0152564 27019096 PMC4809483

[6] Casula M, Mozzanica F, Scotti L, . Statin use and risk of new-onset diabetes: a meta-analysis of observational studies. Nutr Metab Cardiovasc Dis. 2017;27(5):396-406. doi:10.1016/j.numecd.2017.03.001 28416099

[7] Sheng Z, Jia X, Kang M. Statin use and risk of Parkinson’s disease: a meta-analysis. Behav Brain Res. 2016;309:29-34. doi:10.1016/j.bbr.2016.04.046 27131781

[8] Carroll CB, Webb D, Stevens KN, . Simvastatin as a neuroprotective treatment for Parkinson’s disease (PD STAT): protocol for a double-blind, randomised, placebo-controlled futility study. BMJ Open. 2019;9(10):e029740. doi:10.1136/bmjopen-2019-029740 31594876 PMC6797358

[9] Stacy M, Hauser R, Oertel W, . End-of-dose wearing off in Parkinson disease: a 9-question survey assessment. Clin Neuropharmacol. 2006;29(6):312-321. doi:10.1097/01.WNF.0000232277.68501.08 17095894

[10] Goetz CG, Tilley BC, Shaftman SR, ; Movement Disorder Society UPDRS Revision Task Force. Movement Disorder Society-sponsored revision of the Unified Parkinson’s Disease Rating Scale (MDS-UPDRS): scale presentation and clinimetric testing results. Mov Disord. 2008;23(15):2129-2170. doi:10.1002/mds.22340 19025984

[11] Tilley BC, Palesch YY, Kieburtz K, ; NET-PD Investigators. Optimizing the ongoing search for new treatments for Parkinson disease: using futility designs. Neurology. 2006;66(5):628-633. doi:10.1212/01.wnl.0000201251.33253.fb 16534099

[12] Shulman LM, Gruber-Baldini AL, Anderson KE, Fishman PS, Reich SG, Weiner WJ. The clinically important difference on the Unified Parkinson’s Disease Rating Scale. Arch Neurol. 2010;67(1):64-70. doi:10.1001/archneurol.2009.295 20065131

[13] Aviles-Olmos I, Dickson J, Kefalopoulou Z, . Exenatide and the treatment of patients with Parkinson’s disease. J Clin Invest. 2013;123(6):2730-2736. doi:10.1172/JCI68295 23728174 PMC3668846

[14] Stevens KN. Simvastatin as a neuroprotective treatment for Parkinson’s disease: a double-blind, randomised, placebo controlled futility study in patients of moderate severity). Accessed October 3, 2022. https://pearl.plymouth.ac.uk/bitstream/handle/10026.1/16728/PD%20stat%20SAP%20v2.2%20signed.pdf?sequence=9&isAllowed=y 10.1136/bmjopen-2019-029740 PMC6797358 31594876

[15] Keezer MR, Wolfson C, Postuma RB. Age, gender, comorbidity, and the MDS-UPDRS: results from a population-based study. Neuroepidemiology. 2016;46(3):222-227. doi:10.1159/000444021 26967747

[16] Skorvanek M, Martinez-Martin P, Kovacs N, . Differences in MDS-UPDRS scores based on Hoehn and Yahr stage and disease duration. Mov Disord Clin Pract. 2017;4(4):536-544. doi:10.1002/mdc3.12476 30363418 PMC6174385

[17] Levin B. Selection and futility designs. In: Ravina B, Cummings J, McDermott M, Poole RM, eds. Clinical Trials in Neurology: Design, Conduct, Analysis. Cambridge University Press; 2012:78-90.

[18] Chataway J, Schuerer N, Alsanousi A, . Effect of high-dose simvastatin on brain atrophy and disability in secondary progressive multiple sclerosis (MS-STAT): a randomised, placebo-controlled, phase 2 trial. Lancet. 2014;383(9936):2213-2221. doi:10.1016/S0140-6736(13)62242-4 24655729

[19] Horváth K, Aschermann Z, Ács P, . Minimal clinically important difference on the Motor Examination part of MDS-UPDRS. Parkinsonism Relat Disord. 2015;21(12):1421-1426. doi:10.1016/j.parkreldis.2015.10.006 26578041

[20] Georgiev D, Hamberg K, Hariz M, Forsgren L, Hariz GM. Gender differences in Parkinson’s disease: a clinical perspective. Acta Neurol Scand. 2017;136(6):570-584. doi:10.1111/ane.12796 28670681

[21] Committee for Human Medicinal Products. Points to consider on implications of coronavirus disease (COVID-19) on methodological aspects of ongoing clinical trials. Accessed October 1, 2022. https://www.ema.europa.eu/documents/scientific-guideline/points-consider-implications-coronavirus-disease-covid-19-methodological-aspects-ongoing-clinical_en-0.pdf

[22] Huang X, Auinger P, Eberly S, ; Parkinson Study Group DATATOP Investigators. Serum cholesterol and the progression of Parkinson’s disease: results from DATATOP. PLoS One. 2011;6(8):e22854. doi:10.1371/journal.pone.0022854 21853051 PMC3154909

[23] Sterling NW, Lichtenstein M, Lee EY, . Higher plasma LDL-cholesterol is associated with preserved executive and fine motor functions in Parkinson’s disease. Aging Dis. 2016;7(3):237-245. doi:10.14336/AD.2015.1030 27330838 PMC4898920

[24] Athauda D, Foltynie T. Challenges in detecting disease modification in Parkinson’s disease clinical trials. Parkinsonism Relat Disord. 2016;32:1-11. doi:10.1016/j.parkreldis.2016.07.019 27499048

[25] Elm JJ, Goetz CG, Ravina B, ; NET-PD Investigators. A responsive outcome for Parkinson’s disease neuroprotection futility studies. Ann Neurol. 2005;57(2):197-203. doi:10.1002/ana.20361 15668964

[26] Lin CH, Chang CH, Tai CH, . A double-blind, randomized, controlled trial of lovastatin in early-stage Parkinson’s disease. Mov Disord. 2021;36(5):1229-1237. doi:10.1002/mds.28474 33449392

[27] Potashkin J, Huang X, Becker C, Chen H, Foltynie T, Marras C. Understanding the links between cardiovascular disease and Parkinson’s disease. Mov Disord. 2020;35(1):55-74. doi:10.1002/mds.27836 31483535 PMC6981000

[28] Johnson ME, Stecher B, Labrie V, Brundin L, Brundin P. Triggers, facilitators, and aggravators: redefining Parkinson’s disease pathogenesis. Trends Neurosci. 2019;42(1):4-13. doi:10.1016/j.tins.2018.09.007 30342839 PMC6623978

