# Supplementary material for: Evaluation of Simvastatin as a Disease-Modifying Treatment for Patients With Parkinson Disease: A Randomized Clinical Trial
Source: JAMA Neurol. 2022 Oct 31;79(12):1232–41. doi: 10.1001/jamaneurol.2022.3718 (PMC9623477; doi:10.1001/jamaneurol.2022.3718)
Supplement: Supplement 1. — Trial Protocol [file jamaneurol-e223718-s001.pdf]

STUDY PROTOCOL

Simvastatin as a neuroprotective treatment for Parkinson's disease: a double-blind, randomised, placebo controlled futility study in patients of moderate severity.

Simvastatin as a neuroprotective treatment for moderate Parkinson's disease (PD STAT)

Version 4.6
22 April 2020

Chief Investigator: Dr Camille Carroll
Associate Professor and Honorary Consultant Neurologist
Peninsula Medical School
Faculty of Medicine and Dentistry
University of Plymouth
N14, ITTC 1, Plymouth Science Park
Plymouth
PL6 8BX

Study Sponsor: University Hospitals Plymouth NHS Trust
Research Office
Level 2, MSCP,
Bircham Park Offices,
1 Roscoff Rise
Derriford, Plymouth
PL6 5FP

EudraCT number: 2015-000148-40
IRAS Number: 172703
REC Reference: 15/NE/0324
ISRCTN16108482

TABLE OF CONTENTS

1 SIGNATURES 5
2 KEY CONTACT DETAILS 6
3 LIST OF ABBREVIATIONS 7
4 STUDY SUMMARY 8
5 BACKGROUND AND RATIONALE 10
6 AIMS AND OBJECTIVES 10
6.1 Aim 10
6.2 Objectives 10
7 TRIAL DESIGN 11
7.1 Primary outcome 12
7.2 Secondary outcomes (at 12,24 and 26 months) 12
8 STUDY PARTICIPANTS 12
8.1 Inclusion criteria 12
8.2 Exclusion criteria 12
9 PARTICIPANT IDENTIFICATION 13
9.1 Recruitment from clinical lists 13
9.2 Recruitment via research registers 13
9.3 Recruitment via publicity and word of mouth 13
10 SCREENING AND CONSENT 13
10.1 Telephone contact 1: Time -10 to -8 weeks approximately 13
10.2 Visit 1: Screening (Time -8 to -2 weeks approximately) 14
10.2.1 Diagnosis of Parkinson's disease 14
10.2.2 Inclusion of women of child-bearing potential 14
10.2.3 Consent 14
10.2.4 Screening procedures 14
10.2.5 Screening for type 2 diabetes 15
10.2.6 Calculation of a QRISK®2 score 15
10.2.7 Booking the baseline visit and final confirmation of eligibility 16
11 RANDOMISATION 16
12 SITE STUDY TEAMS 16
13 BASELINE (VISIT 2) 17
13.1 Baseline assessment (Time 0) 17
13.2 Provision of first medication supply 17
13.3 Participant diaries 17
14 EXPERIENCE OF TRIAL PARTICIPATION SUB-STUDY 17
14.1 Experience of trial participation survey 17
14.2 Semi-structured interviews with PD STAT participants 18
14.3 Focus groups with study participants and carers 19
14.4 Analysis 19
15 SITE STAFF EXPERIENCE OF SUPPORTING PD STAT (OPTIONAL SUB-STUDY) 19
15.1 Aim of site staff sub-study 19
15.1.1 Site staff survey 19

15.1.2 Qualitative interviews 19
15.2 Analysis 20
16 BLOOD SAMPLES FOR GENETIC ANALYSIS (OPTIONAL SUB-STUDY) 20
16.1 Aims of genetic sub-study 20
16.2 Sample collection 20
16.2.1 UCL sample 20
16.2.2 ECACC sample 21
16.3 Analyses 21
16.3.1 Genetic analysis 21
16.3.2 Pharmacogenomic analysis 21
16.3.3 Other analyses 21
16.4 Sample and data sharing 21
16.5 Confidentiality 22
16.6 Follow-up and future contact 22
16.7 Withdrawal 22
17 PARTICIPANT FOLLOW UP 23
17.1 Practically–defined OFF state 23
17.2 Telephone contact 2 (Time 2 weeks) 23
17.3 Visit 3: 1 month follow-up clinic 23
17.4 Visit 4: 6 month follow-up clinic 23
17.5 Visit 5: 12 month follow-up clinic 24
17.6 Visit 6: 18 month follow-up clinic 25
17.7 Visit 7: 24 month follow up clinic 25
17.8 Visit 8: 26 month follow up clinic 25
17.9 Telephone contacts: 2, 4, 8, 10, 14, 16, 20 and 22 months post baseline 26
17.10 Flexibility of visit schedule 26
17.11 Flexibility of follow-up visit location 27
18 TRIAL TREATMENTS 27
18.1 Active and comparator treatments 27
18.2 Packaging and labelling 27
18.3 Provision and storage of trial treatments 27
18.4 Prescription and dispensing of trial treatment 27
18.5 Drug accountability 27
18.6 Treatment schedule and supply to participants 27
18.7 Monitoring of trial treatment safety and tolerability 28
18.8 Stopping criteria for discontinuation of trial treatment 29
18.9 Dose adjustments 29
18.9.1 Dose alteration during lower dose phase 29
18.9.2 Dose alteration during higher dose phase and dose re-challenge 30
18.9.3 Temporary/permanent cessation of treatment due to contraindicated medication ... 30
19 PHARMACOVIGILANCE 30
19.1 Definitions 30
19.2 Reporting non-serious adverse events 30

19.3 Reporting serious adverse events 30
19.4 Suspected Unexpected Serious Adverse Reaction 31
20 UNBLINDING 31
21 SUBJECT WITHDRAWAL 32
21.1 Withdrawal from treatment 32
21.2 Withdrawal from follow-up 32
21.3 Premature termination of study 32
22 DATA MANAGEMENT 32
22.1 Subject numbering 32
22.2 Data collection 33
22.3 Data entry 33
22.4 Data confidentiality and security 33
22.5 Archiving 34
23 STATISTICAL CONSIDERATIONS 34
23.1 Sample size 34
23.2 Statistical analysis 35
24 DATA MONITORING AND QUALITY ASSURANCE 35
25 TRIAL OVERSIGHT 36
25.1 Trial Management Group (TMG) 36
25.2 Trial Steering Committee (TSC) 36
25.3 Data Monitoring Committee (DMC) 36
26 ETHICS AND REGULATORY APPROVALS 36
26.1 Sponsor 36
26.2 Research governance 37
27 STATEMENT OF INDEMNITY 37
28 PUBLICATION POLICY 37
29 FINANCE 37
30 REFERENCES 37
31 APPENDICES 39

Figure 1: Patient pathway 11
Figure 2 : The Patriot electromagnetic sensors (Polhemus Inc.) 45

1 SIGNATURES

Role	Name	Signature	Date
Chief Investigator	Dr Camille Carroll	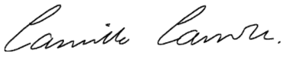	22.4.20
Statistician	Prof Siobhan Creanor	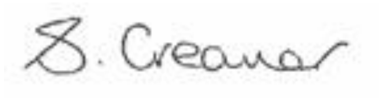	22.4.20
Sponsor's representative	Dr Chris Rollinson	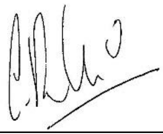	22.4.20

2 KEY CONTACT DETAILS

Chief Investigator
Dr Camille Carroll
Honorary Consultant Neurologist
Peninsula Medical School
Faculty of Medicine and Dentistry
University of Plymouth
N16, ITTC 1
Plymouth Science Park
Plymouth PL6 8BX
camille.carroll@plymouth.ac.uk

Sponsor's representative
Dr Chris Rollinson
Research Governance Manager
University Hospitals Plymouth NHS Trust
Research Office
Level 2, MSCP,
Bircham Park Offices,
1 Roscoff Rise
Derriford, Plymouth
PL6 5FP
crollinson@nhs.net

Statistician
Prof Siobhan Creanor
Associate Professor in Medical Statistics & Clinical Trials
Peninsula Medical School
Faculty of Medicine and Dentistry
University of Plymouth
N15, ITTC Building 1, Plymouth Science Park
Plymouth PL6 8BX
siobhan.creanor@plymouth.ac.uk

Trial Manager
Dr Alison Jeffery
Peninsula Clinical Trials Unit
Peninsula Medical School
Faculty of Medicine and Dentistry
University of Plymouth
N16, ITTC Building 1
Plymouth Science Park
Plymouth PL6 8BX
alison.jeffery@plymouth.acuk

Tel: 01752 439830

Assistant Trial Manager
Miss Rebecca Chapman
Peninsula Clinical Trials Unit
Peninsula Medical School
Faculty of Medicine and Dentistry
University of Plymouth
N16, ITTC Building 1
Plymouth Science Park
Plymouth PL6 8BX
rebecca.chapman@plymouth.ac.uk

Tel: 01752 439830

Study medication provider
Ms Nicky Heath
Specialist Pharmacy Technician
Royal Free London NHS Foundation Trust
Royal Free Hospital
Pharmacy Department
Pond Street
London NW3 2QG
nicky.heath@nhs.net

Tel: 020 7317 7531 ext 36890
Fax: 020 7830 2621

Study medication distributor
Mrs Maggie Kalita/Mr Mike Marner
Pharmacy Department
Derriford Hospital
Derriford Road
Plymouth
PL6 8DH
mike.marner@nhs.net
maggie.kalita@nhs.net

Tel: 01752 432655 or 432371
Fax: 01752 763423

3 LIST OF ABBREVIATIONS

ACE-III	Addenbrooke's Cognitive Examination-III
ADL	Activities of Daily Living
AE	Adverse Event
ALT	Alanine transaminase
AST	Aspartate transaminase
CI	Chief Investigator
CK	Creatine kinase
CRF	Case Report Form
CT	Computed Tomography
CTA	Clinical Trials Authorisation
CTIMP	Clinical Trial of an Investigational Medicinal Product
CTU	Clinical Trials Unit
CVD	Cerebrovascular Disease
DMC	Data Monitoring Committee
ECACC	European Collection of Cell Cultures
EDTA	Ethylenediaminetetraacetic acid
eGFR	estimated Glomerular Filtration Rate
EMS	Electromagnetic Sensor Measurement
EQ-5D-5L	EuroQoL 5D-5L health status questionnaire
HDL	High density lipoprotein
LED	Levodopa-equivalent dose
ICH GCP	International Conference on Harmonisation of Good Clinical Practice
MADRS	Montgomery and Asberg Depression Rating Scale
MDS-UPDRS	Movement Disorder Society Unified Parkinson's disease Rating Scale
MHRA	Medicines and Healthcare products Regulatory Agency
MoCA	Montreal Cognitive Assessment
MRI	Magnetic Resonance Imaging
NMSS	Non-motor symptoms scale
PD	Parkinson's disease
PDQ-39	Parkinson's disease Questionnaire
UHPNT	University Hospitals Plymouth NHS Trust
PI	Principal Investigator
PIS	Participant Information Sheet
QALY	Quality Adjusted Life Year
REC	Research Ethics Committee
SAE	Serious Adverse Event
SmPC	Summary of Product Characteristics
SOP	Standard Operating Procedure
SUSAR	Suspected Unexpected Serious Adverse Reaction
TMG	Trial Management Group
TSC	Trial Steering Committee
U&Es	Urea and electrolytes
ULN	Upper Limit of Normal

4 STUDY SUMMARY

Title	Simvastatin as a neuroprotective treatment for Parkinson's disease
Study location	NHS sites across UK (lead site Derriford Hospital, Plymouth)
Study aim	To determine whether the cholesterol-lowering drug, simvastatin, has potential as a neuroprotective therapy in Parkinson's disease (PD).
Study design	Randomised, double blind, placebo-controlled trial.
Study population	
Inclusion criteria	• Diagnosis of idiopathic PD<br>• Modified Hoehn and Yahr stage (≤3.0) in the ON medication state<br>• Age 40-90<br>• On dopaminergic treatment with wearing-off phenomenon<br>• Able to comply with study protocol and willing to attend study visits
Exclusion criteria	• Diagnosis or suspicion of other cause for parkinsonism<br>• Known abnormality on CT or MRI brain imaging considered to be causing symptoms or signs of neurological dysfunction, or considered likely to compromise compliance with study protocol<br>• Concurrent dementia defined by MoCA score <21<br>• Concurrent severe depression defined by MADRS score >31<br>• Prior intracerebral surgical intervention for PD including deep brain stimulation, lesional surgery, growth factor administration, gene therapy or cell transplantation<br>• Already actively participating in a research study that might conflict with this trial<br>• Prior or current use of statins as a lipid lowering therapy<br>• Intolerance to statins<br>• Untreated hypothyroidism<br>• End stage renal disease (creatinine clearance <30 mL/min) or history of severe cardiac disease (angina, myocardial infarction or cardiac surgery in preceding two years)<br>• eGFR <30 mL/min<br>• History of alcoholism or liver impairment<br>• Creatine kinase (CK) >1.1 x upper limit of normal (ULN)<br>• Serum transaminases (AST or ALT) >1.1 x ULN<br>• Females who are pregnant or breast feeding or of child-bearing potential and unwilling to use appropriate contraception methods whilst on trial treatment<br>• Currently taking any medication contraindicated with simvastatin use<br>• Any requirement for statin use<br>• Regular participation in endurance or high-impact sports<br>• Unable to abstain from consumption of grapefruit-based products
Sample size	198 (with replacement for those not tolerating the 40mg one month phase)

Investigational Medicinal Product	
Formulation	Simvastatin, 40mg capsules or matched placebo
Route	Oral administration
Dose regimen	Lower dose phase: 40 mg daily for one month;<br>Higher dose maintenance phase: 80 mg daily for 23 months
**Summary of outcome measures**	
Primary	Change in MDS-UPDRS part III (OFF) score over 24 months.
Secondary	MDS-UPDRS total score in the practically-defined ON state; MDS-UPDRS part II (ON); Timed motor tests – finger tapping and timed walk test (10MWT), electromagnetic sensor (EMS) assessment (at selected sites); Montgomery and Asberg Depression Rating Scale (MADRS); The Addenbrooke's Cognitive Examination-III (ACE); Non-Motor Symptoms Scale (NMSS); PDQ-39; Changes in PD medication; Cholesterol levels; King's PD pain scale; EQ-5D-5L; Safety and tolerability of trial medication.
**Study schedule**	
Follow-up	Out-patient follow-up at 1, 6, 12, 18, 24 and 26 months.
Study duration	Approximately 27 months for each patient (including screening)
Study timelines	Set-up 14 months. Recruitment 25 months. Treatment and follow-up 24 months. Washout 2 months. Data cleaning, analysis, reporting 4 months. Total duration 69 months.
End of trial	Completion of last follow-up visit of last participant.

5 BACKGROUND AND RATIONALE

Parkinson's disease (PD) is a progressive neurodegenerative condition affecting more than 127,000 people in the UK with a further 10,000 individuals being diagnosed with the condition each year. No drug has been shown to slow or reverse the neurodegenerative process of PD. All currently licensed therapies act as symptom-relieving agents but have a limited lifespan of effectiveness because of continued neuronal loss. The purpose of this study is to determine whether simvastatin, a widely used cholesterol-lowering drug (statin) with an excellent safety profile (Naci et al., 2013; Lv et al., 2014), has potential to reduce the rate of neurodegenerative decline in patients with PD.

Epidemiological data support a possible neuroprotective role for statins in PD, with statin use being associated with lower PD incidence (Friedman et al., 2013; Undela et al., 2013). Simvastatin has been shown in various toxin and genetic cell culture and rodent PD models to influence several pathways thought to be of relevance in PD etiopathogenesis, including inflammation and microglial activation, oxidative stress and α-synuclein aggregation (reviewed by Roy and Pahan, 2011). A beneficial effect of simvastatin on dopamine neuron survival and motor function has been observed in acute (Ghosh et al., 2009) and chronic (Roy and Pahan, 2011) 1-methyl-4-phenyl-1,2,3,6-tetrahydropyridine mouse models. Additionally, statins may have symptomatic effects on dyskinesia and depression in PD (Roy and Pahan, 2011). Interestingly, simvastatin has recently been shown to reduce the rate of brain atrophy in secondary progressive multiple sclerosis (Chataway et al., 2014); it is likely that the mechanisms underlying neuronal death are similar in these and other neurodegenerative diseases.

Clinical trials of potential neuroprotective agents in PD are difficult to design, given the variability in disease phenotype and rate of progression, as well as the potential confounding factor of a symptomatic response. In addition there is no reliable biomarker for disease progression (Lang et al., 2013). This study uses a futility design which allows a relatively short study duration and small sample size (Schwid et al., 2006). The study is part of the Linked Clinical Trials initiative coordinated by The Cure Parkinson's Trust (Brundin et al., 2013).

6 AIMS AND OBJECTIVES

6.1 Aim

The aim of the study is to determine whether the cholesterol-lowering drug, simvastatin, has potential as a neuroprotective therapy in PD.

6.2 Objectives

Primary:

- To determine whether simvastatin is clearly ineffective (futile) in preventing the clinical decline of PD as measured by the Movement Disorder Society Unified Parkinson's Disease Rating Scale (MDS-UPDRS) motor score.

Secondary:

- To confirm the safety and tolerability of simvastatin in patients with PD.
- To distinguish symptomatic effects of simvastatin from disease modifying effects.
- To evaluate the impact of simvastatin on activities of daily living (ADL), timed motor tests, cognitive ability, mood, behaviour, non-motor symptoms (NMSS) and quality of life among patients with moderate PD using standard validated tools of assessment.

7 TRIAL DESIGN

This is a double blind, randomised, placebo-controlled, multi-centre parallel group trial in patients with PD of moderate severity. One hundred and ninety eight patients will be randomised in a 1:1 ratio to receive either oral simvastatin or matched placebo for 24 months. A one month low dose phase of 40mg daily will be followed by a 23 month high dose phase of 80mg daily and a final two month phase off trial medication. Participants will be followed up as out-patients at 1 month, 6, 12, 18, 24 and 26 months (Fig.1).

Figure 1: Patient pathway

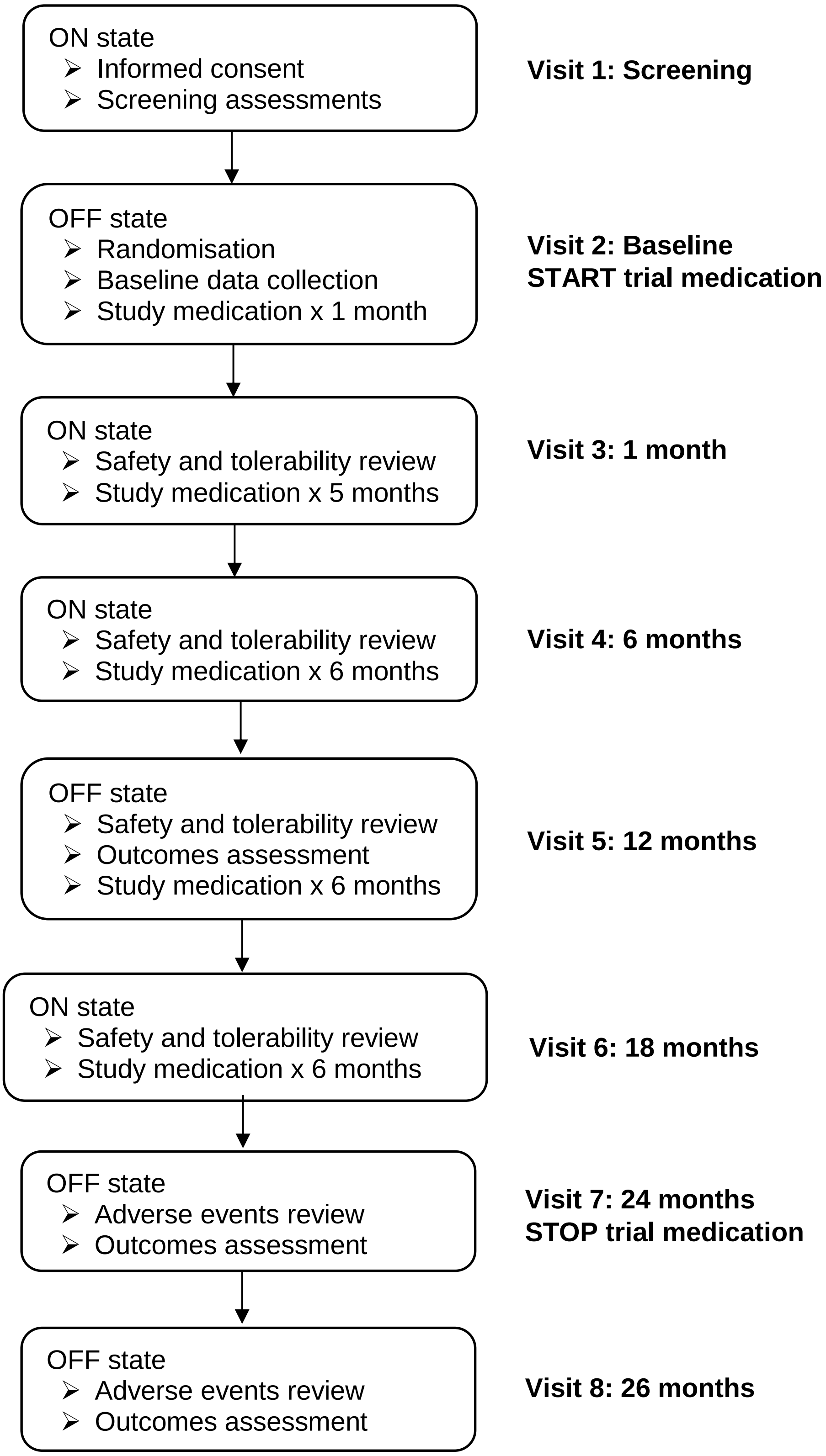

7.1 Primary outcome

The primary outcome is change in MDS-UPDRS part III motor subscale score in the OFF state over 24 months.

7.2 Secondary outcomes (at 12,24 and 26 months)

i) MDS-UPDRS total score in the practically defined ON state
ii) MDS-UPDRS part II subscale score in the practically defined ON state
iii) Timed motor tests – finger tapping and timed walk test (10MWT) in the OFF state, electromagnetic sensor (EMS) assessment in the OFF and ON state
iv) Montgomery and Asberg Depression Rating Scale (MADRS)
v) The Addenbrooke's Cognitive Assessment-III (ACE-III)
vi) Non-Motor Symptom assessment scale (NMSS)
vii) Parkinson's disease Questionnaire (PDQ-39)
viii) Changes in PD medication as measured by levodopa-equivalent dose (LED)
ix) Cholesterol levels (total, HDL, total/HDL ratio)
x) King's PD pain scale (KPPS)
xi) EuroQoL 5D-5L health status questionnaire (EQ-5D-5L)
xii) Safety and tolerability of trial medication by adverse events (AEs) review.
xiii) Incidence of diabetes mellitus at 24 months, using a glycated haemoglobin (HbA1c) level of 6.5% (48mmol/mol) as diagnostic of diabetes mellitus (WHO 2011)

Additional detail about the study outcome measures is given in Appendix 1.

8 STUDY PARTICIPANTS

8.1 Inclusion criteria

Potential participants must satisfy the following criteria to be enrolled in the study:

- Diagnosis of idiopathic PD
- Modified Hoehn and Yahr stage ≤ 3.0 in the ON medication state
- Age 40-90 years
- On dopaminergic treatment with wearing-off phenomenon
- Able to comply with study protocol and willing to attend necessary study visits

8.2 Exclusion criteria

Potential participants meeting any of the following criteria will be excluded from study participation:

- Diagnosis or suspicion of other cause for parkinsonism
- Known abnormality on CT or MRI brain imaging considered to be causing symptoms or signs of neurological dysfunction, or considered likely to compromise compliance with study protocol
- Concurrent dementia defined by MoCA score <21
- Concurrent severe depression defined by MADRS score >31
- Prior intracerebral surgical intervention for PD including deep brain stimulation, lesional surgery, growth factor administration, gene therapy or cell transplantation
- Already actively participating in a research study that might conflict with this trial
- Prior or current use of statins as a lipid lowering therapy
- Intolerance to statins
- Untreated hypothyroidism
- End stage renal disease (creatinine clearance <30 mL/min) or history of severe cardiac disease (angina, myocardial infarction or cardiac surgery in preceding two years)

- eGFR <30 mL/min
- History of alcoholism or liver impairment
- Creatine kinase (CK) >1.1 x upper limit of normal (ULN)
- Aspartate transaminase (AST) or alanine transaminase (ALT) >1.1 x ULN
- Females who are pregnant or breast feeding or of child-bearing potential and unwilling to use appropriate contraception methods whilst on trial treatment
- Currently taking any medication contraindicated with simvastatin use (Appendix 2)
- Any requirement for statin use
- Regular participation in endurance or high-impact sports
- Unable to abstain from consumption of grapefruit-based products

9 PARTICIPANT IDENTIFICATION

Recruitment will be supported by the Local Comprehensive Research Networks (LCRNs). The precise timing and method of approaching patients for the study will depend on individual patient circumstances and site logistics and will be at the discretion of the research team at each site.

9.1 Recruitment from clinical lists

Patients identified as potentially suitable for this study may be contacted by standard letter from their PD clinician in order for the patient to be put in touch with the local research team. The written Participant Information Sheet should be included with the approach letter and the letter will advise that a local research nurse will telephone the patient to ascertain further interest and eligibility for the study. Alternatively, the patient may prefer to telephone the local research team directly in order to register further interest in the study.

In addition, potentially suitable patients may be approached directly by their clinician, specialist nurse or research nurse in the out-patient setting. A brief verbal outline of the study can be given at this point and the PIS provided to those who express an interest. Arrangements should be made for a local research nurse to telephone the patient after a suitable period to ascertain further interest and eligibility for the study. Alternatively, the patient may telephone the local research team directly in order to register further interest in the study. In all cases, the research nurse will make contact with the patient to ascertain interest, provide further information and assess potential eligibility for the study as appropriate.

9.2 Recruitment via research registers

Patients may also be recruited to the study from research registers (e.g. the Pro-DeNDRoN register, a register of PD patients in the South West with an interest in research) or other similar databases. In this case potentially eligible patients will be contacted and provided with the PIS according to local protocol, to ascertain interest in the study.

9.3 Recruitment via publicity and word of mouth

Patients may learn about the study from patient support group publications or meetings (e.g. Cure Parkinson's Trust), or internet sources (e.g. Fox Trial Finder or www.ParkinsonsMovement.com). In such cases, interested patients will be signposted to local study sites for further information.

10 SCREENING AND CONSENT

A trial schedule is provided in Appendix 3.

10.1 Telephone contact 1: Time -10 to -8 weeks approximately

Patients who have registered an interest in the study will undergo telephone screening by a local research nurse to check potential eligibility, including a brief review of relevant medical history.

10.2 Visit 1: Screening (Time -8 to -2 weeks approximately)

Patients interested in and potentially eligible for the study will be invited to a local screening clinic appointment. At this visit the research team will answer any further questions, check the patient's eligibility for the study as far as possible and, if the patient wishes to participate in the study, obtain written consent (see 10.2.3).

10.2.1 Diagnosis of Parkinson's disease

A clinical diagnosis of PD is based on the opinion of the Principal Investigator (PI) after review of the participant's clinical history, examination findings and response to PD medication. The Queen Square brain bank criteria MAY be used to help assist in the diagnosis although this need not be a formal inclusion criteria.

10.2.2 Inclusion of women of child-bearing potential

Simvastatin is contraindicated during pregnancy, thus female participants who are pre-menopausal and not permanently sterilised, or whose male partner has not undergone vasectomy (with zero sperm count), will be required to use appropriate methods of contraception whilst taking trial medication. Acceptable forms of effective contraception include i) the established use of oral, injected or implanted hormonal contraceptive methods; ii) in situ intrauterine device (IUCD) or intrauterine system (IUS); iii) barrier methods of contraception combined with a spermicidal agent or iv) true abstinence, if this is the preferred or usual lifestyle of the participant.

10.2.3 Consent

If the patient appears eligible and is willing to participate, written consent will be obtained and the participant will be allocated a unique study number from a pre-supplied study screening log. This number will be used on all study-related documentation throughout the trial. The written informed consent process will be undertaken by the PI or by an appropriately trained member of the research team as delegated by the PI, depending on local arrangements. All staff undertaking the consent procedure for this study must be authorised by the PI on the site study delegation log. A record of the patient's consent to participate should be documented in the hospital notes along with a filed copy of the completed consent form and participant information sheet. A copy of the completed consent form should also be emailed/faxed to the CTU for central monitoring purposes.

10.2.4 Screening procedures

The PI (or authorised delegate) will complete the following screening assessments/procedures with the participant:

- Demographic information and medical history
- Concomitant medication and 'wearing-off' questionnaire
- Provide "Wearing off guide for patients" (for the participant to take home)
- Physical examination (including assessment of modified Hoehn & Yahr stage)
- MoCA
- MADRS
- Blood samples (local laboratory) for:
    - creatine kinase (CK)
    - aspartate transaminase (AST) or alanine transaminase (ALT)
    - estimated glomerular filtration rate (eGFR)
    - cholesterol (HDL, total)
    - urea and electrolytes (sodium, potassium, creatinine)
    - thyroid stimulating hormone (TSH)
    - glycated haemoglobin (HbA1c)

10.2.5 Screening for type 2 diabetes

There is some evidence that long-term use of high doses of simvastatin may be associated with increased risk of developing insulin resistance and type 2 diabetes mellitus, although in a recent analysis there was no reported evidence of a significant association at two years in patients taking a prescribed statin (Cederberg et al., 2015). To monitor this, patients will be screened for type 2 diabetes mellitus at screening using a glycated haemoglobin (HbA1c) level of 6.5% (48mmol/mol) as diagnostic of diabetes mellitus (WHO 2011), repeated at 24 months. A 'positive' diagnosis of diabetes according to this criterion will be applied to the calculation of the QRISK®2 screening score (section 10.2.6) even if patients do not have a current known diagnosis of diabetes mellitus.

Patients whose HbA1c is ≥6.5% (48mmol/mol) but currently do not have a diabetes diagnosis should be informed of the result by the local site team (usually by telephone, followed up in writing) and asked to discuss the implications with their GP before proceeding further with the study. The decision about whether to include the patient in the study will depend on the outcome of the GP-patient consultation, including whether the patient is prescribed statin therapy. If patients do not discuss the HbA1C result with the GP, they will be considered ineligible for the study, based on the potential requirement for statin use. Local site teams will be required to keep in touch with the patient during this process in order to ascertain eligibility for the study. For all participants eventually recruited to the study, the patient's GP will be advised in writing of the screening HbA1c result by standard letter.

10.2.6 **Calculation of a QRISK®2 score**

NICE guidelines (NICE, 2014) recommend that people with an estimated 10‑year risk of cardiovascular disease (CVD) of 10% or more should be prioritised for a full formal risk assessment for consideration of statin therapy. QRISK®2 is a commonly used CVD risk calculator which will be used in this study to check whether there may be an underlying requirement for statin therapy (exclusion criteria, section 8.2).

A QRISK®2 score will be calculated for all patients at the screening stage, as explained in the participant information sheet. Computation of the score itself will be undertaken by the PenCTU on receipt of the completed screening CRF. The maximum age that can be entered into the QRISK®2 calculator is 84, hence this age will be entered into the risk calculator for all potential participants aged ≥85 years. The score will be reported back to the relevant site staff who should pass the result to the patient (usually by telephone, followed up in writing).

If the QRISK®2 score is <10%, the patient may enter the study, assuming all other eligibility criteria are satisfied. The GP will be informed of the patient's entry into the study, including the QRISK®2 score, by standard GP information letter.

If the QRISK®2 score is ⩾10%, the local site team should advise the patient to discuss this with the GP, though the patient may elect not to do so.

- If the patient **does** discuss the score with the GP, the decision about whether to include the patient in the study will depend on the outcome of the GP-patient consultation and specifically whether the patient is prescribed statin therapy. Site staff should liaise with the patient during this period to check progress on any consultation and to ascertain whether or not the patient can be entered into the study. If the patient subsequently enters the study, the local site staff will confirm this to the GP by standard letter.

- If the patient chooses **not** to discuss a ⩾10% score with the GP but is otherwise eligible and wants to join the study, he/she can be included in the study. Local site staff will confirm study enrolment, the QRISK®2 score (and advice to discuss) by standard letter to the GP.

10.2.7 Booking the baseline visit and final confirmation of eligibility

Assuming that the patient fulfils the immediate eligibility criteria, arrangements will be made for the patient to attend a baseline appointment approximately two to eight weeks after the screening visit. This interval enables review of the patient's blood results to confirm final eligibility and allows the patient to visit the GP to discuss QRISK®2 or HbA1c results as necessary. If more than eight weeks have elapsed since the screening visit, the screening visit and screening assessments should be repeated before proceeding to the baseline visit.

Patients who consent to the study but who are subsequently found not to meet the study eligibility criteria will be contacted by the research nurse or other member of the research team to inform them that they are not eligible to join the study. If any concerns about the patient's health or well-being are identified from out-of-range blood results or abnormal assessment scores, a member of the research team will inform the patient's GP.

11 RANDOMISATION

Following written consent, randomisation will take place at some point between the screening and baseline visits, or at the baseline visit itself (depending on local arrangements for obtaining trial medication from pharmacy). Randomisation will be achieved by means of a 24-hour web-based system created by the Peninsula Clinical Trials Unit (CTU) in conjunction with a statistician independent from the trial team. Participants will be allocated to receive simvastatin or matched placebo in a 1:1 ratio, using random permuted blocks with stratification by site and modified Hoehn & Yahr stage (≤2.0, or 2.5- 3) in the ON medication state.

Once final eligibility of the patient has been confirmed, the PI (or authorised delegate) will access the password-protected randomisation website in order to allocate the participant to one of the two treatment groups. The participant's allocation will not be displayed or be otherwise accessible to the person undertaking the randomisation process. Completion of the randomisation process will generate a screen displaying the participant's study number, initials and allocated bottle number for the baseline visit. This should be printed and provided to the relevant hospital pharmacy with a study-specific prescription so that study medication can be dispensed for the baseline visit (see section 13.2).

The CTU programming team will run checks before and during the trial to verify the integrity of the randomisation system. Access to participants' treatment allocations will be restricted to the CTU programming team except in the event of a potential SUSAR (see section 17.4).

12 SITE STUDY TEAMS

At least two (and probably three) staff members are required at each site to conduct this study. The PI, supported by an appropriate member of the team (usually a research nurse or PD specialist nurse) will monitor participant well-being, record adverse events, titrate and prescribe study medication. Since the 'treating' nurse or doctor will review all blood results and be aware of any reported side effects, a separate member of the research team (usually a research nurse) will act as a blinded assessor, undertaking the MDS-UPDRS and other outcome measures after appropriate training. Ideally the blinded assessor will undertake the MDS-UPDRS Part III and the timed motor tests with the participant in the OFF state, followed by the complete MDS-UPDRS, NMSS and MADRS once the participant is in the ON state. The treating nurse should usually complete the King's PD pain scale and the ACE-III. If necessary for practical or other reasons, the treating nurse may also undertake the MADRS and the NMSS. Efforts should be made to ensure that the same outcome assessor is present at all visits but sites should identify back-up personnel to cover staff absences and avoid cross-over of 'assessing' and 'treating' team members.

13 BASELINE (VISIT 2)

13.1 Baseline assessment (Time 0)

Patients who have formally satisfied all eligibility criteria will attend a baseline visit. Participants will be asked to omit relevant PD medication so that they attend on the day of assessment in the ‘practically-defined OFF’ state (see 16.1). The PI or authorised delegate will record any reported serious adverse events and changes to concomitant medications, including PD medication, enabling subsequent calculation of the levodopa–equivalent dose (LED) by the CTU.

The assessor will complete the following procedures with the participant:

- MDS-UPDRS part III (motor examination)
- Timed motor tests (finger tapping and timed walk test)

Participants will be invited to take their routine PD medication following the motor assessments. The MDS-UPDRS (parts 1A, III, IV) and the NMSS should then be completed by the assessor with the participant in the practically-defined ON state. The ACE-III and the King’s PD pain scale should preferably be completed by the PI or authorised treating nurse. Following a brief explanation of each measure, participants will then be asked to complete the MDS-UPDRS (parts 1B and II), the PDQ-39 and the EQ-5D-5L.

13.2 Provision of first medication supply

If randomisation has not taken place prior to the baseline visit, participants will be randomised at this visit (as detailed in section 11). The PI or authorised delegate will complete and sign a study-specific prescription form for presentation to pharmacy with the baseline bottle allocation print-out from the randomisation process. All participants will be prescribed 40mg daily (simvastatin or placebo equivalent) for the initial one month low dose phase. It is expected that participants will start study treatment on the day of the baseline visit.

13.3 Participant diaries

All participants will be provided with a study-specific diary by their local research team, in which to record any alterations in the dose of trial medication or concomitant medications taken, and any AEs. The study diary is intended to serve as an aide-memoire so participants will be asked to bring their completed diaries to each study visit to aid Case Report Form (CRF) completion. Participants should be advised to contact the study team promptly should they develop unexplained muscle pain, tenderness or weakness rather than waiting for the next study visit or scheduled telephone call (see section 16.9).

14 EXPERIENCE OF TRIAL PARTICIPATION SUB-STUDY

The aim of this sub-study is to develop an understanding of the barriers and facilitators to participating in clinical trials for people living with PD. The design of this sub-study may be further modified and specified, based on the funding available and further involvement of people with lived experience of PD.

14.1 Experience of trial participation survey

All participants recruited and randomised to the study will be invited to complete an ‘experience of trial participation’ survey on three occasions during their involvement in the study. The survey will ask participants to rate their experiences of taking part in the trial, on a Likert-type scale. Free form space will be given at the end of each survey for those who wish to make further comments about any particular experiences. The surveys will be sent directly to participants by PenCTU at baseline (following randomisation) and just prior to the 12 month and 26 month visits, along with a freepost

envelope. Data from the survey will be used to inform the topic guides for the semi-structured interviews and focus groups (see below).

As part of this quantitative survey process, participants will also be asked about their willingness to be approached to take part in a qualitative study. Participants expressing an interest in the qualitative study will be sent an information sheet to give them a greater understanding of what will be involved. Participants will be selected by the PD STAT research team, in conjunction with the PenCTU, from those who express willingness to participate during correspondence with the researcher.

14.2 Semi-structured interviews with PD STAT participants

The qualitative study will be restricted to PD STAT study sites in the South West of England for practical reasons. Up to 10 participants recruited to the PD STAT study and who express an interest in the qualitative study will be purposively selected to take part in two separate face-to-face, semi-structured interviews with a researcher. The first interview will take place after the participant has attended the one month PD STAT study visit. Should interview data reach saturation and no new interviews need to be conducted then those participants who have expressed an interest in taking part but are no longer needed for the research will be sent a thank you letter and will be sent details of the results at the end of the qualitative study. Maximum variation (a mixture of ages, gender and disease progression) will be used to decide the final number of participants.

Interviews will be conducted in a place in which the participant feels comfortable and will take up to one hour. Separate written informed consent will be obtained from participants involved in these qualitative interviews. The interviews will explore a variety of issues including participants' motivation to take part in the PD STAT study, factors that made it easier (or more difficult) for them to take part, their feelings and whether their participation in this study has influenced their willingness to take part in future trials. Draft interview guides have been created but may be amended in light of information gained through the pre-interview surveys. All names will be changed to protect the participant's confidentiality.

At the end of the first interviews, those participants who have been interviewed will be asked whether they would be willing to part in i) a focus group in approximately six months' time (see below) and ii) a further individual interview at the end of the study. The end of the study interviews will be conducted in the same way as the initial interviews. Interviews and focus group discussions will be digitally audio-recorded and transcribed verbatim. If participants choose to leave the qualitative study, new participants will be included to maintain the depth and breadth of the data.

Up to 10 additional semi-structured interviews will be carried out with people who volunteer to take part in the PD STAT study, but who do not fulfil the entry criteria. These interviews will focus on how the potential participant feels about this experience and the likely impact of this experience on their desire and willingness to participate in future trials. These interviews are likely to take 15-30 minutes and will be digitally audio-recorded and transcribed verbatim. These transcripts will also undergo a process of inductive thematic analysis; the results will be considered both separately and combined with the interviews with the trial participants.

Up to 10 semi-structured interviews will also be carried out with participants who discontinue trial treatment or withdraw from the study during follow up. These interviews will be recorded, transcribed and analysed to gain an understanding of what are the range of, and most likely factors in influencing dropping out prematurely. The purpose of identifying these factors is not to produce an exhaustive list, but to consider whether, in any future trial, they could be addressed in order to maximise recruitment and retention.

14.3 Focus groups with study participants and carers

One focus group consisting of eight to ten PD STAT participants will be held once participants have attended the six month clinic visit. The focus group will ideally be made up of participants who have taken part in the initial qualitative interview and will last for up to one and a half hours. If participants have withdrawn from the qualitative sub-study, new participants will be purposively selected from those originally expressing an interest in the qualitative study and will be invited to take part in the focus group and/or end of study interviews.

A second focus group consisting of eight to ten carers of PD STAT participants will be held at the end of the study (around month 26), lasting up to one and a half hours. Study site staff will be asked to give a carers' focus group information sheet to PD STAT trial participants attending for their 24 month visit, to pass to their carer. Carers who express an interest in being involved in the focus groups will be recruited to the project by the researcher in response to the return of a reply slip. The focus groups will discuss the experience of caring for people living with PD and the input required to enable a person with PD to take part in a clinical trial

Focus groups will be held in as convenient a location as possible for participants and carers and appropriate written informed consent will be obtained from those attending. The focus group discussions will be digitally audio-recorded and transcribed verbatim.

14.4 Analysis

All recordings will be transcribed and analysed thematically (Saldano, 2013). Transcripts will be read and re-read and initial ideas (codes) recorded across each of the transcripts. A second qualitative researcher will also code a selection of the transcripts and the codes compared to ensure credibility of the process. Final themes developed from a synthesis of the codes will be discussed with the main PD STAT research team.

15 SITE STAFF EXPERIENCE OF SUPPORTING PD STAT (OPTIONAL SUB-STUDY)

15.1 Aim of site staff sub-study

The aim of this qualitative sub-study is inform future research in Parkinson's studies. The sub-study will explore the experiences of site staff who have supported the PD STAT study and its participants, with particular interest in the challenges associated with participant retention. This sub-study will be conducted towards the end of the trial and will comprise two methods:

15.1.1 Site staff survey

The survey will be posted or emailed to staff members at all participating sites, to include research nurses, research coordinators, assessors and principal investigators,

15.1.2 Qualitative interviews

A qualitative researcher will conduct telephone interviews with staff from around ten selected sites, to include sites with good and poor retention. PenCTU staff will contact staff at these sites by email, attaching the relevant participant information sheet, asking if they would like to participate in an optional telephone interview. PenCTU will give the qualitative researcher a list of those who agree to participate and give consent to be contacted. The interviews will explore a range of issues including the challenges to the site staff supporting participants in the trial and thoughts about improving the trial experience for participants, carers and the team. These interviews are likely to take 30-60 minutes and will be digitally audio-recorded and transcribed verbatim.

15.2 Analysis

All recordings will be transcribed and analysed thematically as described in section 14.4 above. Site staff participants will be identified by an ID number only.

Audio files and transcripts produced will be stored securely on a University of Plymouth SharePoint site. Identifiable information will be omitted from the interview transcripts.

16 BLOOD SAMPLES FOR GENETIC ANALYSIS (OPTIONAL SUB-STUDY)

PD STAT study participants will be provided with a separate Participant Information Sheet (PIS) for the genetics sub-study prior to the 12 month clinic visit. This will usually be posted directly to participants from the CTU at the same time as the 12 month feedback questionnaire (section 13.4). At the 12 month clinic visit, participants will be given an opportunity to discuss the genetics sub-study with the local study team and to have any questions answered. Those agreeing to participate in the sub-study will be asked to provide separate written informed consent. Two 10mL blood samples (approximately 2 tablespoons) will then be taken, usually at the 12 month clinic visit (although samples can be taken at a subsequent clinic visit instead if the participant wishes); one 10mL blood sample in an EDTA sample tube for the extraction of inherited material by University College London (UCL) and one 10mL blood sample in an acid citrate dextrose (ACD) sample tube for the European Collection of Cell Cultures (ECACC), a UK based repository.

16.1 Aims of genetic sub-study

The aim of this sampling is to try to identify genetic markers that may be associated with PD disease course, severity or variation in treatment responsiveness. The primary aim of this resource and of future work will be to enable targeting of the best treatments to specific patient groups.

Secondary outcomes will include:

- Identification of research-engaged patients carrying specific genetic variants for future studies
- Correlation of high quality clinical data with genotype
- Correlation of biochemical trial marker data (e.g. cholesterol) with genotype
- Investigation of pharmacokinetic variables linked to absorption, distribution, metabolism and excretion of therapeutic agents (ADME).

16.2 Sample collection

Instructions for collection, packing and dispatching samples are included in the investigator site file.

16.2.1 UCL sample

One sample will be collected in an EDTA tube, packaged and sent by post at room temperature from individual study sites to UCL Neurogenetics Department, 6th Floor, Institute of Neurology, Queen Square House, Queen Square, London WC1N 3BG, to be stored with other samples in a biobank within the Institute of Neurology. A copy of the signed consent form for the genetics sub-study will be sent to the PenCTU for review, and will be forwarded to the team at UCL with participant contact details if the participant has consented to being contacted in the future. The inherited material (DNA and genes) will be extracted from the whole blood sample in accordance with the analytical plan agreed by the PD STAT genetic sub-study investigators (Dr Camille Carroll and Professor Huw Morris) and their teams. The inherited material will be stored in the Cure Parkinson's Trust DNA bank, a subset of the Clinical Neurological Disease Biobank and Neurogenetics Research Study (CANDAS) DNA bank, and used for genetic investigations into PD. The Neurogenetics laboratory will process, store and dispose of blood samples in accordance with all applicable legal and

regulatory requirements, including the Human Tissue Act, 2004 and any amendments thereto. Professor Huw Morris will be the custodian of the samples and any accompanying data.

16.2.2 ECACC sample

The second sample will be packaged and sent directly by post from individual study sites to ECACC, Genetic Support Services, Culture Collections, Public Health England, Salisbury, Wiltshire SP4 0JG, for preparation and storage of peripheral blood lymphocytes (PBLs) and potential future preparation of immortalised cell lines. This whole blood sample must be kept at room temperature in the ACD tube and must not be frozen or refrigerated.

The advantages of storing cell lines are the provision of an ongoing source of DNA for future studies and the facilitation of large-scale collaborative studies. ECACC will process, store and dispose of blood samples in accordance with all applicable legal and regulatory requirements, including the Human Tissue Act, 2004 and any amendments thereto. Cell lines/lymphocytes for cell line preparation will be stored at ECACC encoded by the participant’s unique PD STAT study number, provided by the local study site. To enable the cell line to be of optimal use to the research community, ECACC will also be provided with strictly limited participant details - specifically gender, ethnicity, year of birth and PD diagnosis. No further personal identifying details will be released to ECACC. Participants can request withdrawal of their samples from ECACC at any point.

16.3 Analyses

16.3.1 Genetic analysis

The initial genetic analysis will consist of high throughput genotyping of the LRRK2 G2019S mutation together with genotyping for the common glucocerebrosidase (GBA) variants associated with PD. It is estimated that approximately 5% of participants in the PD STAT study will carry LRRK2 or GBA mutations. Clearly, specific drug trials and new hypotheses will lead to new areas of analysis and future work will hopefully include whole genome single nucleotide polymorphism (SNP) and, ultimately, exome/genome analysis.

16.3.2 Pharmacogenomic analysis

In collaboration with the PD STAT team and The Cure Parkinson’s Trust, the UCL team will investigate whether specific major genetic sub-groups of PD or specific variants in candidate genes influence the outcome of PD drug trials in a preliminary pilot analysis. This will initially be a post-hoc analysis on a very small number of subjects.

16.3.3 Other analyses

Other analyses may include:

- Screening and analysis of potential pathogenic and anonymous genetic variations in sporadic and familial patients, with comparison to control samples. This will include DNA variants such as point mutations, gene re-arrangements, deletions/duplications, non-coding sequence change and DNA expansions. Analysis will include large-scale SNP analysis and sequence analysis.

- Analysis of phenotype modifiers i.e. analysis of gene variants which modify the disease by altering age at onset, or other disease phenotypes such as drug responsiveness, or secondary characteristics such as cognitive impairment and psychiatric illness.

16.4 Sample and data sharing

All samples will be treated as a gift for research. UCL samples will be stored and used in ongoing and future projects by the PD-STAT genetic sub-study investigators (Dr Camille Carroll and Professor Huw Morris) and their teams. Samples will form part of the Cure Parkinson’s Trust DNA

bank (currently within the CANDAS biobank) which will be made available as a resource for the Parkinson's research community through a vetted application process. A committee including core investigators, scientific, lay and charity representatives will review requests for data and sample access. These samples will be made available to responsible investigators in the UK and around the world for use in research, teaching, therapeutics and diagnostic purposes.

16.5 Confidentiality

Blood samples for the genetics sub-study will be labelled with the participant's unique PD STAT study number ensuring the pseudonymity of the participants who have provided the samples. At UCL, brief clinical details will be stored with the genotype data including date of birth, gender, age at onset of PD, family history and ethnicity. Genotype results will be stored on a web-based, secure confidential database, including after completion of the PD STAT study. Participants may ask for their information to be removed from this database at any time, in accordance with the Data Protection Act 1998 and General Data Protection Regulation, 2018.

Genetics sub-study samples will be linked to the main trial data held by Dr Carroll's team at the end of the study via the unique PD STAT study number to integrate genetic, clinical and trial outcome data as secondary analyses following the main trial. This is essential for analysis of phenotype modifiers - analysis of genes which modify the disease by altering age at onset or other disease phenotypes such as drug responsiveness, or secondary characteristics such as age at onset, response to therapy, and motor phenotype. Participants will be informed of this in the information sheet. Any information collected during the study will be kept confidential, aside from enabling the research team to inform participants about the development of new tests if participants have agreed to this as part of the consent process.

Pseudonymised (de-identified) information and DNA collected during the study may be transferred both within and outside the European Economic Area as part of ongoing collaboration with other researchers. This may include combining data from participants' samples with those of other patients in order to determine important factors related to Parkinson's. This information may be made available to other researchers to enable large-scale analysis and new discoveries. Pseudonymised (de-identified) data will be hosted centrally through a secure web-based database holding research data without personal details. This will meet high security standards and safety measures, including ISO27001 certification, and will enable sharing of data to approved groups. Participants are informed of this in the PD STAT genetics PIS and will consent to these specific aspects. Personal data will be held separately from research data on a separate, secure web-based database meeting the same security standards. Written records linking participants' study numbers with personal identifiable information (e.g. contact details for future communication) will be stored securely in locked filing cabinets.

16.6 Follow-up and future contact

During the consent process for the sub-study, participants will be asked if they would be happy to be contacted in the future to provide further samples or details about their Parkinson's or to learn about new tests or research studies for which they may be eligible.

16.7 Withdrawal

Participation in the genetics sub-study is voluntary and participants can chose to withdraw at any time. If participants decide not to take part or to withdraw from the genetics sub-study, participation and treatment in the main study will not be affected. If participants withdraw from the main study (withdrawal from treatment +/- withdrawal from follow-up), their data and samples will be retained for further use as described in the sub-study participant information sheet. If participants request that their samples and data be withdrawn from the genetics sub-study, every effort will be made to destroy

samples and data that have been provided but in some cases this may not be possible, e.g. when further analyses have been carried out by collaborators.

17 PARTICIPANT FOLLOW UP

17.1 Practically–defined OFF state

The MDS-UPDRS part III and the timed motor tests at baseline, 12, 24 and 26 months need to be conducted in the absence of the participant's regular PD medication so that the severity of the underlying disease is evident. Visits should be held in the morning and participants should attend having omitted their prescribed short acting PD medications (e.g. Levodopa or Ropinirole Immediate release) from 1800h on the day before the clinic visit. Long acting agents (e.g. Ropinirole XL) should be omitted the day before the clinic visit and also on the day of the clinic visit itself. To reduce any physical discomfort of stopping medication and to facilitate attendance at clinic, the local research team should make arrangements to provide the participant with a prescription for relevant supportive medications (e.g. Zopiclone/Zolpidem for night sedation, paracetamol for pain relief and/or diazepam for treatment of dystonia) as necessary. Participants may also be prescribed dispersible Madopar as a rescue medication to be taken in the event of severe difficulty with wearing-off symptoms. A single dose of 62.5mg should be sufficient but up to 125 mg may be prescribed as necessary. If a participant has taken dispersible Madopar after omitting his/her regular PD medication prior to a study visit, the study visit should be rescheduled. If further attempts at attending in the OFF state fail, the participant should be withdrawn from the study.

17.2 Telephone contact 2 (Time 2 weeks)

Participants will be telephoned by a member of the local research team approximately two weeks after the baseline visit to discuss any problems encountered with the study medication, AEs or compliance, as well as any changes to their routine medication. The date and time of the 1 month clinic visit will normally be confirmed at this point and participants should be reminded to bring their diary and their unused study medication with them to that visit.

17.3 Visit 3: 1 month follow-up clinic

Participants will attend a clinic visit one month after the baseline visit, having taken all medications as usual. The PI or authorised delegate will complete the following procedures with the participant in conjunction with study diary review:

- Record any AEs and changes to concomitant medications
- Assess compliance with study treatment and collect any unused medication

Providing that there are no immediate clinical contra-indications, the participant will be provided with a prescription for the higher dose (80mg simvastatin or placebo equivalent daily) of study medication and be provided with a five month supply. Participants who have been unable to tolerate the medication during the lower dose phase will not be prescribed further trial medication for the remainder of the study but will be invited to continue with study assessments.

If formal stopping criteria are fulfilled, study treatment will be discontinued but the participant will be invited to continue with study assessments. Participants should be advised to contact the study team promptly should they develop unexplained muscle pain, tenderness or weakness at any stage.

17.4 Visit 4: 6 month follow-up clinic

Participants will attend a clinic visit six months after the baseline visit, having taken all medications as usual. The PI or authorised delegate will complete the following procedures with the participant in conjunction with study diary review:

- Record any AEs and changes to concomitant medications
- Assess compliance with study treatment and collect any unused medication

Providing that there are no immediate clinical contra-indications, the participant will be provided with a further six month supply of study medication at the prescribed dose. Participants who report prior or during the 6 month visit that they are unable to tolerate the higher dose medication, but do not fulfil the stopping criteria at the 6 month clinic visit can be prescribed the lower dose and continue in the study. Participants who fulfil the stopping criteria will be discontinued from the study treatment but invited to continue with study assessments. Participants should be advised to contact the study team promptly should they develop unexplained muscle pain, tenderness or weakness at any stage.

17.5 Visit 5: 12 month follow-up clinic

Participants will attend a clinic visit 12 months after the baseline visit. For this visit, participants will be asked to omit relevant PD medication so that they attend on the day of assessment in the ‘practically-defined OFF’ state (see 16.1).

The assessor will complete the following procedures with the participant:

- MDS-UPDRS part III (motor examination – see Appendix 5)
- Timed motor tests (finger tapping, EMS (in selected centres) and timed walk test)

Participants will be invited to take their routine PD medication following the motor assessments. The following assessments should then be completed in the practically-defined ON state:

- MDS-UPDRS (parts 1A, III (see Appendix 5), IV)
- EMS (in selected centres)
- MADRS
- NMSS
- ACE-III
- King’s PD pain scale

Following a brief explanation of each measure, participants will be asked to complete the MDS-UPDRS (parts 1B and II), the PDQ-39 and the EQ-5D-5L.

The PI or authorised delegate will complete the following procedures with the participant in conjunction with study diary review:

- Record any AEs
- Record changes to concomitant medications (including PD drugs, to allow LED calculation)
- Assess compliance with study treatment and collect any unused medication
- Take/arrange blood sample(s) for cholesterol and DNA (if the participant has consented to take part in the genetics sub-study)

Providing that there are no clinical contra-indications, the participant will be provided with a further six month supply of study medication at the appropriate dose. Once available, the blood results must be documented in the CRF and reviewed by the local PI to check for any abnormalities in accordance with local reference ranges (as per local site policy/procedure) and the formal stopping criteria for the study (see section 17.8). Out of range blood results should be recorded in the adverse events section of the CRF and participants should be advised to contact the study team promptly should they develop unexplained muscle pain, tenderness or weakness at any stage.

17.6 Visit 6: 18 month follow-up clinic

Participants will attend a clinic visit 18 months after the baseline visit, having taken all medications as usual. The PI or authorised delegate will complete the following procedures with the participant in conjunction with study diary review:

- Record any AEs and changes to concomitant medications
- Assess compliance with study treatment and collect any unused medication
- Take blood samples for the genetic sub-study if required

Providing that there are no clinical contra-indications, the participant will be provided with a further six month supply of study medication at the appropriate dose. Participants should be advised to contact the study team promptly should they develop unexplained muscle pain, tenderness or weakness at any stage.

17.7 Visit 7: 24 month follow up clinic

Participants will attend a clinic visit 24 months after the baseline visit. For this visit, participants will be asked to omit relevant PD medication so that they attend on the day of assessment in the ‘practically-defined OFF’ state (see 16.1).

The assessor will complete the following procedures with the participant:

- MDS-UPDRS part III (motor examination – see Appendix 5)
- Timed motor tests (finger tapping, EMS (in selected centres) and timed walk test)

Participants will be invited to take their routine PD medication following the motor assessments. The following assessments should then be completed in the practically-defined ON state:

- MDS-UPDRS (parts 1A, III (see Appendix 5), IV)
- EMS (in selected centres)
- MADRS
- NMSS
- ACE-III
- King’s PD pain scale

Following a brief explanation of each measure, participants will be asked to complete the MDS-UPDRS (parts 1B and II), the PDQ-39 and the EQ-5D-5L.

The PI or authorised delegate will complete the following procedures with the participant in conjunction with study diary review:

- Record any AEs
- Record changes to concomitant medications (including PD drugs, to allow LED calculation)
- Assess compliance with study treatment and collect any unused medication
- Take/arrange blood sample for cholesterol levels and glycated haemoglobin (HbA1c), and for the genetic sub-study if required

Participants will be reminded to return all empty bottles or unused medication.

17.8 Visit 8: 26 month follow up clinic

Participants will attend a final clinic visit 26 months after the baseline visit. For this visit, participants will be asked to omit relevant PD medication so that they attend on the day of assessment in the ‘practically-defined OFF’ state (see 16.1).

The assessor will complete the following procedures with the participant:

- MDS-UPDRS part III (motor examination)
- Timed motor tests (finger tapping, EMS (in selected centres, if not completed at 24 month visit) and timed walk test)

Participants will be invited to take their routine PD medication following the motor assessments. The following assessments should then be completed in the practically-defined ON state:

- MDS-UPDRS (parts 1A, III, IV)
- EMS (in selected centres, if not completed at 24 month visit)
- MADRS
- NMSS
- ACE-III
- King's PD pain scale

Following a brief explanation of each measure, participants will be asked to complete the MDS-UPDRS (parts 1B and II), the PDQ-39 and the EQ-5D-5L.
The PI or authorised delegate will complete the following procedures with the participant in conjunction with study diary review:

- Record any AEs
- Record changes to concomitant medications (including PD drugs, to allow LED calculation)
- Assess compliance with study treatment and collect any unused medication
- Take/arrange blood sample for cholesterol levels, and for the genetic sub-study if required
- Diary collection

Once available, the blood results must be documented in the CRF and reviewed by the local PI to check for any abnormalities in accordance with local reference ranges (as per local site policy/procedure). Out of range blood results should be recorded in the adverse events section of the CRF.

17.9 Telephone contacts: 2, 4, 8, 10, 14, 16, 20 and 22 months post baseline

Participants will be telephoned by a research nurse or other member of the research team at eight scheduled time points between the baseline visit and the final follow up clinic visit. The purpose of this telephone contact between visits is to identify any compliance problems, AEs or changes to participants' routine medication. Additional telephone contacts may be made as required at the discretion of the local research team and specifically in the event of abnormal blood results being identified at any stage during the trial. Telephone follow-up calls should not be made by the assessor, to preserve blinding.

During the 4, 10, 16 and 22 month telephone contacts the date of the next clinic visit will normally be confirmed and the participant will be reminded to bring their diary, any unused study medication and empty medication bottles to the next visit. At the 10 and 22 month telephone contact participants will also be reminded to omit relevant PD medication so that they attend the 12 and 24 month visits for assessment in the 'practically-defined OFF' state (see 16.1).

17.10 Flexibility of visit schedule

The trial schedule is shown in Appendix 3. A maximum of eight weeks is permitted between the screening and baseline visits. If a baseline visit is unable to be scheduled within this period, the screening visit and assessments should be repeated. Participants should attend the one month follow-up visit at least four weeks, and no later than six weeks, after starting trial treatment. Follow-up visits at 6, 12, 18, 24 and 26 months should ideally be held at +/- two weeks of the due date.

Telephone contact at two weeks should be carried out +/- four days of the due date, and for subsequent telephone contacts +/- seven days of the due date. Compliance with the study visit schedule will be monitored by the CTU.

17.11 Flexibility of follow-up visit location

Every effort will be made for participants to attend follow-up in their local clinic. However, in exceptional circumstances where a participant is unable to attend clinic, site staff may offer a home visit, if their local Trust policy allows.

18 TRIAL TREATMENTS

18.1 Active and comparator treatments

The active investigational medicinal product is simvastatin. Active trial medication will be provided as simvastatin 40mg with cellulose microcrystalline powder in capsule form, to be taken orally. The comparator is a matched placebo capsule containing cellulose microcrystalline powder only.

18.2 Packaging and labelling

The trial treatment and comparator will be presented identically in sealed containers of 100 capsules with child resistant, tamper-evident lids. Containers will be labelled in accordance with regulatory requirements and the contents (active or placebo) will not be outwardly identifiable.

18.3 Provision and storage of trial treatments

Active and placebo medication will be supplied by the Pharmacy Production Department, Royal Free Hospital, London, and distributed to sites by the study's central coordinating pharmacy at Derriford Hospital, Plymouth. Trial medication will be replenished at sites as required throughout the study using a study-specific computerised stock control system maintained by the CTU. Trial medication will be stored at room temperature.

18.4 Prescription and dispensing of trial treatment

For each prescription visit, the PI or authorised delegate will complete and sign a study-specific prescription form for the study medication, including the participant's study number, initials and date of birth. At visits 3, 4, 5 and 6, the PI (or authorised delegate) will access a web-based drug management system provided by CTU to record the dose of study medication prescribed. This will generate a list of allocated bottle numbers for the next treatment period, which should be printed and presented to pharmacy with the study-specific prescription.

18.5 Drug accountability

Site pharmacists will have access to the web-based drug management system in order to track receipt, allocation and return of trial medication. Original prescriptions and printed records of bottle allocations will be kept in a file within each site pharmacy. Participants will be asked to return all empty, full or partially used medication bottles at each study visit. These should be returned to the local site pharmacy for capsule count as part of the assessment of compliance with study treatment.

18.6 Treatment schedule and supply to participants

Medication will be prescribed in two phases: a lower dose phase of 40 mg active drug/equivalent placebo for one month and a higher dose maintenance phase of 80 mg active drug/equivalent placebo for 23 months. Participants will be provided with a one month supply of study medication at baseline (visit 2), a five month supply at month 1 (visit 3) and a six month supply at months 6, 12 and 18 (visits 4, 5 and 6). Participants will be asked to return any unused medication and empty medication bottles at the next study visit. Medication should be taken in the evening. Participants will be given written advice about how to take the study medication, and its potential side effects.

18.7 Monitoring of trial treatment safety and tolerability

Side effects and tolerability of trial medication will be reviewed during clinic visits at 1 month, 6, 12, 18 and 24 months. In between scheduled study visits, participants will be telephoned by a member of their local site research team at approximately 2, 4, 8, 10, 14, 16, 20 and 22 months. Tolerability will be defined as the ability of participants to remain on the allocated treatment. In the case of unwanted side effects, the defined treatment schedule can be altered as outlined below.

At each visit or telephone call, participants will be asked about any adverse events experienced and, dependent on reported symptoms, may be asked to provide a blood sample to check CK and/or AST/ALT levels. If a raised AST/ALT is observed in the absence of a CK result, the CK should be checked. Tables 1-3 outline the possible outcomes and any action required.

If the participant reports jaundice or new or unusually severe nausea, malaise or lethargy, an AST/ALT level should be checked. Refer to Table 1 for potential outcomes and action required. If study treatment needs to be stopped temporarily, AST/ALT should be checked again in six weeks' time and action taken in accordance with Table 2.

If the participant reports new or unusually severe muscle pain, tenderness or weakness, the CK level should be checked. Refer to Table 3 for potential outcomes and action required.

Table 1: AST/ALT monitoring outcomes and action required

Observation	Action required	Repeat observation	Action required
AST/ALT >3xULN	Repeat sample within 1 week.	AST/ALT >4xULN	Stop study treatment temporarily.
		AST/ALT >2xULN but ≤4 x ULN	Repeat again within 3 weeks. If remains >2xULN stop study treatment temporarily.
AST/ALT >2xULN but ≤3xULN	Repeat again within 3 weeks.	AST/ALT >3xULN	Stop study treatment temporarily.
		AST/ALT >2xULN but ≤3xULN	Repeat again within 3 week. If remains >2xULN stop study treatment temporarily.

Table 2: AST/ALT monitoring outcomes and action required 6 weeks following temporary stop of study treatment

Observation	Action required	Subsequent action required
AST/ALT >1.5xULN	Stop study treatment permanently.	Repeat every 3 weeks until AST/ALT reverts to normal (i.e. ≤1.5xULN)
AST/ALT ≤1.5xULN	Study treatment can be restarted.	Repeat twice at 3 week intervals. AST/ALT must remain ≤2xULN, otherwise study treatment should be stopped permanently.

Table 3: CK monitoring outcomes and action required

Observation	Action required	Repeat observation	Action required
CK >5xULN	**Stop study treatment permanently.** Repeat sample within 1 week.	Repeat every 3 weeks until CK reverts to normal (i.e. ≤3xULN)	Repeat every 3 weeks until CK reverts to normal (i.e. ≤3xULN)
CK >4xULN but ≤5xULN that cannot be explained (i.e. trauma, heavy exercise etc.)	Repeat sample within 1 week.	CK remains >4xULN but ≤5xULN.	Stop study treatment temporarily.<br>Check CK again in 6 weeks;<br>• If CK >3xULN stop study treatment permanently.<br>• If CK ≤3xULN study treatment can be restarted with 2 further repeats at 3 week intervals (at which CK must remain ≤3xULN otherwise study treatment must be stopped permanently.

18.8 Stopping criteria for discontinuation of trial treatment

The defined stopping criteria for the discontinuation of trial medication are:

(1) Abnormalities in CK or ALT/AST fulfilling stopping criteria as outlined in Section 17.7, OR
(2) New severe muscular symptoms (progressive or persistent), not attributable to other cause, which in the opinion of the PI may be related to the study medication even in the absence of abnormal CK.
(3) Onset of a clinical condition for which prescription of a statin is indicated

18.9 Dose adjustments

Dose adjustments may be made at clinic visits (e.g. in the event of reported unwanted side effects) or at an interim point (e.g. following review of blood results or clinical discussion). In the latter situation, a member of the research team should contact the participant (preferably by telephone but followed by written confirmation) with instructions about any dose change or need to stop trial medication.

18.9.1 Dose alteration during lower dose phase

During the one month lower dose phase, participants who are unable to tolerate the 40mg dose of study medication due to unwanted symptoms, or who fulfil the stopping criteria, will have their trial treatment permanently discontinued but will be invited to continue with the study assessments. Additional patients will be recruited to the study to allow for those who are unable to tolerate 40mg study medication daily.

18.9.2 Dose alteration during higher dose phase and dose re-challenge

During the higher dose maintenance phase, participants who are unable to tolerate the 80mg dose of study medication due to unwanted symptoms (but do not fulfil the stopping criteria) may have their dose reduced to 40mg daily. Participants may continue on the 40mg dose for the remainder of the trial or, at the discretion of the local investigator, participants may later be re-challenged with the 80mg dose after resolution of their symptoms. Participants who fulfil the stopping criteria at 80mg will be withdrawn from treatment but invited to continue with the study assessments.

18.9.3 Temporary/permanent cessation of treatment due to contraindicated medication

Participants who are prescribed any medication contraindicated for use with simvastatin (see Appendix 2) must inform their local research team and must cease taking trial medication for the period in which the contraindicated medication is taken. In the case of short courses of contraindicated antibiotic or antifungal treatment, trial medication may be recommenced five days or as otherwise specified in the SmPC for Simvastatin after completing the course of treatment. The local site pharmacy should be consulted for the latest version of the SmPC for Simvastatin.

19 PHARMACOVIGILANCE

19.1 Definitions

An adverse event (AE) is defined as any unfavourable and unintended sign, symptom or illness that develops or worsens during the period of the trial, whether or not it is considered to be related to the trial intervention. AEs include unwanted side effects, injury or intercurrent illnesses and abnormal blood results and may be expected or unexpected. An AE that is considered to have a suspected causal relationship to the trial intervention is defined as an **adverse reaction**.

An AE is classified as a **Serious Adverse Event (SAE)** if it:

- results in death
- is life threatening
- requires hospitalisation or prolongation of existing hospitalisation
- results in persistent or significant disability or incapacity
- consists of a congenital anomaly or birth defect
- or is considered by the investigator to be an important medical event

19.2 Reporting non-serious adverse events

Only those non-serious AEs considered by the PI or authorised delegate to be possibly, probably or definitely related to trial treatment will be reported in this study.

AEs will be recorded by the research nurse or other member of the research team in the CRF and also in the participant’s hospital case notes. AEs should be recorded from the time a participant gives written consent for the study until the date the participant completes follow-up or withdraws from the study. AEs may be identified during follow-up visits, telephone contacts or as a result of direct reporting by the participant, non-study clinician or other informant at any time during the study. Multiple symptoms should be recorded as separate events. The PI or authorised delegate is responsible for assessing the relationship between each adverse event and the trial treatment. Completed AE forms should be returned to the CTU as directed in the CRF. In the event that a participant reports an adverse event necessitating discontinuation of study medication, the local research team should maintain regular telephone follow-up with the participant to monitor resolution of the adverse event.

19.3 Reporting serious adverse events

Any **SAE**, whether thought to be related to trial treatment or not, must be reported to the CTU by the

local PI or another member of the research team by telephone (01752 315256) or fax (01752 315245) within 24 hours of the research team becoming aware of it. The CTU will routinely notify the Chief Investigator (CI) by email of all reported SAEs. Within 7 days of the local research team becoming aware of such an event, an SAE form must be completed*, signed by the PI and returned to the CTU. If incomplete information is available at the time of reporting, all appropriate information relating to the SAE should be forwarded to the CTU as soon as possible. Completion of the SAE form must include the PI's assessment of causality i.e. whether there is a reasonable causal relationship between the SAE and trial treatment. If the PI considers that the SAE is not, or unlikely to be, related to trial treatment, the CTU will obtain a second assessment of causality from the CI (or other nominated clinician). If, in the opinion of either assessor, the SAE is considered possibly, probably or definitely related to trial treatment (Serious Suspected Adverse Reaction, SSAR), the CI or nominated deputy will assess the expectedness of the SAE with reference to the Summary of Product Characteristics (SmPC) for simvastatin. A SSAR which is not consistent with the information set out in the SmPC for simvastatin is classified as a SUSAR (see section 17.4).

All SAEs will be followed until resolution and the CTU will report organ system listings of all SAEs to the Data Monitoring Committee (DMC) and Sponsor on a quarterly basis.

19.4 Suspected Unexpected Serious Adverse Reaction

All suspected adverse reactions that are both serious and unexpected (SUSARs) are subject to expedited reporting.

If the CTU is notified of a SAE which qualifies as a potential SUSAR, details will immediately be passed to the study Sponsor. Current European guidelines recommend that the treatment code be broken by the study Sponsor before a SUSAR is reported to the relevant authorities. In the case of a suspected SUSAR, the Sponsor will have the facility to unblind the treatment allocation independently of the CI and CTU trial manager in order to preserve blinding within the Trial Management Team.

The Sponsor will report any SUSAR which is **fatal or life-threatening** to the Medicines and Healthcare products Regulatory Agency (MHRA) and relevant ethics committee **not later than 7 days** after the Sponsor is first made aware of the reaction. The Sponsor is responsible for forwarding relevant follow-up information to the MHRA and Research Ethics Committee (REC) within an additional eight days.

SUSARs which are **not** fatal or life-threatening will be reported by the Sponsor to the MHRA and relevant REC as soon as possible and not later than 15 days after the Sponsor first becomes aware of the reaction. The Sponsor will forward follow-up information to the MHRA and REC as soon as possible.

All potential SUSARs will be reported to the DMC as they occur and all events will be followed until resolution. In the event of a potential SUSAR at any participating site, the PIs at all sites will be notified of the event in a blinded manner. There is no routine requirement for the study Sponsor to report SAEs other than SUSARs to the MHRA and REC. However, the Sponsor is obliged to submit an annual safety report (Development Safety Update Report) to the MHRA and REC, including listings of all suspected serious adverse reactions.

20 UNBLINDING

Participants will be blind to treatment allocation throughout the trial, as will the trial management team, investigator site teams and site pharmacy staff. To preserve blinding as far as possible, members of site research teams assigned to undertake outcome measures assessment should not be involved in monitoring adverse events or titration of study medication dose. In the event of a

potential SUSAR, unblinding will be undertaken by the Sponsor in accordance with the regulatory requirements for safety reporting in Clinical Trials of Investigational Medicinal Products (CTIMPs). Unblinding may also be performed at the request of a senior clinician responsible for the care of a trial participant but such requests are likely to occur only in the case of a serious adverse clinical event and are expected to be rare. During normal working hours, any request to unblind treatment allocation for clinical reasons should be made directly to the PenCTU. Out of hours requests for unblinding should be made via the central coordinating pharmacy at Derriford Hospital according to a written procedure available in the Investigator Site File. In either case, the participant's treatment allocation will be reported directly to the relevant clinician according to the agreed procedure. The coordinating pharmacy and CTU will maintain a record of all unblinding requests. The CI and CTU trial manager will be kept informed of all instances of unblinding but remain blind to treatment allocations themselves wherever possible

21 SUBJECT WITHDRAWAL

Participants will normally complete the study after the 26 month follow-up clinic. The trial itself will end on the date that the last participant attends his/her last study visit. A participant may, at any time, withdraw from trial treatment and/or follow-up without giving a reason and without it affecting their relationship with the clinical team, their future treatment or care.

21.1 Withdrawal from treatment

Participants may choose to withdraw from trial treatment at any stage of the trial without giving a reason and without their usual care being affected. Participants may also have their trial treatment formally discontinued by the PI or authorised delegate for safety reasons, as described in section 16. Withdrawal from trial treatment, and the reason, if known, should be clearly documented in the participant's clinical records and reported to the CTU according to the agreed procedure. The trial analysis is based on intention to treat, so all participants will be encouraged to continue with study visits and assessments as per protocol even if trial treatment is discontinued prematurely.

21.2 Withdrawal from follow-up

Although all participants will be encouraged to complete study follow-up, participants may withdraw from follow-up at any time without it affecting their care. Participants should be asked to explain their reason for withdrawing, but are under no obligation to do so. Withdrawal from trial follow-up, and the reason, if known, should be clearly documented in the participant's clinical records and reported to the CTU according to the agreed procedure. Data collected prior to withdrawal from follow-up will be included in the study analysis unless a participant specifically requests that their data are removed from the database.

Efforts will be made to replace participants who discontinue treatment during the low dose phase of the study even if these participants are continuing in follow-up. Participants who discontinue treatment in the higher dose phase will not be replaced.

21.3 Premature termination of study

In the event that the Trial Steering Committee (TSC) or Sponsor recommends early termination of the study for any reason, the CTU will notify the REC and MHRA. The CI will be responsible for informing participants of the premature termination of the study.

22 DATA MANAGEMENT

22.1 Subject numbering

Each participant will be allocated a unique study number on consenting to the study and will be identified in all study-related documentation by their study number and initials.

22.2 Data collection

Data will be recorded on two-part NCR (no carbon required) study specific CRFs by relevant members of the research team at each site. All persons authorised to collect and record trial data at each site will be listed on the study site delegation logs, signed by the relevant PI. As a minimum, a record should also be made in the participant's hospital notes of:-

- Consent and eligibility for study
- Dates of all study visits attended
- Dose of trial medication prescribed, and changes
- Changes to concomitant medication
- Adverse events
- Completion or discontinuation of study

All data not routinely captured in the hospital records but recorded straight into the CRF will be classified as source data.

22.3 Data entry

Completed CRF pages should be checked and signed at the research sites by the research nurse or appropriate member of the research team before original (top) copies are posted to the CTU at agreed timepoints. Data will be double-data entered on to a password-protected database within CTU, with copies retained at the relevant study site. Forms will be tracked using a web-based trial management system. Double-entered data will be compared for discrepancies using a stored procedure. Discrepant data will be verified using the original paper data sheets.

22.4 Data confidentiality and security

Research teams at all sites will ensure that participants' anonymity is maintained on all documents. Data will be collected and stored in accordance with the Data Protection Act, 1998 and General Data Protection Regulation, 2018. Within the CTU, pseudonymised paper-based study data will be stored in locked filing cabinets within a locked office. Electronic records will be stored in a SQL server database, stored on a restricted access, secure server maintained by University of Plymouth. The website will be encrypted using SSL. Direct access to the trial data will be restricted to members of the research team and the CTU, with access granted to the Sponsor on request. Access to the database will be overseen by the CTU data manager and trial manager. Copies of study data retained at study sites will be securely stored for the duration of the study prior to archiving.

After the end of the study, pseudonymised information collected during the study will be made available to other researchers under an appropriate data sharing agreement, but it will not be possible to identify participants personally from any information shared.

Electronic data relating to the PD STAT genetics sub-study will be stored on a secure database held on a web application (REDCap), consisting of PHP software with a MySQL database back-end. REDCap is password protected, includes audit trails of any activity, and is encrypted using SSL. The database will be hosted on a secure cloud hosting service. The web host, network connection and storage is Information Governance Toolkit compliant and ISO27001 certified following data security best practice. Personally identifiable information will be held on a database which is separated from the main study database, with access restricted to Professor Huw Morris and the PD STAT Genetics sub study team at UCL. The PD STAT genetics sub-study team will be responsible for enforcing a secure and robust system for maintaining coherent de-identified participant IDs at the central database. Personal information data transfer will be needed for registration of patients with the NHS Health and Social Care Information Service for tracking of mortality and death certificate data. Data will be transferred in AES-256 encrypted format.

22.5 Archiving

Following completion of trial data analysis, the Sponsor will be responsible for archiving the study data and essential documentation in a secure location for a period of 15 years after the end of the trial. No trial-related records should be destroyed unless or until the Sponsor gives authorisation to do so. Medical case notes containing source data or other trial-related information should be identified by a label "Keep until dd/mm/yyyy" where the date given is 15 years after the last participant's final study visit.

23 STATISTICAL CONSIDERATIONS

Statistical analysis will be carried out by University of Plymouth statisticians at the end of the study (following last participant's last visit). A full statistical analysis plan will be developed and agreed by the DMC and TSC before analysis begins.

23.1 Sample size

In futility studies, the direction of the hypotheses is different from that in traditional phase III efficacy or effectiveness trials. The study sample size has been calculated based on testing the **null hypothesis that simvastatin is not futile**, in terms of the primary outcome - change in MDS-UPDRS part III motor subscale score in the OFF state at 24 months. If at the end of the study there is evidence to reject the null hypothesis, then simvastatin will be considered to be futile for a phase III study, although the final decision on progression to a phase III study will be made in discussion with the DMC and TSC.

The minimum clinically important difference in UPDRS motor score has been estimated to be 2.3-2.7 (Shulman et al., 2010). The null hypothesis (H_0) in this futility study is that the mean MDS-UPDRS part III change score for the simvastatin group is at least 3 points better (i.e. smaller, as higher MDS-UPDRS scores are worse) than the corresponding mean change in the placebo group. The alternative hypothesis (H_A) is that the mean MDS-UPDRS part III change score for the simvastatin group is not at least 3 points better. This can be written mathematically as:

$$\mathbf{H_0: \mu_s \leq \mu_p - 3 \text{ vs } H_A: \mu_s > \mu_p - 3}$$

where μ_s is the expected mean MDS-UPDRS part III change score from baseline to 24 months for the simvastatin group and μ_p is the expected mean change for the placebo group. Therefore a one-sided test (and associated alpha) is appropriate.

In futility studies, the error probabilities are interpreted differently from those in traditional efficacy/effectiveness studies. The type 1 (alpha) error is recommending that an effective treatment should not be considered for a phase III study and the type 2 (beta) error is recommending that an ineffective treatment should be considered for a phase III study (Tilley et al., 2006). Given these different interpretations, alpha and beta are chosen relative to the futility design-based hypotheses: in this study, one-sided alpha is set at 10% and beta at 20% (i.e. 80% power) (Tilley et al., 2006). Under these design parameters, there is a 20% chance of failing to identify that simvastatin is ineffective.

Based on a recently reported trial, the expected mean increase in MDS-UPDRS part III from baseline to 12 months is 2.2 points and standard deviation 7.3 in the placebo group (Aviles-Olmos et al., 2013). Assuming that this increase in MDS-UPDRS part III is linear over time gives an expected mean increase from baseline to 24 months of 4.4 points in the placebo group. Additionally, we assume a slightly inflated standard deviation over this period of 7.5.

The null hypothesis H_0: $\mu_s \leq \mu_p - 3$ can be stated equivalently as H_0: $\mu_s - \mu_p \leq -3$. To test this hypothesis, and assuming μ_p is 4.4 points, it is assumed that μ_s is 1.4 points (i.e. 4.4 – 3). It is

estimated that based on a two sample t-test with a 10% one-sided alpha, a sample size of 57 patients per group will provide 80% power to reject the null hypothesis and declare futility.

This sample size is firstly inflated to allow for a small proportion of participants allocated to the simvastatin group to stop taking the trial medication either during the initial four week low dose phase. Assuming that this proportion is 15%, the above sample size is inflated by a factor of $1/(1–0.15)^2$, to give 79 participants per group (Redmond and Colton 2001). Secondly the sample size was adjusted to allow for a (non-differential) loss to follow-up rate of 20%. Accordingly, the sample size was further inflated by a factor of 1/(1-0.2), to give a sample size of 99 participants per group and a total recruitment target of 198 participants commencing the higher dose phase of the study.

23.2 Statistical analysis

The study will be reported following the relevant Consolidated Standards Of Reporting Trials (CONSORT) guidelines. There is no planned interim analysis for this study. Primary analyses will be on an intention to treat (ITT) basis. The ITT evaluable sample will include all subjects who are randomised and who commence on the higher dose phase of the study. As this is a phase II study, no imputation of missing data is planned for the primary analysis.

The statistical analyses will be undertaken blinded to the allocated group. The primary analysis will be a between-group comparison of mean change in MDS-UPDRS part III from baseline to 24 months. Specifically, a linear regression model will be fitted to MDS-UPDRS part III scores at 24 months, with allocated treatment group, baseline MDS-UPDRS part III score the stratification variable (modified Hoehn & Yahr stage), gender and age at baseline included as covariates. Scores will be suitably transformed if necessary. In the primary analysis of the primary outcome, if the p-value from the regression model for the adjusted treatment effect is <0.1, then the null hypothesis that simvastatin is not futile will be rejected and simvastatin will be considered to be futile for a phase III study. For completeness, the two-sided 80% confidence interval for the estimated treatment effect will also be presented, although only the upper bound of the confidence interval is of relevance when assessing for futility. If the upper bound of the confidence interval is lower than -3, there will be evidence to consider simvastatin for a phase II study (Appendix 4).

Secondary continuous outcomes will be compared between treatment groups in a similar way, although will not be statistically tested for futility; instead the focus will be on providing appropriate summary statistics and confidence intervals for the between-group differences. Ordered categorical outcomes will be analysed using ordinal regression analysis.

Consideration will be given to a secondary analysis of the primary outcome on a per-protocol basis. If a sufficient number of patients drop back down to the lower dose of simvastatin, consideration will be given to a sub-group analysis of the effect of dose. These, and any other secondary analyses, will be discussed with the DMC and included in the statistical analysis plan.

24 DATA MONITORING AND QUALITY ASSURANCE

The research nurse or other member of the research team will check completed CRFs for missing data or obvious errors before forms are sent to the CTU. Data will be monitored centrally for quality and completeness by the CTU and every effort will be made to recover data from incomplete forms where possible. The CTU data manager will oversee data tracking and data entry and initiate processes to resolve data queries where necessary. The CTU trial manager will devise a risk-based monitoring plan specific to the study which will include both central monitoring strategies and study site visits as appropriate.

All trial procedures will be conducted in compliance with the protocol and according to the principles of Good Clinical Practice. Procedures specifically conducted by the CTU team (e.g. randomisation, data management, trial management and study monitoring) will be conducted in compliance with CTU standard operating procedures (SOPs). The PIs and the participating NHS Trusts will be required to permit the CTU trial manager or deputy to undertake trial-related monitoring to ensure compliance with the approved trial protocol and applicable SOPs, providing direct access to source data and documents as requested.

25 TRIAL OVERSIGHT

The CI will be responsible for the overall running of the trial and for the local conduct of the trial at the Plymouth site. The PI at each of the other participating sites will be responsible for the conduct of the study at his/her trial site. The CTU will coordinate trial-related activities and assist with overall trial management, monitoring and production of progress reports. The CTU will also organise the web-based randomisation, prepare the database, provide double data entry into the database, and oversee safety reporting activities.

25.1 Trial Management Group (TMG)

A trial management group (TMG) including the CI, CTU trial managers, trial statistician and other personnel relevant to the study (e.g. clinicians, clinical trials pharmacist, CTU data manager, patient and Sponsor representatives) will meet regularly (usually monthly) throughout the duration of the trial to monitor progress, resolve day-to-day problems, oversee development of study documentation, monitor participant recruitment and retention, assess data quality, review budgetary issues, discuss analysis, interpret study findings, draft reports and plan dissemination of results.

25.2 Trial Steering Committee (TSC)

The TSC will oversee the conduct and safety of the trial, ensuring that milestones are achieved and general scientific probity is maintained. The Committee will include an independent chair (Prof Cathie Sudlow, neurologist), an independent statistician (Dr Obioha Ukoumunne), an independent neurologist, two lay representatives, a representative from the Cure Parkinson's Trust, Prof John Zajicek (neurologist, co-applicant) and the CI. The trial statistician, trial manager(s) and Sponsor representative will be invited to meetings as observers. Terms of reference for the TSC will be agreed before the start of the study and incorporated into a TSC charter, updated from time to time as required. The TSC will meet once before the start of the study and approximately once a year thereafter.

25.3 Data Monitoring Committee (DMC)

An independent DMC will monitor the safety and ethics of the trial by overseeing recruitment, primary outcome data completeness and AEs. Operating procedures for the DMC will be agreed before the start of the study and incorporated into a DMC charter, updated from time to time as required. The committee will be chaired by Dr Donald Grosset (neurologist) and the other members will be Dr Jeremy Chataway (neurologist) and Dr Chris Metcalfe (statistician). The committee will meet once before the start of the trial and approximately annually thereafter, by teleconference or face-to-face.

26 ETHICS AND REGULATORY APPROVALS

26.1 Sponsor

The study Sponsor is University Hospitals Plymouth NHS Trust (UHPNT) although financial oversight will be the responsibility of University of Plymouth. Selected sponsorship tasks will be formally delegated to the Peninsula Clinical Trials Unit at the University of Plymouth (CTU) according to an agreed task allocation matrix.

26.2 Research governance

The study will be undertaken at UHPNT and at additional NHS Trusts, subject to appropriate REC approval, local NHS Research & Development approvals and a Clinical Trials Authorisation (CTA) issued by the Medicines and Healthcare products Regulatory Agency (MHRA). The trial will be conducted in accordance with the study protocol, the principles of the Declaration of Helsinki, International Conference on Harmonisation of Good Clinical Practice (ICH GCP) and the Medicines for Human Use (Clinical Trials) Regulations, 2004. Any amendments of the protocol will be submitted to the REC and regulatory authority for approval. On request, the CI/PI should make available relevant trial-related documents for monitoring and audit by the Sponsor, the relevant REC or the MHRA.

27 STATEMENT OF INDEMNITY

This is an NHS-sponsored research trial. If an individual suffers negligent harm as a result of participating in the trial, NHS indemnity covers NHS staff and those people responsible for conducting the trial who have honorary contracts with the relevant NHS Trust. In the case of non-negligent harm, the NHS is unable to agree in advance to pay compensation, but an ex-gratia payment may be considered in the event of a claim.

28 PUBLICATION POLICY

The study team will prepare a plain English summary of the study results which will be sent to the study participants as soon as possible after the end of the trial. Results of the study may also be presented at meetings of PD support groups or to other relevant lay audiences.

The Chief Investigator will establish a writing committee comprising co-applicants, relevant members of the trial management group and/or others connected with the trial. The committee will be responsible for establishing authorship rules and preparing scientific reports of the study findings. The study results will be submitted for publication in international, high impact, peer reviewed journals relating to neurology and PD. Names of key collaborators and groups who have contributed to the trial will be clearly stated in all publications. The study findings will be presented at regional, national and international meetings as appropriate.

29 FINANCE

The trial is funded by grants from the Cure Parkinson’s Trust and the JP Moulton Charitable Foundation. University of Plymouth is responsible for managing the study budget.

30 REFERENCES

Aviles-Olmos I, Dickson J, Kefalopoulou Z, et al. Exenatide and the treatment of patients with Parkinson's disease. Journal of Clinical Investigation. Jun 2013;123(6):2730-2736.

Brundin P, Barker RA, Conn PJ, et al. Linked Clinical Trials - The development of new clinical learning studies in Parkinson's disease using screening of multiple prospective new treatments. Journal of Parkinsons Disease. 2013;3(3):231-239.

Cederberg H, Stančáková A, Yaluri N, Modi S, Kuusisto J, Laakso M. Increased risk of diabetes with statin treatment is associated with impaired insulin sensitivity and insulin secretion: a 6 year

follow-up study of the METSIM cohort. Diabetologia. 2015;58:1109-1117.

Chataway J, Schuerer N, Alsanousi A, et al. Effect of high-dose simvastatin on brain atrophy and disability in secondary progressive multiple sclerosis (MS-STAT): a randomised, placebo-controlled, phase 2 trial. The Lancet. 2014;383(9936):2213-21.

Chaudhuri KR, Martinez-Martin P, Brown RG, et al. The metric properties of a novel non-motor symptoms scale for Parkinson's disease: Results from an international pilot study. Mov. Disord. Oct 15 2007;22(13):1901-1911.

Chaudhuri KR, Rizos A, Trenkwalder C et al. King's Parkinson's Disease Pain Scale, the first scale for pain in PD: An international validation. Movement Disorders. In press.

Friedman B, Lahad A, Dresner Y, Vinker S. Long-Term statin use and the risk of Parkinson's disease. American Journal of Managed Care. Aug 2013;19(8):626-632.

Ghosh A, Roy A, Matras J, Brahmachari S, Gendelman HE, Pahan K. Simvastatin inhibits the activation of p21(ras) and prevents the loss of dopaminergic neurons in a mouse model of Parkinson's disease. Journal of Neuroscience. Oct 28 2009;29(43):13543-13556.

Goetz CG, Tilley BC, Shaftman SR, et al. Movement Disorder Society-sponsored revision of the Unified Parkinson's Disease Rating Scale (MDS-UPDRS): Scale presentation and clinimetric testing results. Mov. Disord. 2008;23(15):2129-2170.

Hsieh S, Schubert S, Hoon C, Mioshi E, Hodges J, R, Validation of the Addenbrooke's Cognitive Examination III in Frontotemporal Dementia and Alzheimer's Disease. Dement Geriatr Cogn Disord 2013;36:242-250

Lang AE, Melamed E, Poewe W, Rascol O. Trial designs used to study neuroprotective therapy in Parkinson's disease. Mov. Disord. 2013;28(1):86-95.

Leentjens AFG, Verhey FRJ, Lousberg R, Spitsbergen H, Wilmink FW. The validity of the Hamilton and Montgomery-Asberg depression rating scales as screening and diagnostic tools for depression in Parkinson's disease. International Journal of Geriatric Psychiatry. Jul 2000;15(7):644-649.

Lv H-l, Jin D-m, Liu M, Liu Y-m, Wang J-f, Geng D-f. Long-term efficacy and safety of statin treatment beyond six years: A meta-analysis of randomized controlled trials with extended follow-up. Pharmacological Research. 2014;81:64-73.

Naci H, Brugts J, Ades T. Comparative Tolerability and Harms of Individual Statins: A study-level network meta-analysis of 246 955 participants from 135 randomized, controlled trials. Circulation: Cardiovascular Quality and Outcomes. July 1, 2013;6(4):390-399.

NICE 2014. Lipid modification: cardiovascular risk assessment and the modification of blood lipids for the primary and secondary prevention of cardiovascular disease http://guidance.nice.org.uk/CG181.

Noyce AJ, Nagy A, Acharya S et al. Bradykinesia-Akinesia Incoordination Test: Validating an Online Keyboard Test of Upper Limb Function. PLOS ONE. April 2014; Volume 9; Issue 4. e96260.

Peto V, Jenkinson C, Fitzpatrick R, Greenhall R. The development and validation of a short measure of functioning and well-being for individuals with Parkinson's disease. Quality of Life Research. Jun 1995;4(3):241-248.

Redmond CK and Colton T (editors). Biostatistics in Clinical Trials. Wiley 2001.

Roy A, Pahan K. Prospects of statins in Parkinson disease. Neuroscientist. Jun 2011;17(3):244-255.

Saldana, J. (2013) The Coding Manual for Qualitative Researchers. London: Sage.

Schwid SR, Cutter GR. Futility studies: Spending a little to save a lot. Neurology. March 14, 2006;66(5):626-627.

Shulman LM, Gruber-Baldini AL, Anderson KE, Fishman PS, Reich SG, Weiner WJ. The clinically important difference on the Unified Parkinson's Disease Rating Scale. Archives of Neurology. 2010;67(1):64-70.

Tilley BC, Palesch YY, Kieburtz K, Ravina B, et al. Optimising the ongoing search for new treatments for Parkinson disease: using futility designs. Neurology. 2006;66(5):628-633.

Undela K, Gudala K, Malla S, Bansal D. Statin use and risk of Parkinson's disease: a meta-analysis of observational studies. Journal of Neurology. Jan 2013;260(1):158-165.

Villeneuve S, Rodrigues-Brazete J, Joncas S, Postuma RB, Latreille V, Gagnon J-F. Validity of the Mattis Dementia Rating Scale to detect mild cognitive impairment in Parkinson's disease and REM sleep behavior disorder. Dementia and Geriatric Cognitive Disorders. 2011;31(3):210-217.

WHO 2011: http://www.who.int/diabetes/publications/report-hba1c_2011.pdf).

31 APPENDICES

Appendix 1: Description of outcome measures

- The **MDS-UPDRS** is the standard validated tool for the assessment of patients with PD (Goetz et al., 2008). This scale includes subsections collecting data regarding the impact of PD on a patient's mood and mental state, (UPDRS part I), their activities of daily living (UPDRS part II) an examination of the motor features of PD (UPDRS part III), and complications arising from the use of dopamine replacement (part IV).

- **Timed Motor Tests** include evaluating the number of hand taps (key strokes) that an individual can perform within 30 seconds (Noyce et al., 2014) and a timed walk test (10MWT). In 5 recruiting centres, additional electromagnetic sensor (EMS) measurements will be conducted at the 12 month and 24 month follow up visits (Appendix 5).
- **The Addenbrooke's Cognitive Examination-III (ACE)** is one of the most popular and commonly used cognitive tests used in dementia clinics and in the assessment of other neurological disorders. ACE-III includes five subdomains which provide a cognitive score out of a maximum of 100 (Hsieh et al., 2013).
- The **Montgomery and Asberg Depression Rating Scale (MADRS)** is a 10 item physician rated depression severity scale previously used in the assessment of PD (Leentjens et al., 2000).
- The **PDQ39** is a PD-specific health status questionnaire used both clinically and within research since its publication in 1995 (Peto et al., 1995). It consists of 39 items covering eight discrete dimensions: mobility, emotional well-being, stigma, social support, cognition, communication and bodily discomfort. The scores from each dimension are computed into a scale ranging from 0 (best, i.e. no problem at all) to 100 (worst, i.e. maximum level of problem). In addition a summary score, the PDQ-39SI (summary index) can be calculated by averaging the scores of the eight dimensions.
- The **Non-Motor Symptom assessment scale (NMSS)** is a rating scale designed to capture the presence of the non-motor features of PD (Chaudhuri et al., 2007).
- The **King's PD Pain Scale** (Chaudhuri et al., 2015) is a PD-specific scale consisting of 14 items within seven domains. Each item is scored by severity (0-3) multiplied by frequency (0-4), resulting in an item sub-score of 0-12 and a total possible score of 0-168.

Appendix 2: Contra-indicated medications

The table below summarises the prescribing recommendations for agents known to interact with simvastatin.

Table: Drug interactions associated with increased risk of myopathy/rhabdomyolysis

**Interacting agents**	**Prescribing recommendations**
Potent CYP3A4 inhibitors: e.g.<br><br>Itraconazole<br>Ketoconazole<br>Posaconazole<br>Voriconazole<br>Erythromycin<br>Clarithromycin<br>Telithromycin<br>HIV protease inhibitors (e.g. nelfinavir)<br>Bocepreivir<br>Telaprevir<br>Nefazodone<br>Cobicistat<br>Ciclosporin<br>Danazol<br>Gemfibrozil	 <br>Contraindicated with simvastatin
Other fibrates (except fenofibrate)	Do not exceed 10 mg simvastatin daily
Fusidic acid	Is not recommended with simvastatin
Niacin (nicotinic acid) (≥ 1 g/day)	For Asian patients, is not recommended with simvastatin
Amiodarone<br>Verapamil<br>Diltiazem<br>Amlodipine<br>Elbasvir<br>Grazoprevir	Do not exceed 20 mg simvastatin daily
Lomitapide	For patients with HoFH, do not exceed 40 mg simvastatin daily
Grapefruit juice	Avoid grapefruit juice when taking simvastatin

Source: SmPC for simvastatin 40 mg (as updated 01 August 2018)

Appendix 3: Trial Schedule

CONTACTS:	T1	V1	V2	T2	V3	T3	T4	V4	T5	T6	V5	T7	T8	V6	T9	T10	V7	V8
	- 4 weeks	Screen - 2 weeks	Baseline	2 weeks	1 month	2 months	4 months	6 months	8 months	10 months	12 months	14 months	16 months	18 months	20 months	22 months	24 months	26 months
Demographics		X																
Informed consent		X																
Randomisation			X															
Medical history		X																
Physical Exam		X																
Hoehn & Yahr		X																
MoCA		X																
Screening bloods		X																
Serum cholesterol		X									X*						X	X
HbA1c		X															X	
Telephone contact	X			X		X	X		X	X		X	X		X	X		
MDS-UPDRS Part III (OFF)			X								X						X	X
Complete MDS-UPDRS (ON)			X								X						X	X
ACE-III			X								X						X	X
MADRS		X									X						X	X
Timed Motor Tests			X								X						X	X
PDQ-39			X								X						X	X
King's PD pain scale			X								X						X	X
EQ-5D-5L			X								X						X	X
LED			X								X						X	X
NMSS			X								X						X	X
Adverse events			X	X	X	X	X	X	X	X	X	X	X	X	X		X	X
Experience survey			X								X							X
Diary provision			X															
Diary review					X			X			X			X			X	
Diary collection																		X
Prescription			X		X			X			X			X				
Medication review		X	X	X	X	X	X	X	X	X	X	X	X	X	X		X	X
Return spare meds					X			X			X			X			X	

* If participants consent to take part in the PD STAT genetics sub-study, a blood sample for DNA will be taken at either the 12, 18, 24 or 26 month clinic visit.

Appendix 4: Statistical considerations

Figure A: Expected mean changes in MDS-UPDRS Part III from baseline to 24 months by group

μ_s is expected mean MDS-UPDRS part III change from baseline to 24 months for the simvastatin group
μ_p is the expected mean MDS-UPDRS part III change from baseline to 24 months for the placebo group

H_0: simvastatin is not futile

H_0: $\mu_s - \mu_p \leq -3$

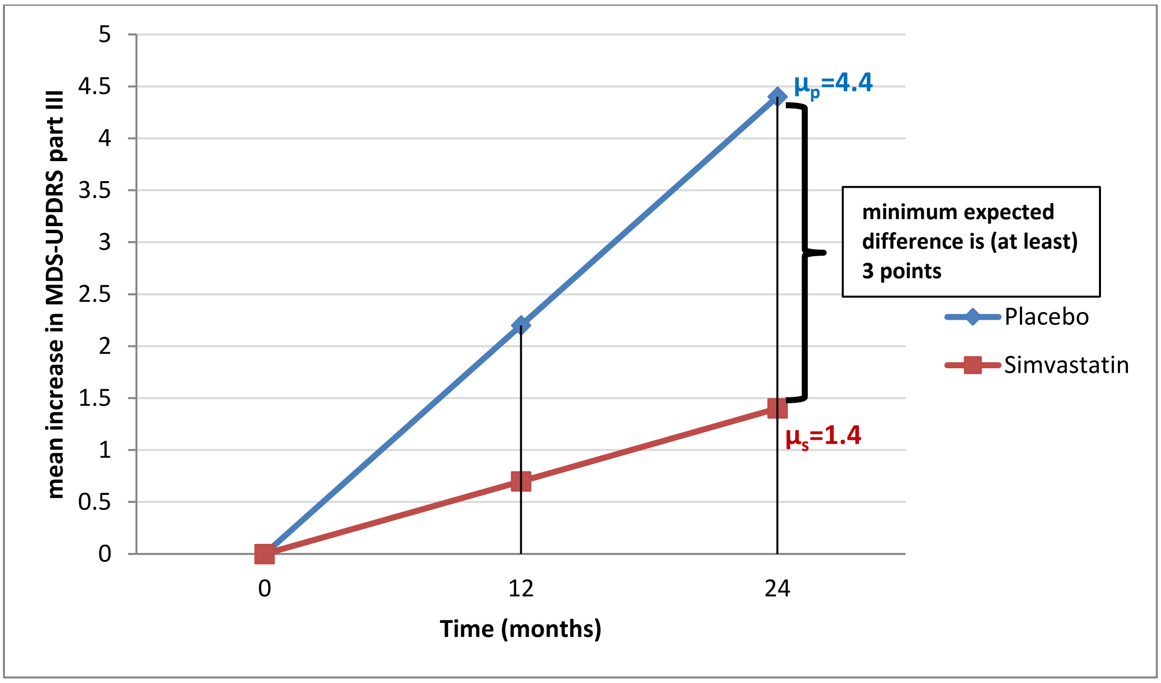

Figure B: Possible scenarios for observed differences between groups in changes in MDS-UPDRS part III

μ_s is expected mean MDS-UPDRS part III change from baseline to 24 months for the simvastatin group
μ_p is the expected mean MDS-UPDRS part III change from baseline to 24 months for the placebo group

H_0: simvastatin is not futile

H_0: $\mu_s - \mu_p \leq -3$

Possible Scenarios for Observed Differences in Changes

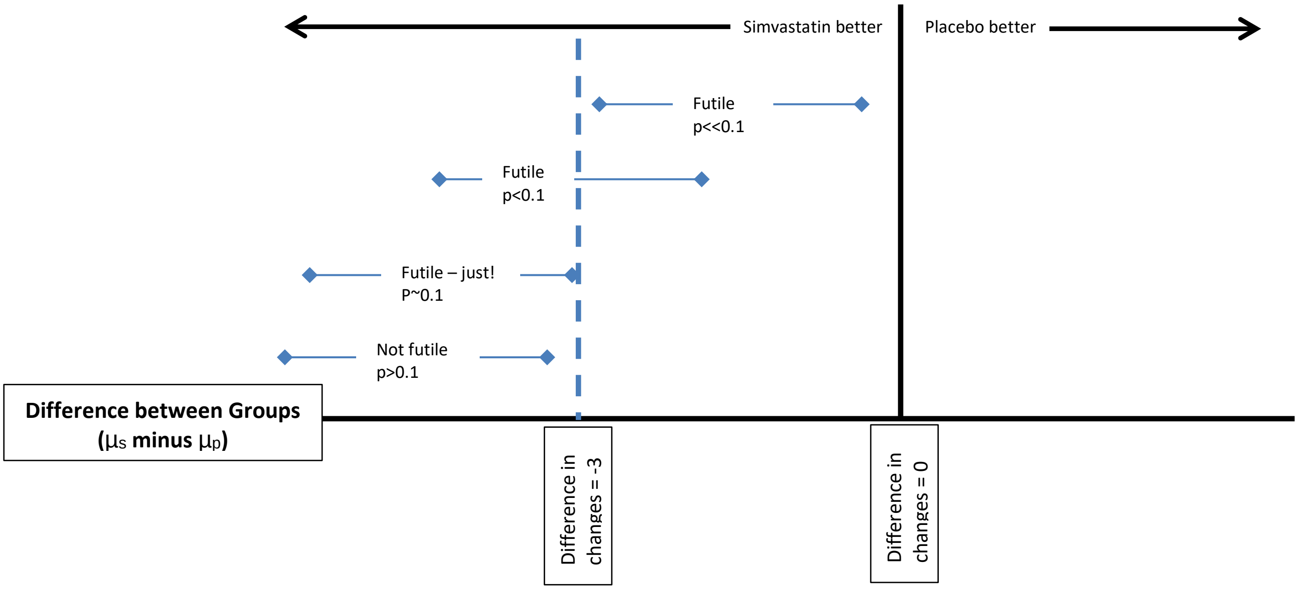

- Dashed blue line is pre-specified futility boundary of -3
- Calculate the adjusted mean increases from the regression model
- Increase in score is "bad"; so if take μ_s minus μ_p expect difference to be negative
- Hypothesis is that μ_s is 'better' i.e. smaller than μ_p by at least 3 units
- So if take μ_s minus μ_p expect difference to be -3 or lower
- Look at upper bound of two-sided 80% confidence interval (equivalent to one-sided 90% confidence interval)
- If upper bound is lower than -3, cannot reject null hypothesis of simvastatin not futile, so carry on
- If lower bound is above -3, reject null hypothesis of simvastatin not futile and stop

Appendix 5: Electromagnetic Sensor (EMS) Measurement

This measurement will be completed in addition to the MDS-UPDRS motor assessments completed at the 12 month and 24 month follow up visits, in seven PD STAT recruiting centres. If the measure is not completed at 24 months, participants will be invited to complete this at the 26 month visit.

The primary outcome measure for the main PD STAT study (MDS-UPDRS Part III, OFF) and timed motor tests (OFF) should ideally be completed before the additional EMS measurements are collected in the OFF state. The assessments required for the PD STAT study in the ON state should ideally be collected before the additional EMS measurements are collected in the ON state.

Participants will wear the electromagnetic (EM) sensors (Polhemus Inc.) on the index finger and thumb when they perform the assessments (see Figure 2). The EM sensors are commercially available and carry a CE mark for conventional use.

Assessors will be prompted to enter data into the tablet computer, including the following:

- Participant study number
- Whether it is the 12 or 24-month visit
- Dominant hand for writing
- Current on/off status

The tablet-computer will prompt the assessor to attach the sensors to the patient's right hand and then guide them through each of the 4 MDS-UPDRS items detailed below. The laptop will then prompt the assessor to attach the sensors to the patient's LEFT hand and then guide them through each of the 4 MDS-UPDRS items again.

MDS-UPDRS tremor items:

1) Hands at rest (with provocation task e.g. serial sevens) – 30 seconds

MDS -UPDRS bradykinesia items:

2) Finger tapping – 10 repetitions
3) Hand movements – 10 repetitions
4) Pronation/Supination – 10 repetitions

It is estimated that it will take approximately 5-7 minutes to complete the EMS kinematic assessments.

Figure 2 : The Patriot electromagnetic sensors (Polhemus Inc.)

The Patriot electromagnetic sensors (Polhemus Inc.) are attached to the thumb and index finger, as shown overleaf.

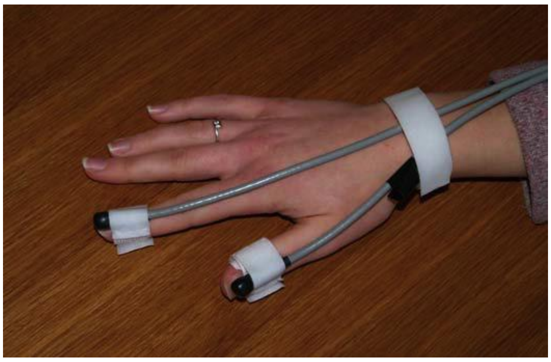

Data Management

Data resulting from the EMS assessments will be initially stored on the computer-tablet. At regular intervals, the data will be encrypted and backed-up to cloud-based storage (Google Drive, as recommended by the University of York as it has agreements in place with Google ensuring the security and physical location of the data).

Once received, the data will be decrypted by the team at York University and kept on password-protected secure systems within the University of York's firewall. Only authorised members of the research team will have access to the data for processing and analysis.

Statistical Analysis of Electromagnetic Sensor Measurement

The proposed analyses will be conducted by the team at York University and will be concerned only with the properties of the EMS assessment compared to those of other measures in the trial, and will not use the main trial's futility analysis approach. Analyses will use data for the centres where the EMS assessment is used and for participants for whom both variables being compared are available only.

The effect sizes for the EMS measure and the trial primary outcome, MDS-UPDRS part III (OFF) score will be estimated. Analyses will be done both for the final score at 24 (or 26) months and for the final score adjusted for covariates as described in the trial protocol (version 4.4, section 21.2). The effect sizes will be presented in standardised form as a multiple of the standard deviation of the baseline scores. The difference between the effect sizes for the EMS measure and MDS-UPDRS part III (OFF) score will be used as an estimate of the superiority (or otherwise) of the finger tapping measure. These analyses will be our primary analyses if simvastatin is shown to be non-futile.

The standard errors of the standardised treatment effect will be estimated in standard deviation units, and will be compared between the EMS measure and MDS-UPDRS part III (OFF) score. The superior outcome measure should have a smaller standard error for the effect. This will be done for the final measure at 24 (or 26) months and for the final measure adjusted for covariates as described in the protocol (version 4.4, section 21.2). These analyses will be our primary analyses if simvastatin is shown to be futile.

The observations of the finger tapping measure and MDS-UPDRS part III (OFF) score made at 12 and 24 months will be used to fit individual regression slopes over time, thus using both observations, and these slopes will be used as an outcome measurement, comparing the standardised effect sizes and standard errors.

The analyses detailed above will be used for other measures of Parkinson's disease outcome used in the study, including: the MDS-UPDRS total score in the practically-defined ON state, individual item scores from MDS-UPDRS part III (OFF and ON), timed motor tests and the Addenbrooke's Cognitive Examination-III (ACE).

This is an exploratory sub study; data to enable us to carry out sample size calculations for these analyses is not available. This sub study should give us sufficient data to plan further studies to investigate the properties of the finger tapping measurement and, we hope, improve the future conduct of research in Parkinson's disease.
