# Supplementary material for: Evaluation of Simvastatin as a Disease-Modifying Treatment for Patients With Parkinson Disease: A Randomized Clinical Trial
Source: JAMA Neurol. 2022 Oct 31;79(12):1232–41. doi: 10.1001/jamaneurol.2022.3718 (PMC9623477; doi:10.1001/jamaneurol.2022.3718)
Supplement: Supplement 2. — Statistical Analysis Plan [file jamaneurol-e223718-s002.pdf]

Statistical Analysis Plan

PD STAT

Simvastatin as a neuroprotective treatment for moderate Parkinson's disease

Simvastatin as a neuroprotective treatment for Parkinson's disease: a double-blind, randomised, placebo controlled futility study in patients of moderate severity

CONTENTS

Administrative Information 4

Abbreviations 9

1 Introduction 10

1.1 Background and Rationale for the Trial 10

1.2 Purpose of statistical analysis plan 10

2 Trial Objectives and Outcome Measures 10

2.1 Primary Objective 10

2.1.1 Primary Outcome Measures 11

2.2 Secondary Objectives 11

2.2.1 Secondary Outcome Measures 11

2.3 Practically–Defined OFF State 12

3 Trial Design 13

3.1 General Design 13

3.1.1 COVID-19 Adaptions 13

3.2 Blinding 13

3.3 Analysis Populations 13

3.3.1 COVID-19 Analysis Populations 14

3.4 Inclusion and Exclusion Criteria 14

3.4.1 Inclusion Criteria 14

3.4.2 Exclusion Criteria 14

4 Statistical Principles 15

4.1 Randomisation, Allocation Concealment and Stratification 15

4.2 Sample Size Calculation 15

4.3 Statistical Significance Levels 17

4.4 Compliance and Protocol Violations 17

4.4.1 Compliance to Allocated Treatment 17

4.4.2 Protocol Violation or Deviation 17

4.5 Timing of Collection of Outcome Measures 19

4.6 Derived Variables 19

4.7 Interim Analysis 20

4.8 Time points of Statistical Analysis 20

4.9 Data Sources and Data Quality 20

4.10 Missing Data 21
4.10.1 Missing Primary Outcome Data 21
4.10.2 Other Missing Data 22
4.10.3 Missing Data as a Consequence of COVID-19 22
5 Study Population 22
5.1 Participants who discontinue, withdraw or are lost to follow-up 23
5.2 Baseline characteristics and demographics 23
6 Statistical Analyses 24
6.1 General Considerations 24
6.2 Adjustments 24
6.3 Statistical Software 24
6.4 Reporting Conventions 25
7 Analysis of the Primary Outcome 25
7.1 Outcome Variable 25
7.2 Primary Analysis: Futility Analysis 25
7.2.1 Interpretation of the results of the primary analysis 25
7.3 Sensitivity Analyses of the Primary Outcome 27
7.3.1 Non-Compliance Sensitivity Analysis 27
7.3.2 Location of Face-to-Face Visit Sensitivity Analysis 29
7.3.3 COVID-19 Sensitivity Analysis 29
7.4 Secondary Analyses of the Primary Outcome 29
7.4.1 Repeated Measure Model 29
7.4.2 Disease-Modifying Analysis 30
7.5 Superiority Analysis 31
8 Analysis of Secondary Outcomes 32
8.1 Secondary Outcome Variables 32
8.2 Secondary Outcomes Analysis 33
8.2.1 COVID-19 Sensitivity Analyses of Secondary Outcomes 34
9 Safety Data 34
10 References 36
Appendix 39
A. Additional Information for Outcome Measures 39
B. Examples of Figures and Tables for Reporting Study Results 42
C. Superiority Analysis Correspondence 46

ADMINISTRATIVE INFORMATION

Short Title of Trial	Simvastatin as a neuroprotective treatment for moderate Parkinson's disease
Full Title of Trial	Simvastatin as a neuroprotective treatment for Parkinson's disease: a double-blind, randomised, placebo controlled futility study in patients of moderate severity
Trial Registration Numbers	EudraCT number: 2015-000148-40<br>ISRCTN16108482<br>REC Reference: 15/NE/0324
Protocol Version	4.5 (2nd February 2019)
SAP Version	2.1 (24th August 2020)
SAP Revisions	**Revisions 15th July 2020 (Statisticians' blinding maintained)**<br>Following final review of paper-based CRFs during the data cleaning process, a small proportion of participants from multiple sites were identified where the assessor had annotated the CRF to include separate left hand and right hand postural tremor scores (see **section 4.10.1** on missing an item from question 15 of the MDS-UPDRS part III). Prior to database lock, the trial database was updated to include these additional data. After discussion with the PD STAT Trial Management Group (13/07/2020), it was agreed that the calculation of the primary outcome should utilise all available data.<br>Therefore, **section 4.10.1** is updated as follows:<br>*For a small proportion of participants, the assessor manually annotated the case report forms and included postural tremor scores for the left and right hand. In these instances, the scores for both left and right hands will be used as part of the calculation of the primary outcome. However, if a participant only has one postural tremor score, the second postural tremor score will be treated as missing data (not at random).*<br>*For a maximum of three missing items, imputation of the MDS-UPDRS part III total score will be undertaken according to the following equation……*<br>*…… For the purposes of pre-defined sensitivity analyses of the primary outcome (outlined in section 7.3), the requirement of no more than three missing items will be relaxed.*

	Following a detailed examination of the MDS-UPDRS part IV during final data cleaning, it was identified that for questions 1, 3 and 6 assessors had not always completed the calculations required to determine the item score. After discussion with the Trial Management Group (13/07/2020), it was decided missing scores will be calculated by the trial statisticians after database export, if all the necessary data is available.<br><br>Therefore **Table 5 of Appendix A** was amended to include:<br><br>*MDS-UPDRS part IV*: *For questions 1, 3 and 6, the score is based on the percentage reported in part C of each question, which is calculated by dividing the number of hours stated in part B by the number of hours stated in part A and multiplying by 100. For each of questions 1, 3 and 6, the question score is then:*<br>• *0 if part B = 0*<br>• *1 if part C ≤ 25%*<br>• *2 if part C is 26-50%*<br>• *3 if part C is 51-75%*<br>• *4 if part C > 75%.*<br>*In instances where the ordinal score is missing but part C (the percentage) is available, the score will be determined based on the above thresholds of the reported part C. If the ordinal score and part C are missing but part A and B are available, part C (the percentage) will be calculated and the ordinal score determined by the above defined thresholds.*<br><br>**Revisions 15th July 2020 (Statisticians' blinding maintained)**<br><br>It was identified that Equation 4 and Equation 5 in **section 7.4** were missing the participant random effect. This had now been included as the term $U_{i,j}$ and the definition:<br><br>• $U_{i,j} \sim N(0, \sigma_u^2)$ *is the random effect for participant j in cluster i, independent of Z_i*<br><br>added to the list of definitions following Equation 4.

Revisions 27th July 2020 (Statisticians' blinding maintained)

In **section 7.4.2** of the SAP v1.1 (15/07/2020), the disease-modifying model of MDS-UPDRS part III (OFF) total score observed at baseline, 12, 24 and 26 months erroneously included an adjustment for the baseline MDS-UPDRS part III (OFF) total score. As part of the dependent variable, this should not also be included as an independent variable in the repeated measures or disease modifying models. Therefore, the text *"which is instead adjusted for as a fixed effect within θ",* has been deleted.

Following a review of the Adverse Events (AE) data, it was identified that the information required to determine whether an IMP discontinuation was due to either an AE or SAE had not been obtained. Therefore, instead of reporting the number of withdrawals and discontinuations due to AEs, summary statistics of AEs of all participants who discontinue or withdraw will be reported and **section 9** has been updated to:

The number and percentage of adverse events (AEs) will be reported by participant's completion status (i.e. completed IMP, discontinued IMP and withdrew) and by treatment taken (i.e. placebo, 40mg only or 80mg).

Revision 7th August 2020 (Statisticians' blinding maintained)

At the time of finalising the SAP v1.1 (15/07/2020), it was assumed that all visits undertaken after the introduction of government social distancing measures on 23rd March 2020 would be conducted remotely. As remote visits had not been an option prior to the COVID-19 lockdown, the presence of a remote visit was planned to be used as a proxy/indicator for an outcome measured during COVID-19 lockdown. However, following data export, it became apparent that one participant visit was completed face-to-face shortly after this date, on 25th March 2020. As a result, it was necessary to implement the following revisions to clarify that all outcome collection visits completed after 23rd March 2020 will be considered to be "COVID-19 affected" visits:

Section 3.3.1

> *The non-COVID-19 population will consist of participants whose data collection visits were completed on or prior to the introduction of government social distancing measures on 23rd March 2020.*

<table>
<tr><td></td><td>

Section 7.4.2.1

…… will include an indicator variable that identifies whether the visit was completed during COVID-19 lockdown. The estimated coefficient and associated 95% confidence interval of the COVID-19 indicator variable will be used to understand the effect of COVID-19 on the disease-modifying analysis.

Section 8.2.1

…… provided valid 24-month data during COVID-19 lockdown will be undertaken. Specifically, the analyses of each of the secondary outcomes will be repeated excluding participants who provided 24-month outcome data during COVID-19 lockdown when social distancing measures were in place (i.e. the non-COVID-19 population).

Revision 24th August 2020 (Group allocations revealed)

At the TMG meeting on 21st August 2020, it was discussed that missing items within the ACE-III were currently being set to zero. This could potentially lead to miss-assigning a participant a low ACE-III total score due to physical impairment rather than cognitive impairment. The decision was made to set the total ACE-III total score to missing for any participant missing one or more ACE-III items.

Therefore, **Table 5 in Appendix A** was amended to:

ACE-III	*Participants with any missing items set total score to missing (approach 3, Lees et.al [41])*

</td></tr>
<tr><td>SAP Clarifications</td><td>

(i) To maximise all available data, the disease-modifying analysis will use the compliance definition exactly as specified in **section 4.4.2(i)**, such that the ±2-week visit window only applies to the 24-month follow-up visit.

(ii) In **section 6.2** the adjustment for the modified Hoehn and Yahr score will be conducted according to the cut-off used to stratify the randomisation procedure (i.e. ≤2.0 versus 2.5-3.0).

(iii) For all 24-month group comparisons in which the model adjusts only for baseline (as per **section 6.2**), random effects for centre will **not** be included.

</td></tr>
</table>

	(iv) Analysis of the BTT in **section 8** should only use the first measurement of frequency of taps in 30 seconds for each hand at each visit, as an error within the BTT website resulted in multiple uploads of the same data or the participants repeating the tests as the website indicated the initial test had not been successful.<br><br>(v) The pre-specified COVID-19 sensitivity analyses of the secondary outcomes, as outlined in **section 8.2.1**, pertain only to the analysis models in which the 24-months outcomes are treated as the dependent variables (i.e. there is no planned COVID-19 sensitivity analyses of the repeated measures models). Given no data collection visits at baseline or 12-month follow-up were undertaken during the COVID-19 lockdown period, COVID-19 sensitivity analyses of the repeated measures models are not considered necessary.<br><br>(vi) Cholesterol levels that exceed 10mmol/L will be excluded from analyses, as these are considered clinically spurious values.

	Name	Signature	Date
Statistical Analysis Plan Authored by:	Trial Statistician: Kara N. Stevens		12/08/2020
	Trial Statistician: Siobhan Creanor		12/08/2020
	Trial Statistician: Ben Jones		12/08/2020
Approved by:	Chief Investigator: Camille Carrol		12/08/2020
	Independent Statistician (Trial Steering Committee): Dr Obioha Ukoumunne		20/08/2020

ABBREVIATIONS

ACE-III	Addenbrooke's Cognitive Examination-III
ADL	Activities of Daily Living
AE	Adverse Event
ALT	Alanine transaminase
AST	Aspartate transaminase
CI	Confidence interval
CK	Creatine kinase
CRF	Case Report Form
CTU	Clinical Trials Unit
CVD	Cerebrovascular Disease
DMC	Data Monitoring Committee
EDTA	Ethylenediaminetetraacetic acid
eGRF	Estimated Glomerular Filtration rate
EQ-5D-5L	EuroQoL 5D-5L health status questionnaire
HDL	High density lipoprotein
LED	Levodopa-equivalent dose
KPPS	King's PD pain scale
MADRS	Montgomery and Asberg Depression Rating Scale
MDS-UPDRS	Movement Disorder Society Unified Parkinson's disease Rating Scale
MoCA	Montreal Cognitive Assessment
NMSS	Non-motor symptoms scale
PD	Parkinson's disease
PDQ-39	Parkinson's disease Questionnaire
PenCTU	Peninsula Clinical Trials Unit
SAE	Serious Adverse Event
SAP	Statistical Analysis Plan
SOP	Standard Operating Procedure
SUSAR	Suspected Unexpected Serious Adverse Reaction
TMG	Trial Management Group
TSC	Trial Steering Committee
U&Es	Urea and electrolytes
10MWT	10 metre walk test

1 INTRODUCTION

1.1 BACKGROUND AND RATIONALE FOR THE TRIAL

The full background and rationale for the trial can be found in the PD STAT protocol[1]. In summary, Parkinson's disease (PD) is a progressive neurodegenerative condition affecting more than 127,000 people in the UK, with a further 10,000 individuals being diagnosed with the condition each year. No drug has been shown to slow or reverse the neurodegenerative process of PD. All currently licensed therapies act as symptom-relieving agents but have a limited lifespan of effectiveness because of continued neuronal loss. Epidemiological studies support a potential neuroprotective role for statins in PD, as statin use has been associated with a lower incidence rate of PD[2 3]. Various studies have suggested an association between the cholesterol lowering drug, Simvastatin, and a reduction in the rate of brain atrophy in secondary progressive multiple sclerosis[4].

The purpose of this futility study is to determine whether Simvastatin, a widely used cholesterol-lowering drug (statin), has potential to reduce the rate of neurodegenerative decline in patients with PD. The study is part of the Linked Clinical Trials initiative coordinated by The Cure Parkinson's Trust[5].

1.2 PURPOSE OF STATISTICAL ANALYSIS PLAN

The study protocol includes an outline of the statistical methods to be employed in the analysis of the trial data. The purpose of the Statistical Analysis Plan (SAP) is to provide full details of the planned statistical methods to be used in the primary report of the trial results.

The SAP has been drafted following published SAP Guidelines[6], also taking cognisance of the CONSORT for parallel group trials[7], the extension for reporting patient-reported outcomes[8] and Harms[9]. However, it is worth noting that, as this is a futility trial, there will be some differences in the analysis of the outcome measures and presentation of the results compared to a typical definitive randomised trial.

The electromagnetic sensor measurement and genetic sub-studies will be analysed separately by collaborators on the PD-STAT project. The statistical analyses outlined in this SAP do not pertain to data gathered from either of these sub-studies.

2 TRIAL OBJECTIVES AND OUTCOME MEASURES

The main objective of this trial was to determine the futility of Simvastatin in reducing neurodegenerative decline in patients with PD. The outcome measures will be recorded at baseline, 12, 24 and 26 months.

2.1 PRIMARY OBJECTIVE

To determine whether Simvastatin is clearly ineffective (futile) in preventing the clinical decline of PD, as measured by the Movement Disorder Society Unified Parkinson's Disease Rating Scale (MDS-UPDRS)[10] motor score.

2.1.1 Primary Outcome Measures

The primary outcome is change in MDS-UPDRS part III motor subscale score in the OFF state over 24 months. MDS-UPDRS part III score consists of 33 items, where each item is scored between 0 (normal) and 4 (severe). The OFF state is defined as the absence of the participant's regular PD medication so that the severity of the underlying disease is evident; more details can be found in section 2.3.

2.2 SECONDARY OBJECTIVES

The secondary objectives are to:

i. Confirm the safety and tolerability of Simvastatin in patients with PD.
ii. Distinguish symptomatic effects of Simvastatin from disease modifying effects.
iii. Evaluate the impact of Simvastatin on:
   a) activities of daily living (ADL)
   b) timed motor tests
   c) cognitive ability
   d) mood
   e) behaviour
   f) non-motor symptoms
   g) quality of life.

2.2.1 Secondary Outcome Measures

The secondary outcome measures collected at baseline, 12, 24 and 26 months are:

i. *MDS-UPDRS total score* in the practically defined ON state – the total score consists of 65 items, where each item is scored between 0 (normal) and 4 (severe).

ii. *MDS-UPDRS part II subscale score* in the practically defined ON state – the part II score consist of 13 items, where each item is scored between 0 (normal) and 4 (severe).

iii. Timed motor tests:
   a) *brain tap test*[11] in the OFF state, where the measurement used is the number of key taps in 30 seconds,
   b) *10 metre walk test (10MWT)*[12] in the OFF state, which will consist of two components: number of 10MWTs the participant was able to complete out of three, and the mean time taken to successfully complete 10MWT.

iv. *Montgomery and Asberg Depression Rating Scale (MADRS)*[13 14] – a 10-item physician rated depression severity scale where each item is scored from 0 (best) to 6 (worst).

v. *Addenbrooke's Cognitive Examination-III (ACE-III)*[15] – total score which includes five subdomains: attention (score: 0-18), memory (score: 0-26), fluency (score: 0-14), language (score: 0-26) and visual spatial (score: 0-16).

vi. *Non-Motor Symptom scale (NMSS)*[16] – total score which comprises of 30 item rater-based instrument, specifically designed for the comprehensive assessment of non-motor symptoms in patients with PD. It contains nine dimensions: cardiovascular (2 items), sleep/fatigue (4 items), mood/cognition (6 items), perceptual problems (3 items), attention/memory (3 items), gastrointestinal (3 items), urinary (3 items), sexual function

(2 items) and miscellaneous (4 items). Each item is scored for severity (0-3) and frequency (0-4).

vii. *Parkinson's disease Questionnaire (PDQ-39)*[17] *total score* – this questionnaire assesses how often people affected by Parkinson's experience difficulties. It consists of 39 items across eight discrete dimensions: mobility (10 items), activities of daily living (6 items), emotional well-being (6 items), stigma (4 items), social support (3 items), cognitions (4 items), communications (3 items) and bodily discomfort (3 items). Each item is scored between 0 (never) and 4 (always).

viii. *Levodopa-equivalent dose (LED)* – this measure allows for comparison of different types of PD medication by calculating the equivalent dose in Levodopa.

ix. *Cholesterol levels* – this will include measurements of total, HDL, total/HDL ratio cholesterol levels.

x. *King's PD pain scale (KPPS)*[18] *total score* - a PD specific pain score consisting of 14 items within seven domains: musculoskeletal pain (1 item), chronic pain (2 items), fluctuation related pain (3 items), nocturnal pain (2 items), orofacial pain (3 items), discoloration and Oedema/swelling (2 items) and radicular pain (1 item). Each item is scored for severity (0-3) and frequency (0-4).

xi. *EuroQoL 5D-5L health status questionnaire (EQ-5D-5L)*[19].

At 24 months incidence of diabetes mellitus, determined by a glycated haemoglobin (HbA1c) level ≥ 6.5% (48mmol/mol) (WHO 2011), will also be recorded.

Every two months the safety and tolerability data will be gathered, using adverse events reporting, via telephone contacts (2 weeks, 2, 4, 8, 10, 14, 16, 20 and 22 months post-baseline) or during a follow-up visit (baseline and at 1, 6, 12, 24 and 26 months post-baseline). In addition, the number of capsules returned will be recorded every 6 months, in order to aid the determination of tolerability and compliance.

2.3 PRACTICALLY–DEFINED OFF STATE

The MDS-UPDRS part III and the timed motor tests at baseline, 12, 24 and 26 months need to be conducted in the absence of the participant's regular PD medication (known as the OFF state) so that the severity of the underlying disease is evident.

To minimise the time participants are in the OFF state and allow for withdrawal of participant's PD medication, study visits should be held in the morning and participants should attend having omitted their prescribed short acting PD medications (e.g. Levodopa or Ropinirole Immediate release) from 1800 hours on the day before the visit. Long acting agents (e.g. Ropinirole XL) should be omitted the day before the visit and on the day of the visit itself.

To reduce any physical discomfort of stopping PD medications and to facilitate attendance at clinic, the local research team should make arrangements to provide the participant with a prescription for relevant supportive medications (e.g. Zopiclone/Zolpidem for night sedation, paracetamol for pain relief and/or diazepam for treatment of dystonia) as necessary.

Participants may also be prescribed dispersible Madopar as a rescue medication to be taken in the event of severe difficulty with wearing-off symptoms. If a participant has taken dispersible Madopar after omitting his/her regular PD medication prior to a study visit, the study visit should be rescheduled. If further attempts at attending in the OFF state fail, the participant should be withdrawn from the study.

3 Trial Design

3.1 General Design

This is a double-blind, randomised, placebo-controlled, multi-centre parallel group futility trial in patients with PD of moderate severity. The plan is to recruit and randomise 198 patients in a 1:1 ratio to receive either oral Simvastatin or matched placebo for 24 months. A one month low dose phase of 40mg daily will be followed by a 23 month high dose phase of 80mg daily and a final two month phase off trial medication. Participants will be followed up at 1 month, 6, 12, 18, 24 and 26 months (±2 weeks) post-baseline. Visits to obtain outcome measures are undertaken face-to-face, and can be either in an outpatient clinic or at home.

3.1.1 COVID-19 Adaptions

Following the start of the COVID-19 outbreak in early 2020, government guidelines led to a restriction on face-to-face trial visits. As a result, it will be necessary to conduct a small number of primary endpoint (24 month) visits remotely (e.g. using Skype), as well as a number of 26-month visits.

3.2 Blinding

Participants will be blind to treatment allocation throughout the trial, as will the trial management team, investigator site teams and site pharmacy staff. The primary statistical analyses of the primary outcome will be undertaken blinded to the allocated group.

3.3 Analysis Populations

We will base the primary analysis of the primary outcome on a modified intention-to-treat (mITT) principle. The mITT evaluable sample for the primary analysis will include all participants who are randomised, provide baseline outcome data, commence to the higher dose phase of the study and provide 24-month outcome data. Imputation will only be performed for missing questionnaire items that fulfil pre-specified criteria as outlined in section 4.10.

To investigate any effect of compliance with taking study medication, if ≥ 20% of participants in the active Simvastatin group are identified as non-compliers (according to each of the definitions in section 4.4.2), we will undertake additional sensitivity analyses of the primary outcome (further details in section 7.3.1). This population will consist of all participants with baseline and 24-month primary outcome data.

The safety population will consist of all participants who have at least one dose of allocated trial treatment.

A washout period has been included in the study design to explore the potential disease-modifying effect of Simvastatin. Trial treatment will be discontinued at 24 months and a follow-up visit is conducted two months later at 26 months post-baseline. All participants still on trial treatment at the higher dose of 80mg at 24 months with valid baseline, 24 and 26 months data will be included in the disease-modifying analysis.

3.3.1 COVID-19 Analysis Populations

To facilitate sensitivity analyses to explore any effect of the COVID-19 outbreak, two further populations are defined. The non-COVID-19 population will consist of participants whose data collection visits were completed on or prior to the introduction of government social distancing measures on 23rd March 2020. Note different numbers of participants will be included in this population when considering the 24 month and 26 month follow-up visits, due to the timing of the COVID-19 restrictions.

The COVID-19 population will consist of participants who had one or more follow-up visits completed remotely. Again, the numbers of participants included in this population will differ for the 24 month and 26 month end-points.

3.4 Inclusion and Exclusion Criteria

3.4.1 Inclusion Criteria

Potential participants must satisfy the following criteria to be enrolled in the study:

- Diagnosis of idiopathic PD
- Modified Hoehn and Yahr stage ≤ 3.0 in the ON medication state
- Age 40-90 years
- On dopaminergic treatment with wearing-off phenomenon
- Able to comply with study protocol and willing to attend necessary study visits

3.4.2 Exclusion Criteria

Potential participants meeting any of the following criteria will be excluded from study participation:

- Diagnosis, or suspicion, of other cause for parkinsonism
- Known abnormality on CT or MRI brain imaging considered to be causing symptoms or signs of neurological dysfunction, or considered likely to compromise compliance with study protocol
- Concurrent dementia defined by MoCA score < 21
- Concurrent severe depression defined by MADRS score > 31
- Prior intracerebral surgical intervention for PD including deep brain stimulation, lesional surgery, growth factor administration, gene therapy or cell transplantation
- Already actively participating in a research study that might conflict with this trial
- Prior or current use of statins as a lipid lowering therapy
- Intolerance to statins
- Untreated hypothyroidism

- End stage renal disease (creatinine clearance < 30 mL/min) or history of severe cardiac disease (angina, myocardial infarction or cardiac surgery in preceding two years)
- Estimated Glomerular Filtration rate (eGFR) < 30 mL/min
- History of alcoholism or liver impairment
- Creatine kinase (CK) > 1.1 x upper limit of normal (ULN)
- Aspartate transaminase (AST) or alanine transaminase (ALT) > 1.1 x ULN
- Females who are pregnant or breast feeding or of child-bearing potential and unwilling to use appropriate contraception methods whilst on trial treatment
- Currently taking any medication contraindicated with Simvastatin use (Appendix 2 of the protocol v4.5)
- Any requirement for statin use
- Regular participation in endurance or high-impact sports
- Unable to abstain from consumption of grapefruit-based products

4 STATISTICAL PRINCIPLES

University of Plymouth medical statisticians (Peninsula Clinical Trials Unit) will carry out statistical analysis at the end of the study (following last participant's last visit and database lock).

4.1 RANDOMISATION, ALLOCATION CONCEALMENT AND STRATIFICATION

The Peninsula Clinical Trials Unit (PenCTU) will provide a 24-hour web-based, 1:1 randomisation system using random permutated blocks, stratified by site and modified Hoehn & Yahr stage (≤ 2.0 or 2.5 - 3.0), prepared by a statistician independent of the trial team. Randomisation takes place between screening and baseline visit, after confirmation of eligibility. Completion of the randomisation process will generate the participant's study number, initials and allocated treatment bottle number for the baseline visit, which needs to be printed and submitted to the hospital pharmacy.

Only members of the PenCTU programming team are able to access participants' treatment allocation, except in the event of Suspected Unexpected Serious Adverse Reaction (SUSAR).

4.2 SAMPLE SIZE CALCULATION

In futility studies, the direction of the hypotheses is different from that in traditional phase III efficacy or effectiveness trials. The study sample size was based on testing the *null hypothesis that Simvastatin is not futile*, in terms of the primary outcome (change in MDS-UPDRS part III motor subscale score in the OFF state at 24 months). If at the end of the study there is evidence to reject the null hypothesis, then it is unlikely that Simvastatin will be considered for a phase III study, although the final decision on planning for progression to a phase III study will be made in discussion with the trial oversight committees and other key stakeholders.

The minimum clinically important difference (MCID) in UPDRS motor score has been estimated to be between 2.3 and 2.7 points[20]. The null hypothesis, H_0, in this futility study is that the mean change in MDS-UPDRS part III score between baseline and 24 months for the Simvastatin group is at least 3 points less (as higher MDS-UPDRS scores are worse) than the corresponding mean change in the placebo group. The alternative hypothesis, H_1, is that the mean change in MDS-UPDRS part III score for the Simvastatin group is not at least 3 points less than the corresponding mean change in the placebo group. Mathematically, this is written as

$$H_0: \mu_s - \mu_p \leq -3 \text{ versus } H_1: \mu_s - \mu_p > -3 \text{ ,}$$

where μ_s is the expected mean change in MDS-UPDRS part III score from baseline to 24 months for the Simvastatin group and μ_p is the expected mean change for the placebo group.

Due to this change in the hypotheses in comparison with conventional phase III studies, the type I and type II error probabilities are interpreted differently compared to the traditional efficacy/effectiveness studies:

- A type I error occurs when the active treatment (Simvastatin) is non-futile but is rejected following the futility study, leading to an effective treatment not being considered for a phase III study (i.e. an erroneous rejection of H_0); the probability of this occurring is denoted by α;
- A type II error occurs when the active treatment (Simvastatin) is futile but there is insufficient evidence to reject the null hypothesis of non-futility, and an ineffective treatment is considered for a phase III study (i.e. an erroneous failure to reject H_0); the probability of this occurring is denoted by β[21].

Hence α and β were chosen to reflect the futility design-based hypotheses, with the one-sided α set at 10% and β at 20% (i.e. 80% power).

Based on a recently reported study at the time of the design of this futility trial, the expected mean increase in MDS-UPDRS part III from baseline to 12 months in the placebo group is assumed to be 2.2 points, with corresponding standard deviation of 7.3 points[22]. Assuming that this increase in MDS-UPDRS part III is linear over time, gives an expected mean increase from baseline to 24 months of 4.4 points in the placebo group (i.e. μ_p = 4.4). Therefore, if we assume the MCID is -3, this implies the mean of the Simvastatin group, μ_s , is 1.4 points. Additionally, we assume a slightly inflated standard deviation over this period of 7.5 points.

The sample size calculation was based on a two-sample t-test with a 10% one-sided alpha. Following the previously listed assumptions, the number of participants needed to follow-up at 24 months (i.e. with primary outcome data) is 57 participants per group, to provide 80% power to reject the null hypothesis of non-futility and declare futility.

This sample size was inflated to allow for a proportion of participants allocated to the Simvastatin group to stop taking the trial medication during the initial 1-month low dose phase. Assuming that this proportion was 15%, the above sample size was inflated by $(1–0.15)^{-2}$, to give 79 participants per group[23]. Secondly, the sample size was adjusted to allow for a (non-differential) loss to follow-up rate of 20%[1]. Accordingly, the sample size was further inflated by a factor of $(1-0.2)^{-1}$, to give a sample size of 99 participants per group and a total recruitment target of 198 participants commencing the higher dose phase of the study.

4.3 Statistical Significance Levels

As this is a futility study, a one-sided test of the primary outcome is appropriate, which has been set at the 10%[21] significance level. All other outcome variables will assessed on a two-sided test at the 5% significance level with corresponding two-sided 95% confidence intervals.

4.4 Compliance and Protocol Violations

4.4.1 Compliance to Allocated Treatment

Compliance with treatment allocation is assessed throughout the study, at each visit (at 1, 6, 12, 18, 24 months post-baseline) and telephone contacts (at 2, 4, 8, 10, 14, 16 and 22 months post-baseline).

4.4.2 Protocol Violation or Deviation

Non-compliance may occur in some instances if participants do not complete their allocated treatment according to the study protocol, or if the follow-up visit was not within ±2 weeks of the scheduled date. This could occur because of medical issues such as fatigue, deterioration of health, acceleration of PD, difficulty in attending outpatient clinics in the OFF state, or participants may simply decide that they no longer want to complete the programme.

Within the PD STAT study protocol (section 17.9), there is allowance for study drug dose alterations, depending on whether the patient is at the initial low dose phase or high dose phase. If a participant discontinues treatment during the low dose phase, attempts will be made to recruit an additional participant. During the high dose phase, participants with unwanted symptoms/side effects may reduce to the 40mg dose and then may continue on the 40mg dose for the remainder of the trial or, at the discretion of the local investigator, such participants may later be re-challenged with the 80mg dose after resolution of the unwanted symptoms. Participants who are on the 80mg dose and subsequently fulfil the trial stopping criteria will be withdrawn from treatment but invited to continue with the study assessments.

Complete identification of compliance with the study protocol may not be possible. For example, there may be unreported instances where participants miss doses of their medication or medication containers are not returned. Compliance will be assessed based on a combination of each participant's self-reported compliance during telephone calls and capsule count, but this may not capture the participant's true compliance. Therefore, we will base compliance on the reported data, acknowledging it may have limitations. Figure 1 outlines the possible forms of non-compliance we will be able to identify through the data collected.

We have constructed two definitions of study compliance. These are:

(i) Participants are compliant only if they proceed to and maintain the 80mg dose and attend 24 month follow-up within their 2-week window of their due date, and non-compliant if they remain on the 40mg dose, discontinue trial treatment or attend the 24-month follow-up visit outside of the 2-week window.

(ii) Participants are compliant if they take and maintain 80mg or 40mg and attend the 24 month follow-up visit within the 2-week window of their due date, and non-compliant if they attend their 24-month follow-up outside of the 2-week window or discontinue trial treatment.

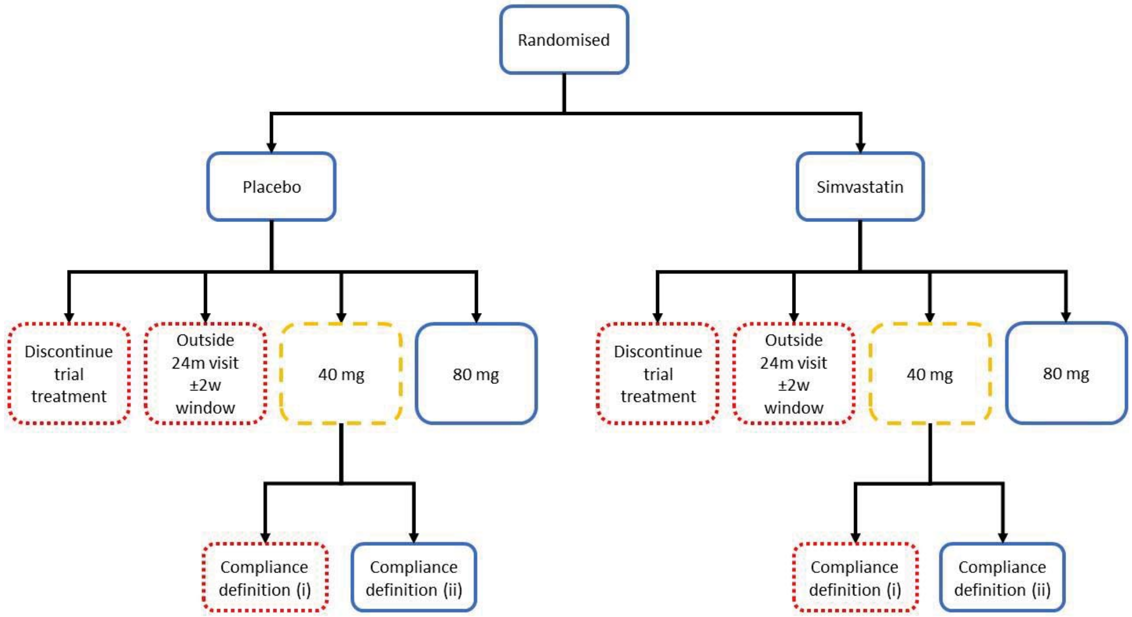

Figure 1: Possible scenarios for non-compliance during the PD Stat study. Where 24m = 24 months and 2w = 2 weeks. Blue solid line boxes denotes compliance with the study protocol; the yellow dashed boxes denotes participants who may be considered as compliers or non-compliers depending on whether definition (i) or (ii) for compliance is used; the red dotted line boxes represents non-compliance.

The number and proportions of participants classified as non-compliers, as defined above, will be summarised for each allocated group separately and overall, alongside details of the non-compliances. A formal complier-average causal effect (CACE) analysis will proceed if at least 20% of participants in the Simvastatin group are classified as non-compliers according, in turn, to each of the definitions above. Whilst non-compliance rates will be calculated and presented for participants allocated to the placebo group as well as participants allocated to the intervention group, their compliance is not relevant to the one-sided CACE analysis, in which non-compliance to the placebo/control does not imply receipt of the active intervention.

There are no planned protocol deviations. Any unintended protocol deviations will be documented and reported to the Chief Investigator and Sponsor. The number of deviations and percentage (number) of participants with protocol deviations will be reported.

4.5 TIMING OF COLLECTION OF OUTCOME MEASURES

Outcome measures are collected at baseline, 12 (±2 weeks), 24 (±2 weeks) and 26 (±2 weeks) months post-baseline. The primary endpoint is the 24-month review. Table 1 lists all the outcome measures, the ON or OFF state of participants when they were measured and the timing of these observations.

Variable	State*	Baseline	12 months	24 months	26 months
**MDS-UPDRS Part III (primary outcome)**	**OFF**	**X**	**X**	**X**	**X**
MDS-UPDRS Part II	ON	X	X	X	X
MDS-UPDRS Total	ON	X	X	X	X
Brain tap test	OFF	X	X	X	X
Walk Test (10MWT)	OFF		X	X	X
MADRS Total score	ON		X	X	X
ACE-III Total score	ON	X	X	X	X
NMSS Total score	ON	X	X	X	X
PDQ-39 Total score	ON	X	X	X	X
KPPS Total score	ON	X	X	X	X
EQ-5D-5L	ON	X	X	X	X
Total & HDL Cholesterol		X**	X	X	
LED		X	X	X	X
Diabetes				X	

* See section 2.3 for definition of ON/OFF states; ** Measurements taken at screening but will be referred to as baseline

Table 1: Table of outcome measures collected and time point

4.6 DERIVED VARIABLES

Some of the variables to be analysed will be derived from the raw collected data. Below is a list of these variables and the methods to be used to derive the variables for analysis:

- Age at baseline - calculated from date of birth and date of baseline visit.
- Number of years since PD diagnosis – calculated by subtracting age of PD diagnosis from age at baseline.
- BMI – calculated using participant's weight in kilograms divided by their height in metres (kg/m^2).
- MDS-UPDRS part III score – the sum of all 33 items within part III of MDS-UPDRS, giving a possible score between 0 and 132.
- MDS-UPDRS part II score – the sum of all 13 items within part II of MDS-UPDRS, giving a possible score between 0 and 52.
- MDS-UPDRS total score – the sum of all 65 items of the MDS-UPDRS, giving a possible score between 0 and 260.
- Brain tap test – mean frequency key taps of left and right hand.

- 10MWT – a binary outcome of whether participant was able to complete at least one 10MWT and mean time for participant to complete a 10MWT calculated from all successfully completed attempts.
- MADRS Total score – the sum of all 10 items, giving a total score between 0 and 60.
- ACE-III Total score – the score from each of the five subdomains, where the score for: attention is 0-18; memory is 0-26; fluency is 0-14; language is 0-26 and visual spatial is 0-16. The total score is then a value between 0 and 100.
- NMSS Total score – for each item, the severity is multiplied by frequency, resulting in an item sub-score of 0 to 12. Each item is then summed, giving a total score between 0 and 360.
- PDQ-39 Total score – the total scores from each of the eight dimensions are converted into a scale from 0 (best) to 100 (worst) by dividing by the maximum possible score of that dimension and then multiplying by 100. The total overall PDQ-39 score is then calculated by summing the eight dimension total scores and then dividing by 8.
- Total/HDL Cholesterol ratio – total cholesterol level divided by HDL.
- KPPS Total score – for each of the 14 items amongst the seven domains, the severity (scored as 0 to 3) is multiplied by the reported frequency (scored as 0 to 4), resulting in an item sub-score of 0 to 12. The 14 items are then summed, giving a total score between 0 and 168.
- EQ-5D-5L – the crosswalk[24] of the score to EQ-5D-3L will be calculated.
- LED – calculation of the LED will depend on the conversion factor, listed in Appendix A: Table 4, taken from Tomlinson, et al. [25] and Cervantes-Arriaga, et al. [26], where the daily dose in mg of the medication is multiplied by the conversion factor.

4.7 INTERIM ANALYSIS

There is no planned interim analysis in this study.

4.8 TIME POINTS OF STATISTICAL ANALYSIS

Statistical analysis will be performed after the final assessment of the last participant has been submitted and the trial database has been locked.

4.9 DATA SOURCES AND DATA QUALITY

The research nurse or other member of the research team will check completed case report forms (CRFs) for missing data or obvious errors before forms are sent to the CTU. Data will be monitored centrally for quality and completeness by the CTU and every effort will be made to recover data from incomplete forms where possible. The CTU data manager will oversee data tracking and data entry and initiate processes to resolve data queries where necessary. The CTU trial manager will devise a risk-based monitoring plan specific to the study, which will include both central monitoring strategies and study site visits as appropriate.

If there is evidence of fraudulent data, this will be considered a major non-compliance. Potential fraudulent data will be:

- Noted in the final report
- Summarised

Consideration will be given to excluding all data for any site where fraudulent data is suspected or detected.

4.10 Missing Data

Data for participants who miss follow-up visits will not be imputed. The timing and frequency of missing data will be summarised.

4.10.1 Missing Primary Outcome Data

Summary statistics of the number of missing items, and frequencies and percentages of participants with missing items of the MDS-UPDRS part III score will be reported.

Provided all missing items of MDS-UPDRS part III are missing at random, the total score for a given participant can be imputed if a maximum of seven of the 33 possible items are missing. Alternatively, if the same item is missing across all participants for one follow-up time-point, a maximum of three items can be missing to allow for valid imputation of the total score for each participant[27].

In PD STAT, one item of the MDS-UPDRS part III was erroneously omitted from case report forms at all time-points. Question 15 should consist of two items, a score for postural tremor for each of the left and right hands. However, only one item response could be recorded and the case report form did not specify, or request specification of, which hand. For a small proportion of participants, the assessor manually annotated the case report forms and included postural tremor scores for the left and right hand. In these instances, the scores for both left and right hands will be used as part of the calculation of the primary outcome. However, if a participant only has one postural tremor score, the second postural tremor score will be treated as missing data (not at random).

For a maximum of three missing items, imputation of the MDS-UPDRS part III total score will be undertaken according to the following equation

$$D = \frac{A \times B}{C}$$

Equation 1

where A is the sum of the items scored, B is the total number of items which should have been scored (for MDS-UPDRS part III B = 33), C is the number of items actually scored and D is the imputed score (i.e. total MDS-UPDRS part III score).

For the purposes of pre-defined sensitivity analyses of the primary outcome (outlined in section 7.3), the requirement of no more than three missing items will be relaxed.

4.10.2 Other Missing Data

If there are validated methods for imputing missing items in self-reported secondary outcomes, these will be used to impute total scores. Details of imputation methods to be used to handle missing data can be found in Appendix A: Table 5.

4.10.3 Missing Data as a Consequence of COVID-19

Although the majority of outcomes can be completed remotely, due to the nature of some of the tasks, a small number of items/measurements cannot be assessed during remote visits. For the MDS-UPDRS part III, question 3 (rigidity, consisting of 5 items) and question 12 (postural stability, consisting of 1 item) cannot be assessed remotely. This will affect calculation of the primary outcome, total MDS-UPDRS part III assessed in the OFF state, and corresponding secondary outcome assessed in the ON state.

In addition, the ACE-III cannot be completed remotely, whilst completion of 10MWT is dependent on the suitability of the participant’s house or outside space and completion of the BTT is dependent on a stable internet connection and the availability of a tablet or computer. Finally, outcomes derived from the blood test at 24 months will be missing for participants in the COVID-19 population.

5 STUDY POPULATION

Data from the screening process through to the completion of the trial will be recorded and presented in a CONSORT-style flow diagram (see Figure 4 in Appendix B). In particular, the following data will be provided:

- Number of participants screened for eligibility
- Number of participants ineligible*
- Number of participants eligible and asked to participate
- Number of participants who declined to participate*
- Number of participants consented to participate
- Number of participants withdrawn prior to randomisation*
- Number of participants randomised to each allocated group
- Number of participants who did not receive their allocated treatment*
- Number of participants who did receive their allocated treatment
- Number of participants who progressed to the higher dose
- Number of participants who did not progress to the higher dose*
- Number of participants who did not complete their allocated treatment*
- Number of participants who completed their allocated treatment
- Number of participants who completed each of the 12, 24 and 26 month follow-up visits
- Number of participants lost to follow up*
- Number of participants analysed

*Reasons will be provided where available.

5.1 Participants who discontinue, withdraw or are lost to follow-up

All participants will be encouraged to continue with study visits and assessments as per the study protocol even if trial treatment is discontinued prematurely. If a participant discontinues in the first 1-month low dose phase, attempts will be made to recruit additional participants to *supplement* participants who discontinue treatment during the first phase, even if they are continuing with the follow-up visits. The number of participants who are recruited due to an earlier participant discontinuing trial treatment during the 1-month low dose stage will be reported.

Participants who discontinue allocated treatment will be categorised into one of the following:

- Continue to consent for follow-up and data collection
- Consent to use pre-collected data only
- No further follow-up and withdraw consent to use data already collected

Reasons for withdrawal or loss to follow up will be summarised where possible, at each stage of the trial (withdrawal prior to randomisation, patients who did not receive their allocated treatment, non-completion of treatment, lost to follow-up).

5.2 Baseline characteristics and demographics

The demographic data will include:

- Age in years at baseline
- Gender
- Body Mass Index (BMI)
- Ethnic origin
- Smoking status
- Modified Hoehn & Yahr score
- Age in years of PD onset
- Number of years since PD diagnosis at baseline
- Diabetes status at screening
- QRISK® 2 score
- Montreal Cognitive Assessment Score (MoCA)
- Aspartate transaminase (AST)
- Alanine transaminase (ALT)
- Creatine kinase (CK)

The data will be summarised using descriptive statistics for all recruited participants. Summaries will be produced for participant's who only participate in the 1-month low dose phase of the study.

6 STATISTICAL ANALYSES

6.1 GENERAL CONSIDERATIONS

Descriptive statistics will be presented for all outcomes at all available time points. For continuous outcomes, summary statistics will be presented in the form of means (including mean difference between follow-up and baseline where appropriate) alongside standard deviations and ranges, or other suitable summary statistics such as medians and inter-quartile range, depending on the distributional properties of the variable. For categorical outcomes, summary information will be presented in the form of frequencies and percentages.

The primary analysis of the primary outcome will be presented with a one-sided p-value and two-sided 80% confidence interval (see 7.2.1 for further details). Analyses of the secondary outcomes will be presented with two-sided p-values and two-sided 95% confidence intervals wherever possible. If the evidence suggests modelling the raw measurements of the primary outcome is inappropriate, bootstrapped confidence intervals of the between-group differences will be presented. If the model assumptions for a secondary outcome are violated, an appropriate transformation of the outcome will be sought or alternatively the between-group difference will be presented alongside the bootstrapped confidence interval.

6.2 ADJUSTMENTS

The primary analysis of the primary and secondary outcomes will include fixed effects adjustments baseline outcome, gender, age at baseline and modified Hoehn and Yahr score[28]. Recruiting site will be included as a random effect in order to account for any potential unknown variation associated with each site.

In addition to the adjustments specified in the protocol, we will also include duration of PD, as recent evidence suggests that the rate of change in MDS-UPDRS differs according to disease duration[29]. If there is a suggestion from a visual inspection of any large difference in any of the other baseline characteristics/ demographics between allocated groups, sensitivity analysis will include additional adjustments for such a covariate.

For completeness and in line with EMA guidance[30], the results of models only including adjustment for baseline scores (where available) will also be presented.

6.3 STATISTICAL SOFTWARE

The data will be analysed using STATA (version 14 or above)[31] and supplemented with R[32] where necessary.

6.4 Reporting Conventions

P-values ≥ 0.001 will be reported to three decimal places; p-values less than 0.001 will be reported as "< 0.001". The mean, standard deviation, and any other statistics other than quantiles, will be reported to one decimal place greater than the original data. Quantiles, such as median, or minimum and maximum, will use the same number of decimal places as the original data. Estimated parameters not on the same scale as raw observations (e.g. regression coefficients) will be reported to three significant figures.

7 Analysis of the Primary Outcome

7.1 Outcome Variable

The primary outcome is the change in MDS-UPDRS part III in the OFF state between baseline and 24 months. The total score is calculated from the 33 items within the MDS-UPDRS part III, where each item is scored between 0 and 4.

7.2 Primary Analysis: Futility Analysis

The aim of the primary analysis is to determine whether Simvastatin is clearly ineffective (futile) in preventing the decline of PD. This will be achieved by a between-group comparison of change in MDS-UPDRS part III from baseline to 24 months, measured in the OFF state.

Specifically, a mixed-effects linear regression model will be fitted to the change in MDS-UPDRS part III scores between baseline and 24 months (y), for participants $j = 1, \dots, n_i$, from site $i = 1, \dots, 23$ of the form:

$$y_{i,j} = \delta_s x_{1,i,j} + \boldsymbol{\theta}\boldsymbol{x}_{i,j} + Z_i + \varepsilon_{i,j}$$

Equation 2

where x_1 is an indicator variable for allocated group and δ_s is the mean difference between treatment groups. The vector $\boldsymbol{x}_{i,j}$ represents the row of the design matrix for participant j from site i, where the first element is 1 and the subsequent elements are the fixed effects covariates defined in section 6.2, with corresponding coefficient vector $\boldsymbol{\theta}$. The random effects (recruiting sites) are represented by $Z_i \sim N(0, \sigma_Z^2)$ and $\varepsilon_{i,j} \sim N(0, \sigma_\varepsilon^2)$ are the individual-level residual errors.

7.2.1 Interpretation of the results of the primary analysis

The primary analysis aims to determine the futility, or otherwise, of Simvastatin with respect to the primary outcome. Recall the hypotheses in section 4.2:

$$H_0: \mu_s - \mu_p \leq -3 \text{ versus } H_1: \mu_s - \mu_p > -3.$$

Testing of this will be achieved by determining whether the adjusted treatment coefficient estimate, δ_s from Equation 2, is less than or equal to -3, based on the test statistic from chapter 8 in Ravina, et al. [33]:

$$\frac{\delta_s - (-3)}{\sigma_s} \sim t_\eta$$

Equation 3

where σ_s is the standard error for the estimated coefficient and t is student's t distribution with η degrees of freedom. The degrees of freedom (η) is calculated from the total sample size minus the number of treatment groups minus number of adjustment covariates. The p-value for this test is determined by examining the upper tail of student's t-distribution. Possible scenarios for the futility analysis of Simvastatin are presented in Figure 2.

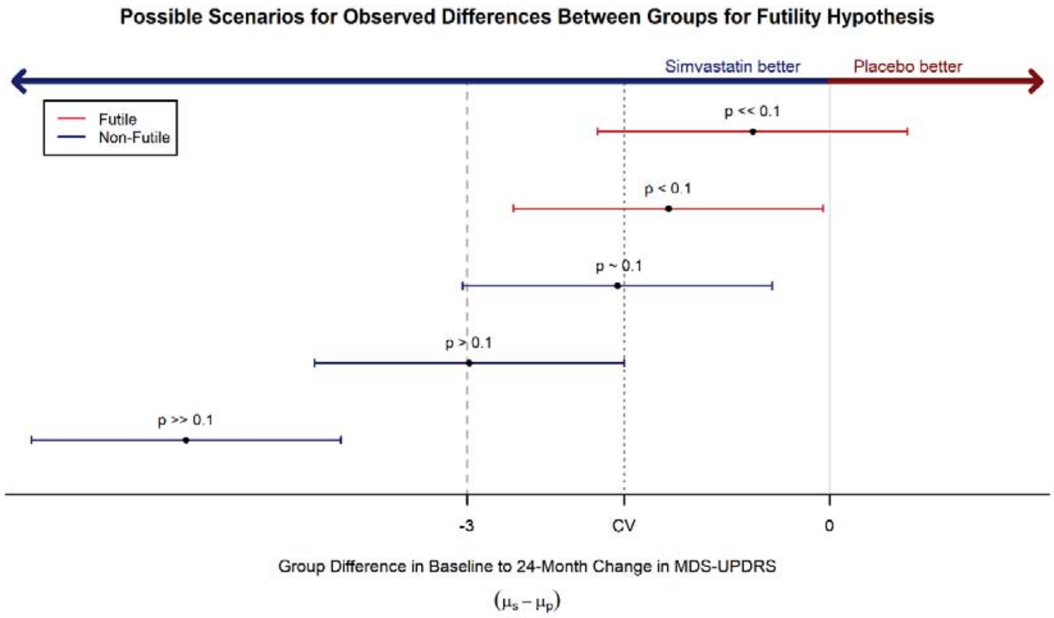

Figure 2: Possible scenarios for observed group differences in the change of MDS-UPDRS between baseline and 24 months under the assumptions of a futility analysis. The black circles are the between group differences in change in UPDRS at 24 months (point estimate). The red lines are two-sided 80% confidence intervals if the study is futile (i.e. p < 0.1) and blue lines are two-sided 80% confidence interval if the study is non-futile (i.e. p ≥ 0.1). The dashed grey line at -3 is the pre-specified minimal clinically important difference. The dotted grey line at CV is the critical value from student's t-distribution; the point estimate should be above this value (to the right of this line) to demonstrate futility.

If the p-value is less than 0.1, then the null hypothesis (that Simvastatin is not futile) will be rejected and Simvastatin will not be recommended for a phase III study. If the p-value is greater than 0.1 then there is insufficient evidence to reject the null hypothesis that Simvastatin is not futile.

P-value	80% Confidence Interval	Recommendations
~ 0.1	Does not contain -3	Consider Simvastatin futile.
≥ 0.1	Contains -3	• $\delta_s > -3$: Consider Simvastatin not futile<br>• $\delta_s \leq -3$: Consider further analysis of collected data for superiority.
>> 0.1	Upper bound below -3	Consider further analysis of collected data for superiority.

Table 2: Suggested recommendations based on the results of the futility analysis of the primary outcome if the null hypothesis is not rejected.

In addition to considering the calculated p-value, it is important to consider the two-sided 80% confidence interval to aid in interpreting the results of the primary analysis. Recommendations based on the two-sided 80% confidence interval, if the p-value is 0.1 or greater, are outlined in Table 2. However, these recommendations may require additional consideration should the original assumptions made for the sample size calculation prove to be invalid. The final decision on the futility or otherwise of Simvastatin should also take into consideration the results from the secondary outcomes and safety analysis.

7.3 Sensitivity Analyses of the Primary Outcome

Inferential sensitivity analyses will only be performed if the pre-specified criteria outlined in each of the following sections are met. Otherwise, appropriate descriptive statistics of the primary outcome by compliance (according to the pre-specified definitions given in section 4.4.2) will be presented.

7.3.1 Non-Compliance Sensitivity Analysis

Frequency and percentages of non-compliers for each of the compliance definitions outlined in section 4.4.2 will be reported. If ≥ 20% of participants with 24-month outcome data are classed as non-compliers based on either compliance definition (i) or definition (ii) from section 4.4.2, a sensitivity analysis based on that definition will be triggered. Therefore, a maximum of two non-compliance sensitivity analyses will be performed.

One potential method for sensitivity analyses is a per protocol analysis, which would only analyse participants who completed the trial as indicated in the protocol. However, this approach could introduce bias by excluding participants after randomisation and thus jeopardising the group comparability achieved through randomisation.

As an alternative, a complier-average causal effect (CACE) analysis will be undertaken if triggered according to the criteria above, which will provide an unbiased estimate of the intervention effect, based on protocol compliance[34]. In a CACE analysis, participant's allocated group is included in the model as an instrumental variable and the participant's given treatment is an endogenous variable, which will consist of placebo or intervention. The definitions of compliance in section 4.4.12 will be used to determine the participants' given treatment.

Under compliance definition (i), intervention participants' given treatment (the endogenous variable) would remain as intervention only if they proceeded to and maintained the 80mg dose and attended their 24-month follow-up assessment within the 2-week window. If they did not proceed to the 80mg dose, or if they did not attend their 24-month follow-up within the 2-week window, they would be classed as non-compliers and their given treatment would be classified as placebo.

Compliance definition (ii) is less stringent than compliance definition (i); participants in the intervention group will be classed as compliers and their given treatment will remain as intervention even if they do not proceed to the 80mg dose, provided they attend their 24-month assessment within the 2-week window and at least remain on the 40mg dose. Otherwise, their given treatment will become placebo.

It is possible that participants may be prescribed a statin, if clinically indicated, external to the study, at which point their allocated trial treatment will be discontinued, but they will be eligible to continue with follow-up. For participants allocated to the intervention who are prescribed statins at least equivalent to the study treatment (80mg under compliance definition (i); 40mg under compliance definition (ii)), their given treatment will remain as intervention; otherwise, their given treatment will be specified as placebo. In circumstances where a placebo allocated participant is prescribed statins within three months of commencing the study (i.e. minimum of 21 months on clinically indicated statin) and provides primary outcome data at 24 months, a clinical decision will determine whether the prescribed statin is equivalent to either 80mg or 40mg of Simvastatin.

If the clinical decision indicates the prescribed statin is at least equivalent to either dose of study treatment, the participant's given treatment will change to intervention accordance to the compliance definition; otherwise, they will remain as placebo.

With the exception of clinically indicated statin prescription outlined previously, under both compliance definitions, the given treatment of non-compliant participants allocated to the placebo arm will remain as placebo.

As a consequence of the planned CACE analyses, the group allocations will have to be known in order to identify non-compliers in the intervention group. Therefore, this analysis will be conducted following completion of the primary analysis of the primary outcome, once the statistician is unblinded.

Interpretation of the results from the CACE analyses will be based on testing the one-sided futility hypothesis, as detailed in section 7.2.

7.3.2 Location of Face-to-Face Visit Sensitivity Analysis

Participants' face-to-face visits could be conducted in an outpatient clinic or at home. To determine if the location of the visit has an effect on the primary outcome, irrespective of the group allocation, a sensitivity analysis will be performed in which the location of visit is included as a fixed effect covariate, alongside the previously defined adjustments in section 6.2. This fixed effect covariate will have four levels: (i) baseline and 24 month follow-up visit in clinic; (ii) baseline visit in clinic, 24 month follow-up visit at home; (iii) baseline visit at home, 24 month follow-up visit in clinic and (iv) both visits at home. Visits completed not face-to-face (i.e. visits completed remotely due to COVID-19 outbreak) will be excluded from this sensitivity analysis.

7.3.3 COVID-19 Sensitivity Analysis

The EMA draft guidance[35] on the implications of COVID-19 on methodological aspects of ongoing trials recommends additional analyses are undertaken to investigate three key phases (pre, during and post COVID-19), in order to understand the impact of the outbreak on the estimated treatment effect. As PD-STAT was nearing completion at the start of the outbreak, only two of these phases (pre and during COVID-19) will be applicable.

The primary outcome, MDS-UPDRS part III scores in the OFF state at 24 months, will be imputed as described in section 4.10.1, despite the fact that there will be more than three items missing for participants assessed during the COVID-19 lock down, due to the limitations of remote data collection outlined in 4.10.3. In order to facilitate a fair comparison between the COVID-19 and non-COVID-19 populations (defined in section 3.3.1), two models will be fitted to all participants' imputed total scores for (i) the COVID-19 population and (ii) the non-COVID-19 population. Interpretation of the analysis will focus on informal comparison of the coefficient estimates of allocated group difference between each model.

7.4 Secondary Analyses of the Primary Outcome

7.4.1 Repeated Measure Model

To ensure maximal use of the available primary outcome data, a repeated measures, mixed effects model[36] will be fitted to the primary outcome measure at baseline, 12, and 24 months, where participants are included as a random effect.

The model will be adjusted for participant's age at baseline, gender and disease duration, as well as the stratification variables (Hoehn & Yahr score as a fixed effect and recruiting site as a random effect). The model will include visit (baseline, 12 months and 24 months) and the interaction between visit and allocated group, which will be the coefficient of interest.

The repeated measure model is defined as

$$y_{i,j,t} = \delta_1 t + \delta_2 x_{1,i,j} I_{t=12} + \delta_3 x_{1,i,j} I_{t=24} + \boldsymbol{\theta} \boldsymbol{x}_{i,j} + Z_i + U_{i,j} + \varepsilon_{i,j,t}.$$

Equation 4

where

- $I_{t=k} = \begin{cases} 0 \text{ if } t \neq k \\ 1 \text{ if } t = k \end{cases}$ for $k = 12, 24$ is the visit month
- $y_{i,j,t}$ is the MDS-UPDRS score at visit month t
- δ_1 is the slope coefficient for time t
- δ_2 is between group difference at 12 months
- δ_3 is the between group difference at 24 months
- $U_{i,j} \sim N(0, \sigma_u^2)$ is the random effect for participant j in cluster i, independent of Z_i
- $\varepsilon_{i,j,t} \sim N(0, \sigma_\varepsilon^2)$ is the individual residual error at month t.

The terms x_1, $\boldsymbol{x}_{i,j}$, $\boldsymbol{\theta}$, Z_i, i and j are the same as defined for Equation 2, except the baseline score which is now included as an outcome instead of a fixed effect covariate.

7.4.2 Disease-Modifying Analysis

If there is evidence from the primary analysis that Simvastatin is not futile, consideration will be given to performing additional analysis of the primary outcome to investigate a potential disease-modifying effect. Descriptive statistics (e.g. means and 95% CI) and plots of MDS-UPDRS part III by visit month and allocated group will be presented. Participants will be included in the disease modifying analysis if: (i) they have fully complied with the treatment protocol (i.e. definition (i) from section 4.4.2) and (ii) they have provided valid MDS-UPDRS III scores at least two time points according to the imputation requirements outlined in section 4.10.1.

A mixed effects, repeated measures model will be fitted to the primary outcome measured at baseline, 12, 24 and 26 months, with participants included as a random effect. This repeated measures model will allow for missing data, whilst also allowing for analysis of the two-period design of the washout phase[37].

Adjustments will also be made for the stratification variables, modified Hoehn & Yahr score as a fixed effect and recruiting site as a random effect, as well as patient-related factors, which may have a strong influence on disease progression, i.e. disease duration, gender and age at baseline, to account for potential influence on the primary outcome[38].

The repeated measure model to investigate a disease modifying effect is defined as

$$y_{i,j,t} = \delta^{12*} x_{1,i,j} I_{t=12} + \delta^{24*} x_{1,i,j} I_{t=24} + \delta^{26*} x_{1,i,j} I_{t=26} + \delta_1 \boldsymbol{t} + \boldsymbol{\theta} \boldsymbol{x}_{i,j} + Z_i + U_{i,j} + \varepsilon_{i,j,t}$$

Equation 5

where δ^{k*} is the allocated group difference at the k visit month. The terms $y_{i,j,t}$, x_1, δ_1, $\boldsymbol{x}_{i,j}$, $\boldsymbol{\theta}$, Z_j, $U_{i,j}$, $\varepsilon_{i,j,t}$, $I_{\mathrm{t}=k}$, i and j are the same as defined in Equation 4, but $k = 12, 24, 26$, which include the 26-month visit and excludes baseline.

The study is not powered to determine whether the treatment is disease modifying. Therefore, the results of this analysis should be treated with caution, and interpreted as exploratory only. However, interpretation will focus on δ^{26*}, where an estimate below zero may be indicative of a effect that persists after discontinuation of treatment, suggesting the possibility that the effect of simvastatin is disease modifying rather than merely symptomatic.

The duration of the washout period of two months was a pragmatic decision. Currently there is no literature defining an appropriate washout period that would ensure any potential symptomatic effect of Simvastatin on PD had completely disappeared.

7.4.2.1 Disease-Modifying COVID-19 Sensitivity Analysis

To ascertain the possible impact of COVID-19 on the results of the disease-modifying analysis, a sensitivity analysis will be performed. The repeated measure model defined in Equation 5 will be fitted to the MDS-UPDRS part III scores in the OFF state, with imputation of the total scores undertaken regardless of the number of missing items, and will include an indicator variable that identifies whether the visit was completed during COVID-19 lockdown. The estimated coefficient and associated 95% confidence interval of the COVID-19 indicator variable will be used to understand the effect of COVID-19 on the disease-modifying analysis.

7.5 Superiority Analysis

If there is clearly insufficient evidence to conclude futility, there is potential for analysis of the primary outcome to assess superiority (see Appendix C for correspondence).

Recall the null hypothesis for the futility design from section 4.2:

$$H_0: \mu_s - \mu_p \leq -3.$$

This is the *pre-specified superiority.* Now consider the null hypothesis of a superiority design (i.e. *zero treatment effect*),

$$H_0^{(s)}: \mu_s - \mu_p = 0.$$

These two parameter subspaces are mutually exclusive, such that if one null hypothesis is true the other must be false. Therefore, at most only one type I error can ever be committed, whatever the nature of the true state may be. We conclude there is no need to adjust for type I error rate inflation, and there are no multiple testing issues associated with a further superiority analysis. A visual example of potential confidence intervals, for both futility and superiority analyses, are presented in Figure 3.

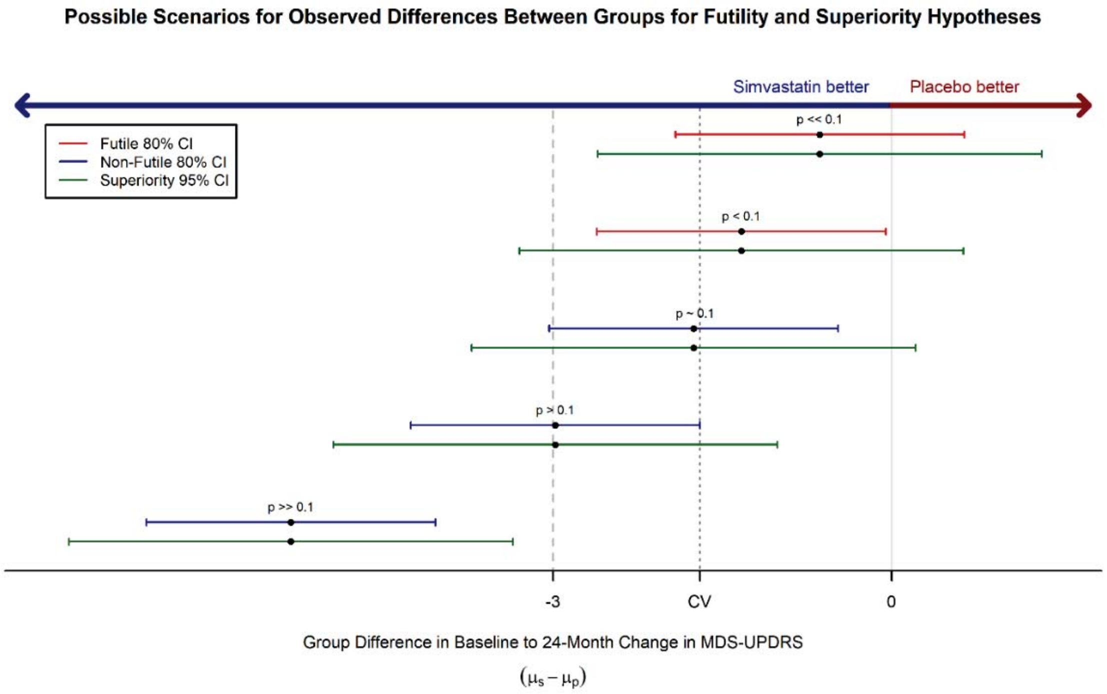

Figure 3: Possible scenarios for observed group differences in the change of MDS-UPDRS between baseline and 24 months under the assumptions of a futility and superiority analysis. The black circles are the difference between group means (point estimate). The red lines are two-sided 80% confidence intervals if the study is futile (i.e. $p < 0.1$) and blue lines are two-sided 80% confidence interval if the study is non-futile (i.e. $p \geq 0.1$). The green lines are the 95% confidence interval estimated with the same data under the assumptions for a superiority analysis. The dashed grey line at the MCID is the minimal clinically important difference. The dotted grey line at CV is the critical value from student's t-distribution; the point estimate should be above value (to the right of this line) to demonstrate futility.

8 Analysis of Secondary Outcomes

8.1 Secondary Outcome Variables

Where available, the secondary outcomes will be analysed:

(i) at 24 months;

(ii) using repeated measures modelling to include data at baseline, 12 and 24 months.

A list of the secondary outcomes, whether the patient was measured in the ON or OFF state and the objective of the measures are presented in Table 3.

Outcome	State	Objective
MDS-UPDRS part II	ON	Impact on motor experiences of daily living
MDS-UPDRS Total	ON	Impact on burden and extent of PD
Brain tap test *(number of key presses in 30 seconds)*	OFF	Impact on timed motor test
10MWT *(successfully completed and mean time to complete)*	OFF	Impact on timed motor test and distinguish symptomatic from disease modifying effects
MADRS Total score	ON	Impact on mood
ACE-III Total score	ON	Impact on cognitive abilities
NMSS Total score	ON	Impact on non-motor symptoms
KPPS Total score	ON	Impact on non-motor symptoms
PDQ-39 Total score	ON	Impact on activities of daily living
Total cholesterol level		Safety of Simvastatin
HDL cholesterol level		Safety of Simvastatin
Total/HDL cholesterol ratio		Safety of Simvastatin
Incidents of diabetes at 24 months		Safety of Simvastatin
LED		Distinguish symptomatic from disease modifying effects
Number of capsules every 6 months		Tolerability of simvastatin and trial protocol

Table 3: Secondary Outcomes and the objectives they will be used to assess.

8.2 Secondary Outcomes Analysis

Continuous secondary outcome measures will be analysed:

(i) at 24 months using mixed effects, linear regression models;

(ii) across baseline, 12 and 24 months using repeated measures linear models.

Since incidence of diabetes is a binary measure only observed at 24 months, this secondary outcome will be modelled using a mixed effects logistic regression model.

The models will be adjusted by the covariates described in section 6.2, with the exception of the repeated measures models, which will not include the baseline measure as a covariate (as baseline data are included in the outcome being modelled).

As not all participants will be able to complete the 10MWT, the ability to complete the test, as well as the time, are both valuable indicators of PD progression. Therefore, we will analyse time and successful completion of the 10MWT as two components of this outcome measure.

We will model completing the 10MWT as:

- binary data – whether participant successfully completed at least one 10MWT (yes or no);
- continuous data - mean time, of participants who are able to complete at least one 10MWT.

The number of capsules and percentage of expected capsules returned every month will be reported for both of the allocated groups.

The secondary outcomes will not be tested under a futility hypothesis framework: allocated group differences will be assessed at the two-sided 5% level of significance and reported with two-sided 95% confidence intervals. As the trial is not powered to detect clinically meaningful differences on the secondary outcome measures, and no adjustments for multiplicity will be made, the results of the analyses of secondary outcomes will be interpreted and reported as exploratory only.

8.2.1 COVID-19 Sensitivity Analyses of Secondary Outcomes

In line with the draft guidance on the implications of the COVID-19 outbreak on methodological aspects of ongoing clinical trials[35], additional sensitivity analyses of all secondary outcomes for which at least one participant provided valid 24-month data during COVID-19 lockdown will be undertaken. Specifically, the analyses of each of the secondary outcomes will be repeated excluding participants who provided 24-month outcome data during COVID-19 lockdown when social distancing measures were in place (i.e. the non-COVID-19 population). Comparison of the treatment effect estimates from these sensitivity analyses against the estimates obtained from the primary analyses of the secondary outcomes will facilitate informal evaluation of the effect of the pandemic on the secondary outcomes.

The number of capsules and percentage of capsules returned every month will also be presented according to whether 24-month outcome data was collected face-to-face or remotely.

9 SAFETY DATA

Adverse event (AE) data gathered from telephone contacts and visits of participants included in the safety population will be used to determine the safety and tolerability of Simvastatin.

The number and percentage of adverse events (AEs) will be reported by participant's completion status (i.e. completed IMP, discontinued IMP and withdrew) and by treatment taken (i.e. placebo, 40mg only or 80mg). We will also report information on:

- Timing of withdrawal/discontinuation
- Severity of AEs prior to withdrawal/discontinuation
- Classification of AEs prior to withdrawal/discontinuation
- Whether the decision to withdraw/discontinue was blinded.

We will report summary statistics of AEs by treatment taken, and if the number of participants reporting at least one AE within an organ system classification exceeds 5% of the safety population, we will report the AE summary statistics by treatment taken for that particular organ system. The summary statistics of serious adverse events (SAEs) will be reported by treatment taken and organ system classification.

Any AEs and/or SAEs which are related to common known effects side of statins use, as listed in the BNF [39], will be reported by treatment taken. These include:

- Asthenia
- Constipation
- Diarrhoea
- Dizziness
- Flatulence
- Gastrointestinal discomfort
- Hepatitis
- Headache
- Myalgia
- Myopathy (including myositis)
- Nausea
- Sleep disorder
- Thrombocytopenia

The summary statistics of AEs and SAEs will include:

- Numbers and percentages of participants reporting at least one AE
- Numbers and percentages of participants reporting AEs by the total number of AEs participants have reported
- Numbers and percentages of participants reporting at least one SAE
- Numbers and percentages of participants reporting SAEs by the total number of SAEs participants have reported

In addition, we shall produce Kaplan-Meier curves of cumulative incidences of AEs and SAEs by treatment taken.

Although no formal hypothesis testing of the safety data will be conducted, 95% confidence intervals of proportions by treatment taken will be presented. Any notable increase in safety related events within the Simvastatin groups (40mg only or 80mg) compared to the control group will be investigated further.

10 REFERENCES

1. Carroll CB, Webb D, Stevens KN, et al. Simvastatin as a neuroprotective treatment for Parkinson's disease (PD STAT): protocol for a double-blind, randomised, placebo-controlled futility study. *BMJ Open* 2019;9(10):e029740. doi: 10.1136/bmjopen-2019-029740 [published Online First: 2019/10/09]

2. Friedman B, Lahad A, Dresner Y, et al. Long-Term Statin Use and the Risk of Parkinson's Disease. *Am J Manag Care* 2013;19(8):626-32.

3. Undela K, Gudala K, Malla S, et al. Statin use and risk of Parkinson's disease: a meta-analysis of observational studies. *J Neurol* 2013;260(1):158-65. doi: 10.1007/s00415-012-6606-3

4. Chataway J, Schuerer N, Alsanousi A, et al. Effect of high-dose simvastatin on brain atrophy and disability in secondary progressive multiple sclerosis (MS-STAT): a randomised, placebo-controlled, phase 2 trial. *Lancet* 2014;383(9936):2213-21. doi: 10.1016/S0140-6736(13)62242-4

5. Brundin P, Barker RA, Conn PJ, et al. Linked Clinical Trials - The Development of New Clinical Learning Studies in Parkinson's Disease Using Screening of Multiple Prospective New Treatments. *J Parkinson Dis* 2013;3(3):231-39. doi: 10.3233/Jpd-139000

6. Gamble C, Krishan A, Stocken D, et al. Guidelines for the Content of Statistical Analysis Plans in Clinical Trials. *Jama-J Am Med Assoc* 2017;318(23):2337-43. doi: 10.1001/jama.2017.18556

7. Schulz KF, Altman DG, Moher D, et al. CONSORT 2010 Statement: updated guidelines for reporting parallel group randomised trials. *Bmj-Brit Med J* 2010;340 doi: 10.1136/bmj.c332

8. Calvert M, Blazeby J, Altman DG, et al. Reporting of Patient-Reported Outcomes in Randomized Trials The CONSORT PRO Extension. *Jama-J Am Med Assoc* 2013;309(8):814-22. doi: 10.1001/jama.2013.879

9. Ioannidis JP, Evans SJ, Gotzsche PC, et al. Better reporting of harms in randomized trials: an extension of the CONSORT statement. *Ann Intern Med* 2004;141(10):781-8. doi: 10.7326/0003-4819-141-10-200411160-00009 [published Online First: 2004/11/17]

10. Goetz CG, Tilley BC, Shaftman SR, et al. Movement Disorder Society-sponsored revision of the Unified Parkinson's Disease Rating Scale (MDS-UPDRS): Scale presentation and clinimetric testing results. *Movement Disord* 2008;23(15):2129-70. doi: doi:10.1002/mds.22340

11. Noyce AJ, Nagy A, Acharya S, et al. Bradykinesia-Akinesia Incoordination Test: Validating an Online Keyboard Test of Upper Limb Function. *PLOS ONE* 2014;9(4):e96260. doi: 10.1371/journal.pone.0096260

12. Steffen T, Seney M. Test-Retest Reliability and Minimal Detectable Change on Balance and Ambulation Tests, the 36-Item Short-Form Health Survey, and the Unified Parkinson Disease Rating Scale in People With Parkinsonism. *Physical Therapy* 2008;88(6):733-46. doi: 10.2522/ptj.20070214

13. Anette S, Paolo B, G. BR, et al. Depression rating scales in Parkinson's disease: Critique and recommendations. *Movement Disord* 2007;22(8):1077-92. doi: doi:10.1002/mds.21333

14. Leentjens AFG, Verhey FRJ, Lousberg R, et al. The validity of the Hamilton and Montgomery-Åsberg depression rating scales as screening and diagnostic tools for depression in Parkinson's disease. *International Journal of Geriatric Psychiatry* 2000;15(7):644-49. doi: doi:10.1002/1099-1166(200007)15:7<644::AID-GPS167>3.0.CO;2-L

15. Reyes MA, Lloret SP, Gerscovich ER, et al. Addenbrooke's Cognitive Examination validation in Parkinson's disease. *European Journal of Neurology* 2009;16(1):142-47. doi: doi:10.1111/j.1468-1331.2008.02384.x

16. Martinez-Martin P, Rodriguez-Blazquez C, Abe K, et al. International study on the psychometric attributes of the Non-Motor Symptoms Scale in Parkinson disease. *Neurology* 2009;73(19):1584-91. doi: 10.1212/WNL.0b013e3181c0d416

17. Jenkinson C, Fitzpatrick R, Peto V, et al. The Parkinson's Disease Questionnaire (PDQ-39): development and validation of a Parkinson's disease summary index score. *Age Ageing* 1997;26(5):353-7.

18. Ray CK, A. R, C. T, et al. King's Parkinson's disease pain scale, the first scale for pain in PD: An international validation. *Movement Disord* 2015;30(12):1623-31. doi: doi:10.1002/mds.26270

19. Herdman M, Gudex C, Lloyd A, et al. Development and preliminary testing of the new five-level version of EQ-5D (EQ-5D-5L). *Quality of Life Research* 2011;20(10):1727-36. doi: 10.1007/s11136-011-9903-x

20. Shulman LM, Gruber-Baldini AL, Anderson KE, et al. The Clinically Important Difference on the Unified Parkinson's Disease Rating Scale. *Arch Neurol-Chicago* 2010;67(1):64-70.

21. Tilley BC, Palesch YY, Kieburtz K, et al. Optimizing the ongoing search for new treatments for Parkinson disease - Using futility designs. *Neurology* 2006;66(5):628-33. doi: DOI 10.1212/01.wnl.0000201251.33253.fb

22. Aviles-Olmos I, Dickson J, Kefalopoulou Z, et al. Exenatide and the treatment of patients with Parkinson's disease. *J Clin Invest* 2013;123(6):2730-36. doi: 10.1172/Jci68295

23. Redmond CK, Colton T. Biostatistics in clinical trials: John Wiley & Sons 2001.

24. van Hout B, Janssen MF, Feng Y-S, et al. Interim Scoring for the EQ-5D-5L: Mapping the EQ-5D-5L to EQ-5D-3L Value Sets. *Value in Health* 2012;15(5):708-15. doi: https://doi.org/10.1016/j.jval.2012.02.008

25. Tomlinson CL, Stowe R, Patel S, et al. Systematic review of levodopa dose equivalency reporting in Parkinson's disease. *Movement Disord* 2010;25(15):2649-53. doi: 10.1002/mds.23429

26. Cervantes-Arriaga A, Rodríguez-Violante M, Villar-Velarde A, et al. Cálculo de unidades de equivalencia de levodopa en enfermedad de Parkinson. *Arch Neurocien* 2009;14(2):116-19.

27. Goetz CG, Luo S, Wang L, et al. Handling missing values in the MDS-UPDRS. *Mov Disord* 2015;30(12):1632-8. doi: 10.1002/mds.26153 [published Online First: 2015/02/05]

28. Keezer MR, Wolfson C, Postuma RB. Age, Gender, Comorbidity, and the MDS-UPDRS: Results from a Population-Based Study. *Neuroepidemiology* 2016;46(3):222-7. doi: 10.1159/000444021 [published Online First: 2016/03/12]

29. Skorvanek M, Martinez-Martin P, Kovacs N, et al. Differences in MDS-UPDRS Scores Based on Hoehn and Yahr Stage and Disease Duration. *Mov Disord Clin Prac* 2017;4(4):536-44. doi: 10.1002/mdc3.12476

30. Committee for Human Medicinal Products. Guideline on adjustment for baseline covariates in clinical trials (EMA/CHMP/295050/2013). Eurpean Medicines Agency, 2015.

31. Stata Statistical Software: Release 14 [program]. College Station, TX: StataCorp LP., 2015.

32. R: A language and environment for statistical computing [program]. Vienna, Austria, 2017.

33. Ravina B, Cummings J, McDermott M, et al., editors. *Clinical Trials in Neurology: Design, Conduct, Analysis*. Cambridge: Cambridge University Press, 2012.

34. Dunn G, Maracy M, Tomenson B. Estimating treatment effects from randomized clinical trials with noncompliance and loss to follow-up: the role of instrumental variable methods. *Stat Methods Med Res* 2005;14(4):369-95. doi: 10.1191/0962280205sm403oa

35. Committee for Human Medicinal Products. Points to consider on implications of Coronavirus disease (COVID-19) on methodological aspects of ongoing clinical trials – Draft (EMA/158330/2020). European Medinces Agency, 2020.

36. Albert PS. Longitudinal data analysis (repeated measures) in clinical trials. *Stat Med* 1999;18(13):1707-32. doi: 10.1002/(sici)1097-0258(19990715)18:13<1707::aid-sim138>3.0.co;2-h [published Online First: 1999/07/17]

37. Molenberghs G, Thijs H, Jansen I, et al. Analyzing incomplete longitudinal clinical trial data. *Biostatistics* 2004;5(3):445-64. doi: 10.1093/biostatistics/kxh001

38. Lang AE, Melamed E, Poewe W, et al. Trial designs used to study neuroprotective therapy in Parkinson's disease. *Mov Disord* 2013;28(1):86-95. doi: 10.1002/mds.24997 [published Online First: 2012/08/29]

39. British Medical Association, Royal Pharmaceutical Society. British National Formulary (BNF) BMJ Group and Pharmaceutical Press; 2020 [updated 11/03/2020]. Available from: https://bnf.nice.org.uk/drug/simvastatin.html#sideEffects accessed 02/04/2020 2020.

40. Gale T, Hawley C. A model for handling missing items on two depression rating scales. *Int Clin Psychopharmacol* 2001;16(4):205-14. doi: 10.1097/00004850-200107000-00004 [published Online First: 2001/07/19]

41. Lees RA, Hendry Ba K, Broomfield N, et al. Cognitive assessment in stroke: feasibility and test properties using differing approaches to scoring of incomplete items. *Int J Geriatr Psychiatry* 2017;32(10):1072-78. doi: 10.1002/gps.4568 [published Online First: 2016/08/17]

42. Jenkinson C, Heffernan C, Doll H, et al. The Parkinson's Disease Questionnaire (PDQ-39): evidence for a method of imputing missing data. *Age Ageing* 2006;35(5):497-502. doi: 10.1093/ageing/afl055 [published Online First: 2006/06/15]

APPENDIX

A. ADDITIONAL INFORMATION FOR OUTCOME MEASURES

PD Medication	Conversion Factor
Amantadine	1
Apomorphine	10
Azilect	10
Bromocriptine	10
Cabergoline	80
Duodopa	1.11
Entacapone	0.33*
Levodopa dispersible	1
Levodopa immediate release	1
Levodopa controlled release	0.75
Lisuride	100
Madopar	1
Mirapex	100
Opicapone	0.5*
Pergolide	100
Pramipexole	100
Rasagiline	100
Requip	20
Ropinirole	20
Rotigotine	30
Rytary	0.6
Safinamide	100**
Selegiline Oral	10
Selegiline Sublingual	80
Sinemet	1
Sinemet controlled release	0.75
Stalevo	0.33*
Tolcapone	0.5*

* Multiply immediate release levodopa dose by conversion factor and add to immediate release levodopa dose.
** Add this value to the LED, regardless of dose.

Table 4: Conversion factors for levodopa-equivalent dose

Example: Immediate release Levodopa dose = 400 and Entacapone = 800, implies:

LED = (1 × 400) + (0.33 × 400) = 400 + 132 = 532

<table>
<tr><th>Secondary outcome</th><th>Missing Items</th></tr>
<tr><td>MDS-UPDRS part II</td><td rowspan="2">Goetz, et al. [27]

Imputation formula

$D = \frac{A \times B}{C}$

▪ A - sum of the items scored,
▪ B - total number of items which should have been scored
▪ C - number of items with actual score
▪ D - imputed score.

Part I missing a maximum of 1 item, part II missing maximum of 2 items, part III missing maximum of 3 items, part IV cannot have any missing items.</td></tr>
<tr><td>MDS-UPDRS Total</td></tr>
<tr><td>MDS-UPDRS part IV</td><td>For questions 1, 3 and 6, the score is based on the percentage reported in part C of each question, which is calculated by dividing the number of hours stated in part B by the number of hours stated in part A and multiplying by 100. For each of questions 1, 3 and 6, the question score is then:
• 0 if part B = 0
• 1 if part C ≤ 25%
• 2 if part C is 26-50%
• 3 if part C is 51-75%
• 4 if part C > 75%.

In instances where the ordinal score is missing but part C (the percentage) is available, the score will be determined based on the above thresholds of the reported part C. If the ordinal score and part C are missing but part A and B are available, part C (the percentage) will be calculated and the ordinal score determined by the above defined thresholds.</td></tr>
<tr><td>MADRS</td><td>For a maximum of 1 missing item

Use weighted means from Gale and Hawley [40] to calculate total MADRS score, see Table 6 for weights.</td></tr>
<tr><td>ACE-III</td><td>Participants with any missing items set total score to missing (approach 3, Lees, et al. [41]).</td></tr>
<tr><td>PDQ-39</td><td>Use EM algorithm but requires more than 200 participants[42].</td></tr>
</table>

Table 5: Methods for handling missing items for questionnaire data, where available

Item	Weight
1	1.03
2	1.14
3	1.13
4	1.20
5	0.69
6	1.18
7	1.15
8	1.11
9	0.89
10	0.47

Table 6: Item weights for the calculation of MADRS given one item is missing

B. Examples of Figures and Tables for Reporting Study Results

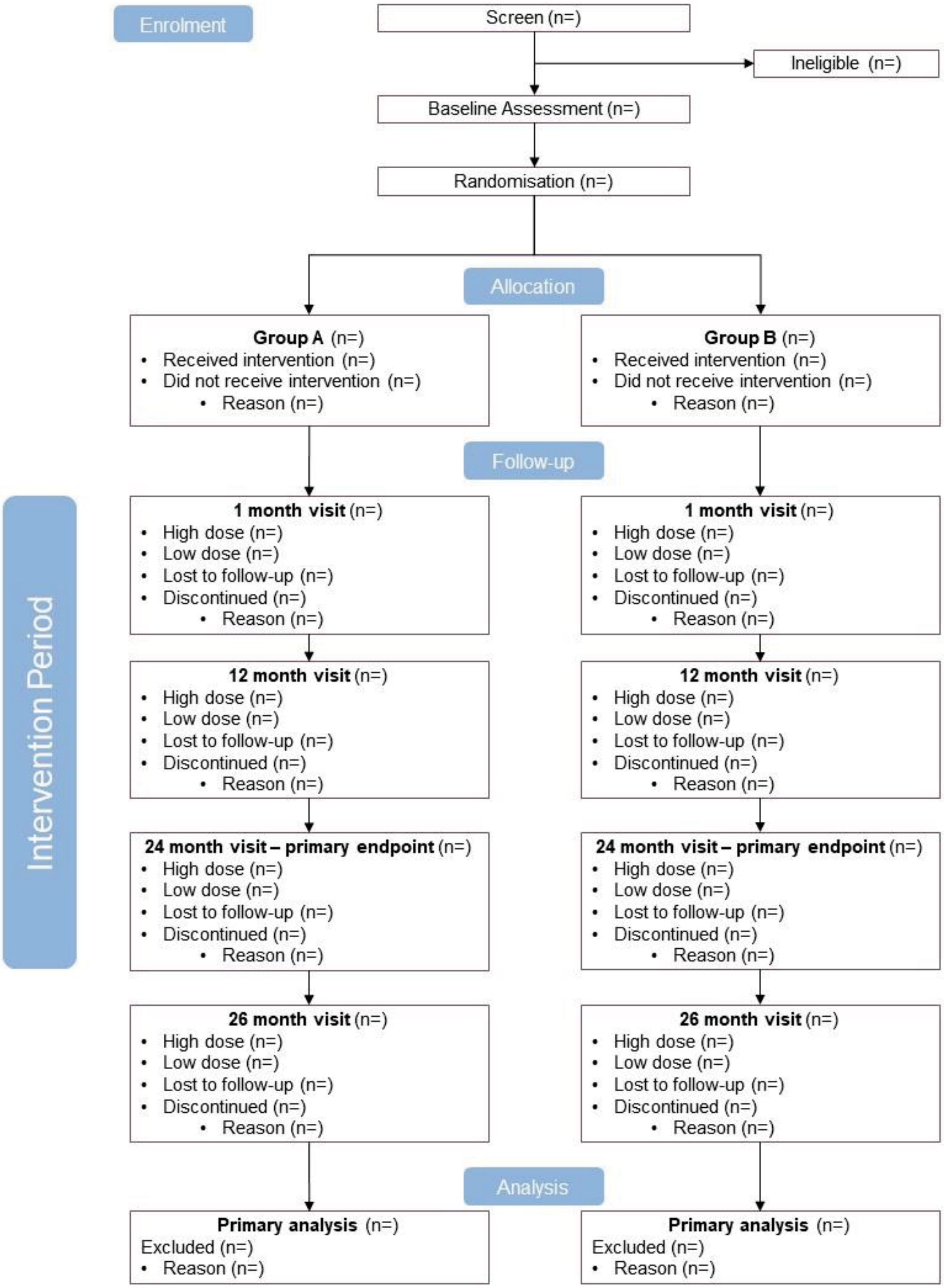

Figure 4: CONSORT Flow Diagram of participants through PD STAT.

	Simvastatin N (%)	Placebo N (%)	All N (%)
**Age in years**			
Mean (SD) [range]			
Median (IQR)			
**Female**			
**Ethnic Origin**			
White			
Black Caribbean			
Black African			
Chinese			
Indian			
Pakistani			
Bangladeshi			
Other			
**Smoking Status**			
Non smoker			
Ex-smoker			
Light Smoker			
Moderate smoker			
Heavy Smoker			
**Hoehn & Yahr**			
1.0			
1.5			
2.0			
2.5			
3.0			
**Age in years at onset PD**			
Mean (SD) [range]			
Median (IQR)			
< 30			
30 ≤ PD age < 40			
40 ≤ PD age < 50			
50 ≤ PD age < 60			
60 ≤ PD age			
**Disease duration**			
Mean (SD) [range]			
Median (IQR) [range]			
**Diabetes Status at screening**			
None			
Type 1			
Type 2			

Type 2[a]			
**Total Cholesterol (mmol/L)**			
Mean (SD) [range]			
Median (IQR)			
≤ 5 mmol/L			
> 5 mmol/L			
**QRISK® 2**<br>**Mean (SD)**			
< 10%			
≥ 10%			
**QRISK® 2**			
Median (IQR) [range]			
< 10%			
≥ 10%			
**MoCA**			
Mean (SD) [range]			
Median (IQR)			
**MADRS**			
Mean (SD) [range]			
Median (IQR)			
**AST**			
Mean (SD) [range]			
Median (IQR)			
**ALT**			
Mean (SD) [range]			
Median (IQR)			
**CK**			
Mean (SD) [range]			
Median (IQR)			

Table 7: Baseline and Demographic Data

	Mean (SD)	Fully Adjusted*		Adjusted Only for Baseline MDS-UPDRS Part III Score	
		Coeff. Est. (80% CI)	p-value	Coeff. Est. (80% CI)	p-value
**Primary analysis**					
Placebo					
Simvastatin					
**Sensitivity Analysis**					
Placebo					
Simvastatin					

* The analysis is adjusted for baseline MDS-UPDRS part III, recruiting site, modified Hoehn & Yahr score, disease duration, gender and age at baseline.

Table 8: Primary outcome analysis: linear regression of change in MDS-UPDRS score part III at 24 months in the OFF state.

Secondary Outcomes	State	Fully Adjusted* (95% CI)		Adjusted Only for Baseline Score (95% CI)	
		24 months	Repeated	24 months	Repeated
**MDS-UPDRS**					
Part II	ON				
Total	ON				
**Timed Motor Tests**					
Brain tap test	OFF				
Walk test	OFF				
**Questionnaire**					
MADRS	ON				
ACE-III	ON				
NMSS	ON				
PDQ-39	ON				
KPPS	ON				
**Cholesterol**					
Total	NA				
HDL	NA				
Total/HDL ratio	NA				
**Diabetes**	NA				
**LED**	NA				

EMS - electromagnetic sensor; MADRS - Montgomery and Asberg Depression Rating Scale; ACE-III -The Addenbrooke's Cognitive Assessment-III (ACE-III); NMSS - Non-Motor Symptom assessment scale (NMSS); PDQ-39 - Parkinson's disease Questionnaire (PDQ-39); LED - levodopa-equivalent dose; KPPS - King's PD pain scale; EQ-5D-5L - EuroQoL 5D-5L health status questionnaire.

* Differences are adjusted for outcome measure at baseline (where available), recruiting site and modified Hoehn & Yahr score, disease duration, gender and age at baseline.

Table 9: Differences between placebo and intervention group (i.e. placebo – intervention) with corresponding 95% confidence intervals for all secondary outcomes.

C. Superiority Analysis Correspondence

From: Siobhan Creanor <siobhan.creanor@plymouth.ac.uk>
Sent: Wednesday, September 18, 2019 1:41 PM
To: bruce.levin@columbia.edu
Subject: Futility studies

Dear Professor Levin

I'm emailing for some thoughts/advice regarding a current parallel group "futility/non-superiority" RCT that we are running in the UK. The trial, PD STAT (http://www.isrctn.com/ISRCTN16108482) is managed by the Peninsula Clinical Trials Unit, and is one of the early Linked Clinical Trials in The Cure Parkinson's Trust.

The trial is still in follow-up and we are currently finalising the statistical analysis plan for independent review by the trial oversight committees (in the UK, things are run a little differently to the US). I should add at this point, the trial team, including the statisticians, has not seen any data.

As a futility/non-superiority study, we are very clear on the null hypotheses, etc. However, throughout this trial I've been reflecting on some of the similarities/challenges with non-inferiority trials. One point that we are particularly thinking about is the following.

PD STAT was powered on a one-sided hypothesis, with one-sided alpha of 10% and 80% power (beta=20%). We have a pre-specified margin on non-superiority of 3 points.

Take a non-inferiority trial, with a one-sided primary hypothesis and say one-sided alpha of 5% and a pre-specified margin of non-inferiority, say again of 3 points. However, despite the one-sided hypothesis, the recommended approach (by the European Medicines Agency, EMA) for analysis of non-inferiority trials is to calculate the TWO-SIDED confidence interval – so for this example would be the two-sided 90% confidence interval. To 'test' the non-inferiority hypothesis, we look at whether this confidence interval lies completely on one side of the non-inferiority margin. And importantly, if non-inferiority can be declared, it is then possible to assess for superiority using the same two-sided confidence interval. From EMA (https://www.ema.europa.eu/en/documents/scientific-guideline/points-consider-switching-between-superiority-non-inferiority_en.pdf) "*If the 95% confidence interval for the treatment effect not only lies entirely above -Δ but also above zero then there is evidence of superiority in terms of statistical significance at the 5% level (p<0.05). See Figure 4. In this case it is acceptable to calculate the p-value associated with a test of superiority and to evaluate whether this is sufficiently small to reject convincingly the hypothesis of no difference. There is no multiplicity argument that affects this interpretation because, in statistical terms, it corresponds to a simple closed test procedure. Usually this demonstration of a benefit is sufficient on its own, provided the safety profiles of the new agent and the comparator are similar.*"

This makes me wonder if it would be appropriate/sensible to think of allowing for a similar pre-specified approach in PD STAT – i.e. firstly assess for futility and then, if the results indicate promise of efficacy, assess for "superiority".

I appreciate this is not how the trial was designed as such – and I suspect it is a "long shot" that PD STAT would show superiority. However, we currently have higher participant retention than we might have anticipated and given the primary endpoint is at 24 months (this has been an expensive

futility trial!), I think it's important to consider in advance that we 'may' have evidence to support superiority, in addition to non-futility.

Have you ever considered such an approach?

Kind regards

Siobhan

Professor of Medical Statistics & Clinical Trials
Director of PenCTU
Faculty of Medicine & Dentistry
University of Plymouth
Plymouth Science Park
Davy Road
Plymouth
PL6 8BX

From: Bruce Levin <bl6@columbia.edu>
Sent: 19 September 2019 00:35
To: Siobhan Creanor <siobhan.creanor@plymouth.ac.uk>
Subject: RE: Futility studies

Dear Professor Creanor,

Yes, I have considered such an approach and I'm happy to say it is valid, although for slightly simpler reasons than the one you quote from the EMEA. Let me give you that simpler reason first, in the context of futility testing, and then explain why it's a little different from the non-inferiority scenario.

Your futility (non-superiority) design tests one null hypothesis, let's call it $H_0^{(1)}$, namely, that the true treatment effect is greater than or equal to the pre-specified margin of superiority, call it □>0. You wish to test a second null hypothesis, call it $H_0^{(2)}$, which states that the true treatment effect is *less than or equal* to 0 (assuming one-sided testing) or that the true treatment effect is exactly equal to 0 (assuming two-sided testing), such that if you reject $H_0^{(2)}$ you can declare "superiority". Thinking about the parameter space of a possible true treatment effects, the two null hypotheses comprise *disjoint* subspaces. In plain English, if $H_0^{(1)}$ is true then $H_0^{(2)}$ cannot be true and *vice versa*. Therefore, one cannot possibly make more than one type I error under any true state of nature, because there can only ever be (at most) one true null hypothesis. Therefore, the family-wise type I error rate is controlled at level alpha and there is no inflation of type I error. Voilà!

In the case of a non-inferiority trial, the EMEA cites the closed testing principle because it is implicitly contemplating two ***overlapping*** null hypotheses, namely, $H_0^{(1)}$: the true treatment effect is *less than or equal* to –□, and $H_0^{(2)}$: the true treatment effect is *less than or equal* to 0. The closed test principle allows us to test both hypotheses with no inflation of type I error because in order to reject $H_0^{(2)}$, by the closed test principle one must reject *both* $H_0^{(1)}$ *and* $H_0^{(2)}$, because the intersection of $H_0^{(1)}$ and $H_0^{(2)}$ in this case is just $H_0^{(1)}$. However, because of the structure of the hypothesis space, if one rejects $H_0^{(2)}$ one *automatically* rejects $H_0^{(1)}$ by an *a fortiori* argument (picture the critical

regions). [This argument is for one-sided testing of $H_0^{(2)}$; if one wanted to conduct a two-sided test, the above argument by disjointedness would suffice.]

At this point, please note the structure of what is being considered in non-inferiority testing: *if* one already rejects $H_0^{(1)}$ and declares non-inferiority, one is then naturally interested in testing the "home-run" hypothesis $H_0^{(2)}$, hoping actually to declare superiority.

Now let's look in the mirror to see what this says about non-superiority testing. The analog would be $H_0^{(1)}$ as we originally defined it, that the true treatment effect is *greater than or equal* to □ and if one already rejects that, one might be interested in testing the "anti-home-run" hypothesis, namely, $H_0^{(2)}$: the true treatment effect is *actually worse than* 0. The closed test principle again allows you to do this without inflation of type I error, again, because in order to reject $H_0^{(2)}$ you have to reject *both* $H_0^{(1)}$ and $H_0^{(2)}$, but if you reject $H_0^{(2)}$ you must already reject $H_0^{(1)}$.

But this was not the pair of hypotheses you were inquiring about! So, yes, go ahead and pre-specify the one-sided $H_0^{(2)}$: true treatment effect is less than or equal to 0 and reject it if and only if the lower endpoint of your 100%×(1–2□) confidence interval lies above 0. If so, even if you reject the original null hypothesis $H_0^{(1)}$ of superiority and declare "futility" (more precisely, non-superiority), you can still declare the treatment effect significantly better than 0. Of course, by the logic of futility testing, that may be small consolation, because presumably the margin of superiority corresponds to the *minimal worthwhile treatment effect*, such that there would be a clinical lack of enthusiasm for proceeding with smaller effects, even if significantly different from 0.

Bruce

Bruce Levin, Ph.D.,
Professor Emeritus of Biostatistics

Past Chair, Department of Biostatistics
Columbia University
Mailman School of Public Health
722 West 168th Street
MSPH Box 12, 6th Floor
New York, NY 10032

(917) 734-0227 voice

Bruce.Levin@Columbia.edu
http://www.columbia.edu/~bl6
