# Supplementary material for: Evaluation of Simvastatin as a Disease-Modifying Treatment for Patients With Parkinson Disease: A Randomized Clinical Trial
Source: JAMA Neurol. 2022 Oct 31;79(12):1232–41. doi: 10.1001/jamaneurol.2022.3718 (PMC9623477; doi:10.1001/jamaneurol.2022.3718)
Supplement: Supplement 3. — eTable 1. Summary Statistics of the Baseline Characteristics by Allocated Group of All Recruited Participants eTable 2. Results of the COVID-19 Sensitivity Analyses of the Secondary Outcomes Mixed-Effects Linear Regression Models at 24 Months eTable 3. Frequency and Percentage of Serious Adverse Events (SAEs) by Reported Dose of Simvastatin at the Time of the SAE eTable 4. Frequency and Percentage of Related Adverse Events (AEs) by Reported Dose of Simvastatin at the Time of the Related AE eTable 5. Frequency and Percentage of Related Adverse Events (AEs) Identified as Common Adverse Effects of Simvastatin eAppendix. Acknowledgement of Site Contributions [file jamaneurol-e223718-s003.pdf]

# Supplemental Online Content

Stevens KN, Creanor S, Jeffery A, et al; the PD STAT Study Group. Evaluation of simvastatin as a disease-modifying treatment for patients with Parkinson disease: a randomized clinical trial. *JAMA Neurol*. Published online October 31, 2022.
doi:10.1001/jamaneurol.2022.3718

**eTable 1.** Summary Statistics of the Baseline Characteristics by Allocated Group of All Recruited Participants

**eTable 2.** Results of the COVID-19 Sensitivity Analyses of the Secondary Outcomes Mixed-Effects Linear Regression Models at 24 Months

**eTable 3.** Frequency and Percentage of Serious Adverse Events (SAEs) by Reported Dose of Simvastatin at the Time of the SAE

**eTable 4.** Frequency and Percentage of Related Adverse Events (AEs) by Reported Dose of Simvastatin at the Time of the Related AE

**eTable 5.** Frequency and Percentage of Related Adverse Events (AEs) Identified as Common Adverse Effects of Simvastatin

**eAppendix.** Acknowledgement of Site Contributions

This supplementary material has been provided by the authors to give readers additional information about their work.

© 2022 Stevens KN et al. *JAMA Neurology*.

**eTable 1.** Summary Statistics of the Baseline Characteristics by Allocated Group of All Recruited Participants

**Continuous covariates are reported by mean and standard deviation (SD), unless indicated and categorical covariates are reported by frequency and percentage.**

| | **Placebo N = 118** | **Simvastatin N = 117** | **All N = 235** |
|---|---|---|---|
| **Age in years** | 65·0 (9·7) | 65·9 (8·7) | 65·4 (9·2) |
| **Female** | 52 (44%) | 45 (38%) | 97 (41%) |
| **Disease duration in years** | 10·0 (4·8) | 9·6 (3·8) | 9·8 (4·3) |
| **Systolic Blood Pressure (mmHg)** | 129·7 (18·3) | 126·7 (16·5) | 128·2 (17·5) |
| **BMI (kg/m$^2$)** | 26·9 (4·7) | 26·2 (4·7) | 26·6 (4·7) |
| **MoCA** | 27·6 (2·5) | 27·7 (2·2) | 27·6 (2·4) |
| **Ethnic Origin** | | | |
| White | 117 (99%) | 116 (99%) | 233 (99%) |
| Other | 1 (1%) | 1 (1%) | 2 (1%) |
| **Smoking Status** | | | |
| Never-smoker | 81 (69%) | 77 (66%) | 158 (67%) |
| Ex-smoker | 33 (28%) | 35 (30%) | 68 (29%) |
| Light Smoker | 4 (3%) | 4 (3%) | 8 (3%) |
| Moderate smoker | 0 (0·0) | 1 (1%) | 1 (0·5%) |
| **Relationship Status** | | | |
| Single | 7 (6%) | 5 (4%) | 12 (5%) |
| Married/civil partnership | 97 (82%) | 98 (84%) | 195 (83) |
| Separated | 3 (3%) | 1 (1%) | 4 (2%) |
| Divorced/partnership dissolved | 6 (5%) | 8 (7%) | 14 (6%) |
| Widowed/surviving partner | 4 (3%) | 5 (4%) | 9 (4%) |
| **Living Status** | | | |
| Live alone | 10 (8%) | 13 (11%) | 23 (10%) |
| Spouse/partner | 104 (88%) | 101 (86%) | 205 (87%) |
| Parent(s) | 1 (1%) | ·· | 1 (0·5) |
| Children under 18 | 10 (8%) | 13 (11%) | 23 (10%) |
| Children over 18 | 19 (16%) | 15 (13%) | 34 (14%) |
| Non-family | 1 (1%) | 1 (1%) | 2 (1%) |
| **QRisk** | | | |
| N (%) ≥ 10% | 57 (48·3) | 53 (45·3) | 110 (46·8) |
| Median (IQR) | 9·6 (3·8 – 16·0) | 8·6 (6 - 15·9) | 9·2 (4·8 – 16·0) |
| **Hoehn & Yahr** | | | |
| 1·0 to 2·0 | 80 (68%) | 82 (70%) | 162 (69%) |
| 2·5 to 3·0 | 38 (32%) | 35 (30%) | 73 (31%) |

© 2022 Stevens KN et al. *JAMA Neurology*.

**eTable 2.** Results of the COVID-19 Sensitivity Analyses of the Secondary Outcomes Mixed-Effects Linear Regression Models at 24 Months

**The models were adjusted for sex, Hoehn and Yahr stratification variable and the participant's outcome measurement, age and PD duration at baseline as fixed effects and study centres as random effects.**

| Secondary Outcome | Fully Adjusted Mean Between-Group Difference (simvastatin minus placebo) (Two-sided 95% CI) |
|---|---|
| HDL (mmol/L) | 0·000148 (-0·147 to 0·150)* |
| Total cholesterol (mmol/L) | -1·15 (-1·41 to -0·881) |
| Total cholesterol/HDL ratio | -0·691 (-0·905 to -0·476) |
| BTT - key presses in 30 seconds (OFF) | -1·25 (-4·39 to 1·89) |
| Mean 10MWT time (seconds) [OFF] | -0·658 (-1·57 to 0·256)* |
| 10MWT completed at least one test [OFF]† | OR = 0·636 (0·192 to 2·10) |
| MDS-UPDRS part II [ON] | 1·06 (-0·450 to 2·57) |
| MDS-UPDRS Total Score [ON] | 0·413 (-4·18 to 5·00) |
| PDQ-39 Total Score | 0·571 (-2·30 to 3·44) |
| NMSS Total Score | 1·51 (-5·57 to 8·59)* |
| KPPS Total Score | 0·592 (-2·41 to 3·60)* |
| LED | -19·0 (-87·0 to 48·9) |
| HbA1c (mmol/mol) | 0·335 (-0·441 to 1·11) |
| Diabetes† | OR = 0·864 (0·0507 to 14·7) |
| ACE-III Total Score | 0·4 (-01·0 to 1·9)* |
| MADRS Total Score | -0·1 (-1·2 to 1·1) |

* Confidence interval calculated from bootstrapped standard error estimate

*† Summary statistics reported as frequency and percentage allocated group difference reported as odds ratio (OR)*

© 2022 Stevens KN et al. *JAMA Neurology*.

**eTable 3.** Frequency and Percentage of Serious Adverse Events (SAEs) by Reported Dose of Simvastatin at the Time of the SAE

| | Simvastatin Dose at time of SAE | |
|---|---|---|
| **N (%)** | **0mg*** | **80mg** |
| | **(N = 37)** | **(N = 37)** |
| **Reason for SAE**** | | |
| Prolonged/required hospitalisation | 34 | 33 |
| Important medical event | 7 | 10 |
| Persistent/significant disability/incapacity | 1 | 2 |
| Event fatal | 1 | .. |
| Life threatening | .. | 1 |
| **Outcome** | | |
| Recovered | 21 | 25 |
| Recovered with sequelae | 13 | 12 |
| Died | 3 | .. |
| **Related to trial treatment** | | |
| Possible | .. | 1 |
| Unlikely | 4 | 6 |
| Not related | 33 | 30 |
| **Organ Classification** | | |
| Surgical & medical procedures | 12 | 14 |
| Injury, poisoning & procedural complications | 4 | 4 |
| Gastrointestinal disorders | 3 | 5 |
| Infections & infestations | 5 | 1 |
| Nervous system disorders | 2 | 3 |
| Cardiac | 3 | 2 |
| Renal & urinary disorders | 1 | 2 |
| Neoplasms benign, malignant & unspecified | 2 | 1 |
| Respiratory, thoracic & mediastinal | 2 | .. |
| Investigations | .. | 2 |
| Hepatobiliary disorders | 2 | .. |
| General & administration site conditions | 1 | 1 |
| Eye disorders | .. | 1 |
| Endocrine | .. | 1 |

* Includes participants allocated to the placebo group and those who had discontinued simvastatin prior to the reported SAE
** SAE could be assigned more than one reason

© 2022 Stevens KN et al. *JAMA Neurology*.

**eTable 4.** Frequency and Percentage of Related Adverse Events (AEs) by Reported Dose of Simvastatin at the Time of the Related AE

| N | Simvastatin dose at time of related AE | | |
|---|---|---|---|
| | **0mg*** | **40 mg** | **80mg** |
| | **(N = 171)** | **(N = 66)** | **(N = 84)** |
| **Related to trial treatment** | | | |
| Definite | 3 | 2 | 2 |
| Probable | 12 | 6 | 14 |
| Possible | 156 | 58 | 68 |
| **Organ Classification** | | | |
| Musculoskeletal & connective tissue disorders | 65 | 30 | 62 |
| Gastrointestinal disorders | 16 | 20 | 16 |
| Nervous system disorders | 30 | 8 | .. |
| General & administration site conditions | 26 | 6 | 6 |
| Skin & subcutaneous tissue disorders | 10 | .. | .. |
| Respiratory, thoracic & mediastinal | 5 | 2 | .. |
| Investigations | 6 | .. | .. |
| Psychiatric disorders | 4 | .. | .. |
| Eye disorders | 4 | .. | .. |
| Cardiac | 3 | .. | .. |
| Vascular | 2 | .. | .. |
| **Common Known Side Effect** | **93** | **48** | **68** |

* Includes participants allocated to the placebo group and those who had discontinued simvastatin 24 hrs or more prior to the reported related AE

**eTable 5.** Frequency and Percentage of Related Adverse Events (AEs) Identified as Common Adverse Effects of Simvastatin

| N (%) | Simvastatin dose at time of related AEs identified as common side effect | | |
|---|---|---|---|
| | 0mg* | 40 mg | 80mg |
| | (N = 93) | (N = 48) | (N = 68) |
| Myalgia | 62 (63%) | 30 (63%) | 54 (79%) |
| Headache | 18 (18%) | ·· | ·· |
| Nausea | 6 (6%) | 8 (17%) | ·· |
| Gastrointestinal discomfort | 2 (2%) | 6 (13%) | 2 (3%) |
| Constipation | ·· | ·· | 8 (12%) |
| Diarrhoea | 2 (2%) | ·· | 4 (6%) |
| Sleep disorder | 2 (2%) | 2 (4%) | ·· |
| Flatulence | 2 (2%) | 2 (4%) | ·· |
| Asthenia | 4 (4%) | ·· | ·· |

© 2022 Stevens KN et al. *JAMA Neurology*.

**eAppendix.** Acknowledgement of Site Contributions

| | |
|---|---|
| **Derriford Hospital, Plymouth** | **Addenbrooke's Hospital, Cambridge** |
| Jemma Inches | Simone Hargreaves |
| Donna Underwood | Danielle Johnson |
| **Royal Cornwall Hospital, Truro** | Lucy Worboys |
| Julie Frost | Paul Worth |
| Ali James | **Salford Royal Hospital** |
| Christine Schofield | Judith Brooke |
| **Royal Devon and Exeter Hospital** | Christopher Kobylecki |
| Rob James | Victoria Parker |
| Clare O'Reilly | **Fairfield General Hospital, Bury** |
| Ray Sheridan | Judith Brooke |
| Sarah Statton | Linda Johnson |
| **Musgrove Park Hospital, Taunton** | Rosane Joseph |
| Tamlyn Russell | Julie Melville |
| Alison Whitcher | Jason Raw |
| **Yeovil District Hospital** | **Royal Preston Hospital** |
| Alison Lewis | Janice Birt |
| Rani Sophia | Marianne Hare |
| **Royal Bournemouth Hospital** | Saifuddin Shaik |
| Khaled Amar | **Leeds General Infirmary** |
| Rochelle Hernandez | Jane Alty |
| Alison Pitcher | Jeremy Cosgrove |
| **Royal United Hospital, Bath** | **Clinical Ageing Research Unit, Newcastle upon Tyne** |
| Samantha Carvey | David Burn |
| Ruth Hamlin | Angela Green |
| Veronica Lyell | Ann McNichol |
| **St Peters Hospital, Chertsey** | Nicola Pavese |
| Louisa Aubry | Helen Pilkington |
| Gillian Carey | Maria Price |
| Jan Coebergh | Kathryn Walker |
| **Charing Cross Hospital, London** | **Kings College Hospital, London** |
| Idah Mojela | Ray Chaudhuri |
| Sophie Molloy | Aleksandra Podlewska |
| **Royal Free Hospital, London** | Prashanth Reddy |
| Yolanda Berceruelo Bergaz | Dhaval Trivedi |
| Bintou Camera | **Royal Hallamshire Hospital, Sheffield** |
| Philip Campbell | Oliver Bandmann |
| Huw Morris | **Norfolk and Norwich Hospital** |
| Tinashe Samakomva | Vaclav Dostal |
| Anette Schrag | Jodie Graham |
| **Queen's Hospital, Romford** | Godwin Mamutse |
| Sarah Fuller | Alex Miller-Fik |
| Anjum Misbahuddin | Alison Wiltshire |
| Laura Parker | Catherine Wright |
| Elisa Visentin | **Rotherham General Hospital** |
| **John Radcliffe Hospital, Oxford** | Ahmed Abdelhafiz |
| Stephanie Gallehawk | |
| Jacqueline Rudd | |
| Sudhir Singh | |
| **Luton and Dunstable Hospital** | |
| Julie Creven | |
| Yvonne Croucher | |
| Anette Schrag | |
| Susan Tluk | |
| Paul Watts | |

© 2022 Stevens KN et al. *JAMA Neurology*.
