# Supplementary material for: Evaluation of Simvastatin as a Disease-Modifying Treatment for Patients With Parkinson Disease: A Randomized Clinical Trial
Source: JAMA Neurol. 2022 Oct 31;79(12):1232–41. doi: 10.1001/jamaneurol.2022.3718 (PMC9623477; doi:10.1001/jamaneurol.2022.3718)
Supplement: Supplement 4. — PD STAT Study Group [file jamaneurol-e223718-s004.pdf]

Supplemental Online Content: Nonauthor Collaborators
*First name, last name, and suffix (if applicable) are required and will appear in PubMed.

*Group Name(s): PD STAT Study Group							
***First Name and Middle Initial(s)**	***Last Name**	***Suffix (eg, Jr, III)**	Academic Degrees	Institution	Location (city, state/province, country)	Role or Contribution, eg, chair, principal investigator	Group (if more than 1 Group listed in the byline) and/or Subgroup (eg, Steering Committee)
Jemma	Inches			University Hospitals Plymouth	Plymouth, Devon, United Kingdom		
Donna	Underwood			University Hospitals Plymouth	Plymouth, Devon, United Kingdom		
Julie	Frost			Royal Cornwall Hospital	Truro, Cornwall, United Kingdom		
Ali	James			Royal Cornwall Hospital	Truro, Cornwall, United Kingdom		
Christine	Schofield			Royal Cornwall Hospital	Truro, Cornwall, United Kingdom		
Rob	James			Royal Devon and Exeter Hospital	Exeter, Devon, United Kingdom		
Clare	O'Reilly			Royal Devon and Exeter Hospital	Exeter, Devon, United Kingdom		
Ray	Sheridan			Royal Devon and Exeter Hospital	Exeter, Devon, United Kingdom		
Sarah	Statton			Royal Devon and Exeter Hospital	Exeter, Devon, United Kingdom		
Anita	Goff			Musgrove Park Hospital	Taunton, Somerset, United Kingdom		
Tamlyn	Russell			Musgrove Park Hospital	Taunton, Somerset, United Kingdom		
Alison	Whitcher			Musgrove Park Hospital	Taunton, Somerset, United Kingdom		
Sarah	Craw			Yeovil District Hospital	Yeovil, Somerset, United Kingdom		
Alison	Lewis			Yeovil District Hospital	Yeovil, Somerset, United Kingdom		
Rani	Sophia			Yeovil District Hospital	Yeovil, Somerset, United Kingdom		

Supplemental Online Content: Nonauthor Collaborators
*First name, last name, and suffix (if applicable) are required and will appear in PubMed.

***First Name and Middle Initial(s)**	***Last Name**	***Suffix (eg, Jr, III)**	Academic Degrees	Institution	Location (city, state/province, country)	Role or Contribution, eg, chair, principal investigator	Group (if more than 1 Group listed in the byline) and/or Subgroup (eg, Steering Committee)
Khaled	Amar			Royal Bournemouth Hospital	Bournemouth, Dorset, United Kingdom		
Rochelle	Hernandez			Royal Bournemouth Hospital	Bournemouth, Dorset, United Kingdom		
Alison	Pitcher			Royal Bournemouth Hospital	Bournemouth, Dorset, United Kingdom		
Samantha	Carvey			Royal United Hospital	Bath, Somerset, United Kingdom		
Ruth	Hamlin			Royal United Hospital	Bath, Somerset, United Kingdom		
Veronica	Lyell			Royal United Hospital	Bath, Somerset, United Kingdom		
Louisa	Aubry			St Peters Hospital	Chertsey, Surrey, United Kingdom		
Gillian	Carey			St Peters Hospital	Chertsey, Surrey, United Kingdom		
Jan	Coebergh			St Peters Hospital	Chertsey, Surrey, United Kingdom		
Idah	Mojela			Charing Cross Hospital	London, Greater London, United Kingdom		
Sophie	Molloy			Charing Cross Hospital	London, Greater London, United Kingdom		
Yolanda	Berceruelo Bergaz			Royal Free Hospital	London, Greater London, United Kingdom		
Bintou	Camera			Royal Free Hospital	London, Greater London, United Kingdom		
Philip	Campbell			Royal Free Hospital	London, Greater London, United Kingdom		
Huw	Morris			Royal Free Hospital	London, Greater London, United Kingdom		
Tinashe	Samakomva			Royal Free Hospital	London, Greater London, United Kingdom		

Supplemental Online Content: Nonauthor Collaborators
*First name, last name, and suffix (if applicable) are required and will appear in PubMed.

***First Name and Middle Initial(s)**	***Last Name**	***Suffix (eg, Jr, III)**	Academic Degrees	Institution	Location (city, state/province, country)	Role or Contribution, eg, chair, principal investigator	Group (if more than 1 Group listed in the byline) and/or Subgroup (eg, Steering Committee)
Anette	Schrag			Royal Free Hospital	London, Greater London, United Kingdom		
Sarah	Fuller			Queen's Hospital	Romford, Greater London, United Kingdom		
Anjum	Misbahuddin			Queen's Hospital	Romford, Greater London, United Kingdom		
Laura	Parker			Queen's Hospital	Romford, Greater London, United Kingdom		
Elisa	Visentin			Queen's Hospital	Romford, Greater London, United Kingdom		
Stephanie	Gallehawk			John Radcliffe Hospital	Oxford, Oxfordshire, United Kingdom		
Jacqueline	Rudd			John Radcliffe Hospital	Oxford, Oxfordshire, United Kingdom		
Sudhir	Singh			John Radcliffe Hospital	Oxford, Oxfordshire, United Kingdom		
Sarsha	Wilson			John Radcliffe Hospital	Oxford, Oxfordshire, United Kingdom		
Julie	Creven			Luton and Dunstable University Hospital	Luton, Bedfordshire, United Kingdom		
Yvonne	Croucher			Luton and Dunstable University Hospital	Luton, Bedfordshire, United Kingdom		
Anette	Schrag			Luton and Dunstable University Hospital	Luton, Bedfordshire, United Kingdom		
Susan	Tluk			Luton and Dunstable University Hospital	Luton, Bedfordshire, United Kingdom		
Paul	Watts			Luton and Dunstable University Hospital	Luton, Bedfordshire, United Kingdom		
Simone	Hargreaves			Addenbrooke's Hospital	Cambridge, Cambridgeshire, United Kingdom		

Supplemental Online Content: Nonauthor Collaborators
*First name, last name, and suffix (if applicable) are required and will appear in PubMed.

***First Name and Middle Initial(s)**	***Last Name**	***Suffix (eg, Jr, III)**	Academic Degrees	Institution	Location (city, state/province, country)	Role or Contribution, eg, chair, principal investigator	Group (if more than 1 Group listed in the byline) and/or Subgroup (eg, Steering Committee)
Danielle	Johnson			Addenbrooke’s Hospital	Cambridge, Cambridgeshire, United Kingdom		
Lucy	Worboys			Addenbrooke’s Hospital	Cambridge, Cambridgeshire, United Kingdom		
Paul	Worth			Addenbrooke’s Hospital	Cambridge, Cambridgeshire, United Kingdom		
Judith	Brooke			Salford Royal Hospital	Salford, Greater Manchester, United Kingdom		
Christopher	Kobylecki			Salford Royal Hospital	Salford, Greater Manchester, United Kingdom		
Victoria	Parker			Salford Royal Hospital	Salford, Greater Manchester, United Kingdom		
Judith	Brooke			Fairfield General Hospital	Bury, Greater Manchester, United Kingdom		
Linda	Johnson			Fairfield General Hospital	Bury, Greater Manchester, United Kingdom		
Rosane	Joseph			Fairfield General Hospital	Bury, Greater Manchester, United Kingdom		
Julie	Melville			Fairfield General Hospital	Bury, Greater Manchester, United Kingdom		

Supplemental Online Content: Nonauthor Collaborators
*First name, last name, and suffix (if applicable) are required and will appear in PubMed.

***First Name and Middle Initial(s)**	***Last Name**	***Suffix (eg, Jr, III)**	Academic Degrees	Institution	Location (city, state/province, country)	Role or Contribution, eg, chair, principal investigator	Group (if more than 1 Group listed in the byline) and/or Subgroup (eg, Steering Committee)
Jason	Raw			Fairfield General Hospital	Bury, Greater Manchester, United Kingdom		
Janice	Birt			Royal Preston Hospital	Preston, Lancashire, United Kingdom		
Marianne	Hare			Royal Preston Hospital	Preston, Lancashire, United Kingdom		
Saifuddin	Shaik			Royal Preston Hospital	Preston, Lancashire, United Kingdom		
Jane	Alty			Leeds General Infirmary	Leeds, West Yorkshire, United Kingdom		
Jeremy	Cosgrove			Leeds General Infirmary	Leeds, West Yorkshire, United Kingdom		
David	Burn			Clinical Ageing Research Unit, Newcastle University	Newcastle upon Tyne, Tyne and Wear, United Kingdom		
Angela	Green			Clinical Ageing Research Unit, Newcastle University	Newcastle upon Tyne, Tyne and Wear, United Kingdom		
Ann	McNichol			Clinical Ageing Research Unit, Newcastle University	Newcastle upon Tyne, Tyne and Wear, United Kingdom		
Nicola	Pavese			Clinical Ageing Research Unit, Newcastle University	Newcastle upon Tyne, Tyne and Wear, United Kingdom		
Helen	Pilkington			Clinical Ageing Research Unit, Newcastle University	Newcastle upon Tyne, Tyne and Wear, United Kingdom		
Maria	Price			Clinical Ageing Research Unit, Newcastle University	Newcastle upon Tyne, Tyne and Wear, United Kingdom		

Supplemental Online Content: Nonauthor Collaborators
*First name, last name, and suffix (if applicable) are required and will appear in PubMed.

***First Name and Middle Initial(s)**	***Last Name**	***Suffix (eg, Jr, III)**	Academic Degrees	Institution	Location (city, state/province, country)	Role or Contribution, eg, chair, principal investigator	Group (if more than 1 Group listed in the byline) and/or Subgroup (eg, Steering Committee)
Kathryn	Walker			Clinical Ageing Research Unit, Newcastle University	Newcastle upon Tyne, Tyne and Wear, United Kingdom		
Ray	Chaudhuri			King's College Hospital	London, Greater London, United Kingdom		
Aleksandra	Podlewska			King's College Hospital	London, Greater London, United Kingdom		
Prashanth	Reddy			King's College Hospital	London, Greater London, United Kingdom		
Dhaval	Trivedi			King's College Hospital	London, Greater London, United Kingdom		
Oliver	Bandmann			Royal Hallamshire Hospital	Sheffield, South Yorkshire, United Kingdom		
Rosie	Clegg			Royal Hallamshire Hospital	Sheffield, South Yorkshire, United Kingdom		
Grace	Cole			Royal Hallamshire Hospital	Sheffield, South Yorkshire, United Kingdom		
Anna	Emery			Royal Hallamshire Hospital	Sheffield, South Yorkshire, United Kingdom		
Vaclav	Dostal			Norfolk and Norwich Hospital	Norwich, Norfolk, United Kingdom		
Jodie	Graham			Norfolk and Norwich Hospital	Norwich, Norfolk, United Kingdom		
Jocelyn	Keshet-Price			Norfolk and Norwich Hospital	Norwich, Norfolk, United Kingdom		
Godwin	Mamutse			Norfolk and Norwich Hospital	Norwich, Norfolk, United Kingdom		

Supplemental Online Content: Nonauthor Collaborators
*First name, last name, and suffix (if applicable) are required and will appear in PubMed.

***First Name and Middle Initial(s)**	***Last Name**	***Suffix (eg, Jr, III)**	Academic Degrees	Institution	Location (city, state/province, country)	Role or Contribution, eg, chair, principal investigator	Group (if more than 1 Group listed in the byline) and/or Subgroup (eg, Steering Committee)
Alex	Miller-Fik			Norfolk and Norwich Hospital	Norwich, Norfolk, United Kingdom		
Alison	Wiltshire			Norfolk and Norwich Hospital	Norwich, Norfolk, United Kingdom		
Catherine	Wright			Norfolk and Norwich Hospital	Norwich, Norfolk, United Kingdom		
Kathryn	Dixon			Rotherham General Hospital	Rotherham, South Yorkshire, United Kingdom		
Ahmed	Abdelhafiz			Rotherham General Hospital	Rotherham, South Yorkshire, United Kingdom		
Joanne	Rose			Rotherham General Hospital	Rotherham, South Yorkshire, United Kingdom		
