# Supplementary material for: Evaluation of Simvastatin as a Disease-Modifying Treatment for Patients With Parkinson Disease: A Randomized Clinical Trial
Source: JAMA Neurol. 2022 Oct 31;79(12):1232–41. doi: 10.1001/jamaneurol.2022.3718 (PMC9623477; doi:10.1001/jamaneurol.2022.3718)
Supplement: Supplement 5. — Data Sharing Statement [file jamaneurol-e223718-s005.pdf]

Data Sharing Statement

Stevens. Evaluation of Simvastatin as a Neuroprotective Treatment for Patients With Parkinson Disease. *JAMA Neurol*. Published October 31, 2022. doi:10.1001/jamaneurol.2022.3718

Data
Data available: Yes
Data types: Deidentified participant data
How to access data: By request to the data custodian/sponsor representative (crollinson@nhs.net).
When available: With publication

Supporting Documents
Document types: None

Additional Information
Who can access the data: Researchers undertaking ethically approved purposes and on execution of a valid data sharing agreement.
Types of analyses: Specified ethically approved purpose.
Mechanisms of data availability: With a signed valid data sharing agreement.
